# A guided network estimation approach using multi-omic information

**DOI:** 10.1186/s12859-024-05778-7

**Published:** 2024-05-30

**Authors:** Georgios Bartzis, Carel F. W. Peeters, Wilco Ligterink, Fred A. Van Eeuwijk

**Affiliations:** 1 Mathematical and Statistical Methods Group - Biometris, Wageningen University and Research, Wageningen, The Netherlands; 2 Laboratory of Plant Physiology, Wageningen University and Research, Wageningen, The Netherlands

**Keywords:** Multi-omics, Network reconstruction, Network integration

## Abstract

**Intoduction:**

In systems biology, an organism is viewed as a system of interconnected molecular entities. To understand the functioning of organisms it is essential to integrate information about the variations in the concentrations of those molecular entities. This information can be structured as a set of networks with interconnections and with some hierarchical relations between them. Few methods exist for the reconstruction of integrative networks.

**Objective:**

In this work, we propose an integrative network reconstruction method in which the network organization for a particular type of omics data is guided by the network structure of a related type of omics data upstream in the omic cascade. The structure of these guiding data can be either already known or be estimated from the guiding data themselves.

**Methods:**

The method consists of three steps. First a network structure for the guiding data should be provided. Next, responses in the target set are regressed on the full set of predictors in the guiding data with a Lasso penalty to reduce the number of predictors and an L2 penalty on the differences between coefficients for predictors that share edges in the network for the guiding data. Finally, a network is reconstructed on the fitted target responses as functions of the predictors in the guiding data. This way we condition the target network on the network of the guiding data.

**Conclusions:**

We illustrate our approach on two examples in Arabidopsis. The method detects groups of metabolites that have a similar genetic or transcriptomic basis.

**Supplementary Information:**

The online version contains supplementary material available at 10.1186/s12859-024-05778-7.

## Introduction

Advances in high-throughput technology have enabled the massive collective quantification of molecular entities, such as messenger RNA, proteins, and metabolites. This age of omics has revolutionalized the field of systems biology, enabling biological systems to be studied using mathematical and computational modeling on high-dimensional omics data. In systems biology, an organism is viewed as a complex web of interacting molecular entities [19] studied in order to outline how cells, organs, and tissues behave at a molecular level [21].

A commonly used tool for analyzing omics data is network analysis. In network analysis, each omics level is assumed to have a network representation where complex associations are visualized by graphical structures. SNPs, genes, metabolites, and/or traits are typically represented by nodes in a graph and their associations (physical, genetic, or functional) by edges connecting them. Extracted patterns are then used to help elucidate biological mechanisms underlying traits of interest.

### Methods for omic data integration

A key question in systems biology is how to model omics data at a systems level (integrative analysis), instead of each omics source separately [12]. Several approaches have been developed in the context of integrated analysis, see [5, 11]. One such approach for two sets of omics data is canonical correlation analysis (CCA) [8]. In order to solve CCA, the inverse of two covariance matrices needs to be computed which is problematic when the number of variables exceeds the number of samples, therefore penalization techniques can be implemented. Similarly, penalized partial least squares (PLS) regression [13] variants (sPLS; sparse Partial Least Squares) have been proposed in order to remove noisy variables resulting in variable selection for both sets of omics data [14].

An extension of sPLS is the sparse multi block partial least squares regression (sMBPLS) [16] in which several genomic data are measured on the same samples. One dataset is considered the response data, while the rest acts as guiding sets. In an application using a dataset containing gene expression (response data), copy number variation, DNA methylation, and micro RNA expression, Li and et al. [16] identified combinations of multiple types of genomic markers that jointly impacted the expression of a set of genes. The covariance between the data blocks and the response block is maximized so that multidimensional modules are discovered associating the guiding with the response data.

Finally, network-based integration methods have also been proposed. The integration may be vertical (across omic-levels) or horizontal (one omic platform through time). The vertical approaches aim to provide a mechanistic understanding of molecular (de)regulation across the omic cascade. An overview of such methods can be found in [1]. In this work, we propose an integrative network reconstruction method where the network topology of one type of omics data is conditioned on the network topology of another omics source that is upstream in the omics cascade [4].

### Aim

The question answered in this work is how to integrate information across multiple omics levels. To answer and better understand relationships between different biological functional levels, we need to combine a systems view (requiring network modeling) and a multimodal view (requiring data integration).

In this work, we study whether network reconstruction of a particular omics source can benefit from information from the network organization of another omics source. For example, is metabolite network reconstruction helped by using DNA information? Or does information on a gene expression network aid recovery of the metabolites’ network organization? Under our setting, for *N* samples, there are two sets of omics data; the *P*-dimensional target dataset (denoted by ${\varvec{Y}}_{N\times P}$ from hereon) for which the underlying network organization needs to be recovered by using a *Q*-dimensional guiding dataset (denoted by ${\varvec{X}}_{N\times Q}$ from hereon) and information on its network structure which is represented by a $Q\times Q$ matrix.

For estimating the network organization of the target data using the *guiding* data set and its network organization, a guided network estimation approach is considered. First, the network organization of the guiding data is estimated. We then regress the target on the guiding data and keep the fitted values on which we estimate a network structure. Alternatively, a guiding network can be used that is available already.

### Overview

The rest of the paper is organized as follows. In Sect. 2, we review some basic network concepts, and propose a guided approach for estimating the network organization of an omics source using information from another omic dataset. In Sect. 3, we demonstrate our approach on metabolite data coming from the *Arabidopsis thaliana* population.

In the first application, the metabolic network estimation is guided by utilizing SNP information. SNP data and their spatial organization are used as input (DNA structure can be seen as a linear network, with edge intensity analogous to the distance between the markers on the chromosome) [2]. We then identify and retain the part of metabolic variation related to SNP information and its structure and use it for estimating networks of metabolites. In the second data example, we guide the estimation of the metabolite networks by using information coming from gene-expression data and their network organization. Pairs of metabolites will share edges if they are associated to similar gene sets. Here, the data come from the Wageningen Seed Lab and contain SNP, transcriptomic, and metabolic information [10]. We consider this to be a standard dataset and demonstrate our integrative network approach. Our aim is to understand the metabolites from a SNP and gene level. Using network analysis we detect groups of metabolites having similar genetic or transcriptomic basis. We conclude the article with some discussion in Sect. 4.

## Methods

Network analysis is a multivariate type of analysis aimed at recovering the underlying network structure of the data. We consider networks a representation of the pairwise (conditional) (in)dependencies between random variables. The nodes then represent metabolites or other molecular features and the edges represent pairwise dependency. An undirected network is typically encoded into a symmetric matrix ${\varvec{W}}$ (intensity matrix). The element $w_{ij}$ can be any type of association measure, e.g., the (absolute) partial correlation or (absolute) marginal correlation coefficient. The row- or column-sum of ${\varvec{W}}$ is called strength and measures the total intensity of the connections of node *i*: $s({\varvec{X}})_i=\sum _j {\varvec{W}}({\varvec{X}})_{ij}$.

### Graphical LASSO

A popular approach for obtaining the underlying structure of the data from a set of *P* correlated variables (measured in *N* samples) is the Graphical LASSO (GL). In GL, the observational vectors of the data ${\varvec{Z}}_{N\times P}$, where ${\varvec{Z}}$ denotes a general dataset (either guiding or target data), are assumed to follow a *P*-dimensional multivariate normal distribution with mean vector ${\varvec{0}}$ and variance-covariance matrix $\varvec{\Sigma }$. GL is based on the conditional independence of pairwise relationships, meaning that the precision matrix ($\varvec{\Theta }=\varvec{\Sigma }^{-1}$) is estimated. When the element $\theta _{ij}$ is equal to zero, variables *i* and *j* are conditionally independent given all other variables. The penalized log likelihood using a LASSO penalty [6, 7] is:

1 $$\begin{aligned} \ell _\lambda (\varvec{\Theta })\propto \log |\varvec{\Theta } |-\hbox {tr}({\varvec{S}}\varvec{\Theta })-\lambda ||\varvec{\Theta }||_1, \end{aligned}$$

where $||\bullet ||_1$ is the *L*1-norm and $\lambda$ is a non-negative tuning parameter governing the sparsity of the estimated precision matrix $\hat{\varvec{\Theta }}$. The tuning parameter $\lambda$ can be chosen based on subsampling. Here, we use the Stability Approach to Regularization Selection (StARS) [17] to estimate a set of stable edges. When using StARS, sparse networks are estimated based on multiple overlapping subsamples of the data, for different $\lambda$ values on a grid. For an optimal $\lambda$ (resulting in a sparse and stable network under random subsampling) selected by StARS, the absolute estimated precision matrix (similar to [26]) $|\hat{\varvec{\Theta }}|$ will be used here as the intensity matrix $\hat{{\varvec{W}}}({\varvec{Z}})$.

### Visual representation

To visually represent the sparse precision matrix as a network, the *P* variables are represented as a set of *P* nodes/vertices, which are connected by a set of edges, dictated by the non-zero entries of ${\varvec{W}}({\varvec{Z}})$. The intensity of the connections between variables can be visualized by edge thickness with wider edges representing stronger connections. By taking the optimal $\lambda$ selected by StARS as fixed, the intensity matrix $\hat{{\varvec{W}}}({\varvec{Z}})_t=|\hat{\varvec{\Theta }_t}|$ can be computed based on different subsamples $t=\{1,\ldots ,T\}$. The edge-wise standard deviation computed over all $\hat{{\varvec{W}}}({\varvec{Z}})_t$ can be an indicator of the edge’s uncertainty. Since a network is a visual representation of an intensity matrix, we will be using both terms interchangeably and denote a network by its estimated intensity matrix $\hat{{\varvec{W}}}({\varvec{Z}})$.

### From guiding to target data

Let ${\varvec{Y}}=\{{\varvec{y}}_1,\ldots , {\varvec{y}}_P\}$ be the $N\times P$ target omics data matrix. Further, assume that ${\varvec{X}}=\{{\varvec{x}}_1,\ldots ,{\varvec{x}}_Q\}$ is the $N\times Q$ guiding omics data matrix. If ${\varvec{Y}}$ contains the concentration levels of *P* metabolites on *N* samples, ${\varvec{X}}$ could contain, for those same samples, data from *Q* SNPs or the expression levels of *Q* genes.

To incorporate information from the guiding omics data into the analysis of the target data we work in a regression framework. Prior to any type of multivariate analysis, e.g. network analysis, each of the *P* variables of ${\varvec{Y}}$ is regressed on all *Q* variables of ${\varvec{X}}$. Subsequently, the fitted values $\hat{{\varvec{Y}}}({\varvec{X}})$ are obtained, e.g. using penalized regression [23]. Note that the OLS coefficient estimates cannot be obtained in the high dimensional case $Q>N$. LASSO regression has some attractive properties by performing variable selection, i.e. leading to zero coefficients for some of the variables. On the other hand, no information on the dependencies between ${\varvec{X}}$ variables ($\hat{{\varvec{W}}}({\varvec{X}})$) is used.

This drawback can be alleviated by using network-constrained regularization (NCR), as proposed by [15], where the underlying network organization of ${\varvec{X}}$ is explicitly modeled when regressing each of the *P* variables of ${\varvec{Y}}$ on ${\varvec{X}}$ [2].

### Network constrained regularization

We first assume that the data ${\varvec{X}}$ have an underlying estimable network organization $\hat{{\varvec{W}}}({\varvec{X}})$. For the $p^{th}$ response (${\varvec{y}}_p$), the estimated regression coefficients $\hat{\varvec{\beta }}_p \in {\varvec{R}}^{Q \times 1}$ are obtained as:

2 $$\begin{aligned}\hat{\varvec{\beta }}_p= &\mathop {\mathrm {arg\,min}}\limits _{\varvec{\beta }_p}\Bigg \lbrace ({\varvec{y}}_p -{\varvec{X}}\varvec{\beta }_p)^\top ({\varvec{y}}_p-{\varvec{X}}\varvec{\beta }_p) +\lambda _1||\varvec{\beta }_p||_1+ \Bigg .\nonumber \\& \, \Bigg .\lambda _2\sum _{i\sim j}\left( \dfrac{\beta _{p_i}}{\sqrt{s({\varvec{X}})_i}}-\dfrac{\beta _{p_j}}{\sqrt{s({\varvec{X}})_j}} \right) ^2\hat{{\varvec{W}}}({\varvec{X}})_{ij}\Bigg \rbrace , \end{aligned}$$

where $\sum _{i\sim j}$ denotes the sum over all adjacent unordered *ij* pairs, $s({\varvec{X}})_i$, $s({\varvec{X}})_j$ are the strengths of nodes *i* and *j*, and the term $\lambda _1||\bullet ||_1$ is a LASSO-type penalty inducing a sparse solution in which not all *Q* predictors enter the model. For selecting the penalty parameters, cross-validation (CV) can be used for estimating the prediction error for set values of $\lambda _1$ and $\lambda _2$. The chosen penalties are the ones giving the lowest CV error (in our applications we used 5-fold CV). Note that (2) can be seen as a generalization of the elastic net [15].

The part accounting for the network structure of ${\varvec{X}}$ when estimating $\hat{\varvec{\beta }}_p$ in (2) is:

3 $$\begin{aligned} \lambda _2\sum _{i\sim j}\left( \dfrac{\beta _{p_i}}{\sqrt{s(X)_i}}-\dfrac{\beta _{p_j}}{\sqrt{s({\varvec{X}})_j}} \right) ^2\hat{{\varvec{W}}}({\varvec{X}})_{ij}. \end{aligned}$$

The regression coefficients $\varvec{\beta }_p$ are smoothed by penalizing the sum of weighted squares of the differences between $\beta _{p_i}$ and $\beta _{p_j}$. Therefore, when nodes *i* and *j* share an edge with some weight ($w({\varvec{X}})_{ij}\ne 0$) in the network of ${\varvec{X}}$, they will tend to have similar association to ${\varvec{y}}_p$. This can be biologically justified since it is expected that connected nodes (in the case of SNPs/genes/metabolites) have similar function [15] and subsequently their coefficients should be shrunken towards each other. In expression (3) it can be seen that the regression coefficients are scaled, since it is expected that nodes with higher strength are more important and therefore have larger coefficients.

The linear model using the NCR criterion, unlike LASSO, preserves the grouping property, meaning that groups of connected variables (predictors linked in $\hat{{\varvec{W}}}({\varvec{X}})$) will enter the model together. This result is shown in [15].

We then fit values of the target responses on the guiding predictors ${\varvec{X}}$:

4 $$\begin{aligned} \hat{{\varvec{y}}}_p({\varvec{X}})={\varvec{X}}\hat{\varvec{\beta }}_p, \end{aligned}$$

for each p. These are used for network reconstruction on $\hat{{\varvec{Y}}}$.

### Three-step approach for network reconstruction

For recovering the network structure of the target omics data, i.e. ${\varvec{Y}}$ using a guiding omics source ${\varvec{X}}$, we thus have a general 3-step approach:

1. Represent the guiding structure with an estimated or a priori known intensity matrix, i.e. $\hat{{\varvec{W}}}({\varvec{X}})$ using GL;
2. Evaluate expression (2) with ${\varvec{Y}}$, ${\varvec{X}}$, and $\hat{{\varvec{W}}}(\varvec{{\varvec{X}}})_{ij}$ and retain the fitted data matrix $\hat{{\varvec{Y}}}({\varvec{X}})$;
3. Use $\hat{{\varvec{Y}}}({\varvec{X}})$ to reconstruct the target intensity matrix $\hat{{\varvec{W}}}(\hat{{\varvec{Y}}}({\varvec{X}}))$ using GL.

By using the proposed multi-step approach, the two omics sources are no longer treated independently. The resulting estimated network of the target data is conditioned on the network organization of the guiding data.

## Application to data

We now use the proposed methods for estimating metabolite networks while using information from other omics sources that have a network organization of their own.

We use a Recombinant Inbred Line (RIL) population of a cross between two Arabidopsis accessions, i.e. Bayreuth (Bay-0) and Shahdara (Sha). In this population we want to study the metabolite similarities subject to variation coming from lower leveled omics sources (SNPs or Genes). In our first example we utilize SNP data and their spatial relationship to estimate metabolite networks. Metabolites will be connected if they have the same genetic basis (similar QTLs). In the second example, we use gene expression data and their underlying network organization information when we estimate metabolite networks. Therefore, we identify metabolites with similar transcriptomic basis.

### Data

Seeds from 164 lines of the Arabidopsis Bay-0$\times$Sha RIL population were divided into four sub-populations (41 lines each) representing four important developmental stages of seed germination; (1) freshly harvested primary dormant dry seeds (PD), (2) after-ripened non-dormant dry seeds, (3) seeds imbibed for 6 h (6 H), and (4) seeds at radical protrusion (RP).

For determining the metabolite concentrations, all 164 lines were subjected to gas chromatography time of flight mass spectrometry giving 7537 peaks, representing 161 metabolites based on retention time and correlation structure [10, 24]. In total, $P=64$ metabolites were annotated and were further used in our analysis. Gene expression analysis was performed using the Affymetrix AtSNPtile microarray on the same sub-populations and developmental stages as the metabolites, where the expression levels of 29304 genes were extracted. The top 10$\%$ most varying genes ($Q1=2931$ genes) were retained for further analysis. Concentration levels of the metabolites and gene expression levels were log transformed and adjusted for the four developmental seed stages by subtracting the mean levels from each group. Finally, information on $Q2=1059$ markers (5 chromosomes) was available. More information on the study design and data can be found in [10] and [9]. For the rest of the paper, since metabolites will be the target dataset, we will denote their $N\times P$ data matrix as ${\varvec{Y}}=\{{\varvec{y}}_1,\ldots ,{\varvec{y}}_{P}\}$. The $N\times Q1$ gene expression data and the $N\times Q2$ SNP data matrix will be used as guiding dataset and will be denoted as ${\varvec{X}}^G=\{{\varvec{x}}^G_1,\ldots ,{\varvec{x}}^G_{Q1}\}$ and ${\varvec{X}}^S=\{{\varvec{x}}^S_1,\ldots ,{\varvec{x}}^S_{Q2}\}$, respectively.

### From SNPs to metabolites

#### Step 1: The SNP network representation

By having map information known, we represent the SNP data as the simplest type of network, i.e. a one-dimensional linear 'network’. We represent with $\alpha _{(1)},\ldots ,\alpha _{(p)}$ the ordered (in ascending order) genetic/physical position of the markers on the chromosome. The intensity of the connections between neighboring nodes is the relative (genetic/physical) marker proximity is calculated as:

5 $$\begin{aligned} \hat{{\varvec{W}}}({\varvec{X}}^S)_{ij} = \hat{{\varvec{W}}}({\varvec{X}}^S)_{ji}= 1-\dfrac{\alpha _{(j)}-\alpha _{(i)}}{\alpha _{(p)}-\alpha _{(1)}}, \text{ where } i=1,\ldots ,p-1 \text{ and } j=i+1, \end{aligned}$$

where $\hat{{\varvec{W}}}({\varvec{X}}^S)_{ij}=0$ for all other cases and for markers belonging to different chromosomes.

#### Step 2: Estimating the metabolite part related to genetic variation

In order to use ${\varvec{X}}^S$, and ${\varvec{W}}({\varvec{X}}^S)$ for estimating ${\varvec{Y}}^{M}({\varvec{X}}^S)$, we work with the NCR as described in Sect. 2.3. Sets of SNPs that relate to each metabolite are identified. For metabolite *p*, the vector of coefficients $\varvec{\beta }^{S}_{p}$ is estimated and used for obtaining the metabolite fitted values as:

$$\begin{aligned} \hat{{\varvec{y}}}_{p}^{M}({\varvec{X}}^S)={\varvec{X}}^S\hat{\varvec{\beta }}^{S}_{p}. \end{aligned}$$

#### Step 3: Estimating metabolite network related to genetic variation

**Fig. 1:**
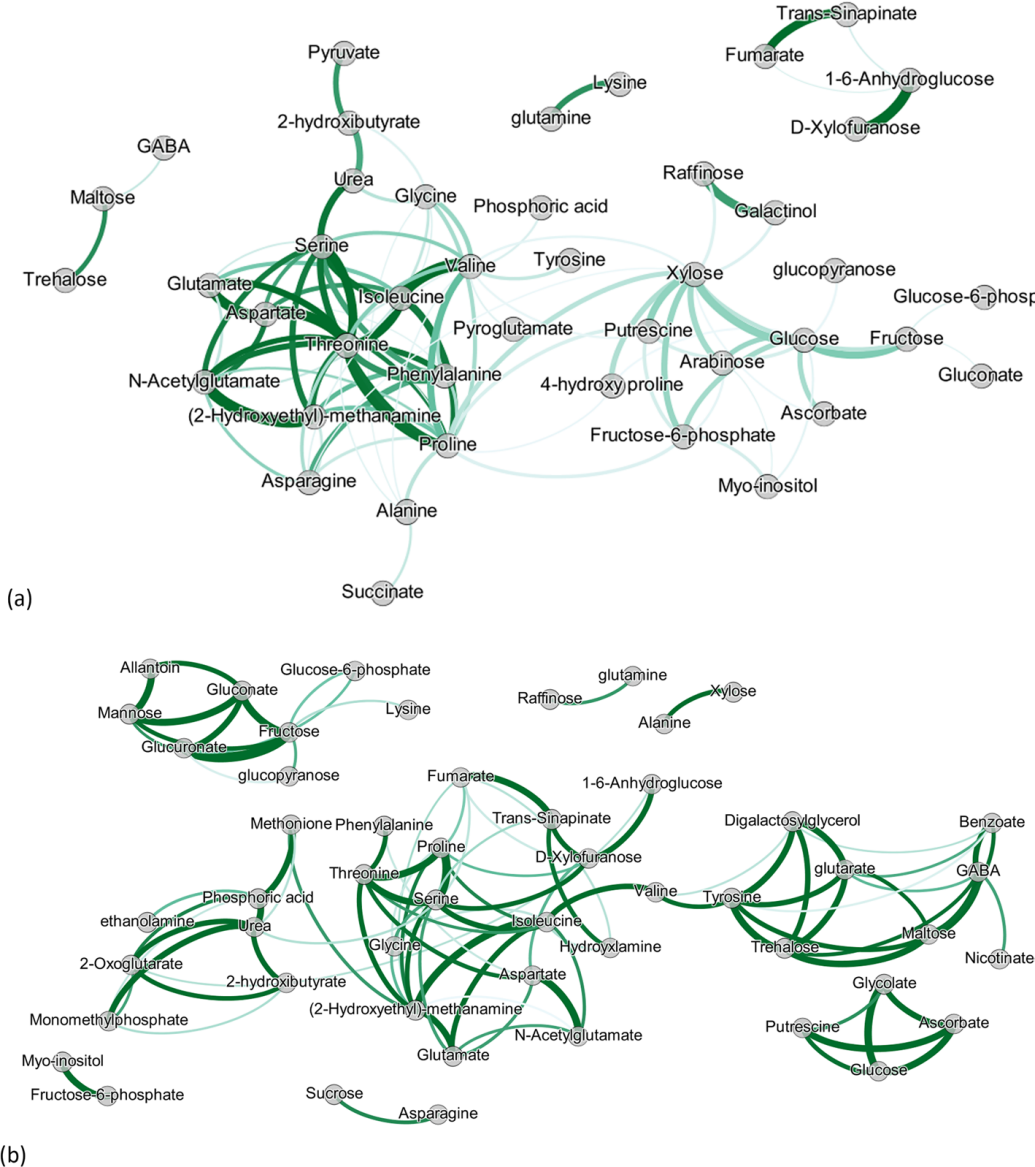
Estimated metabolite networks when: (**a**) using the original metabolite data (${\varvec{W}}({\varvec{Y}}^{M})$), and (**b**) using information on SNPs and their network structure (${\varvec{W}}(\hat{{\varvec{Y}}}^{M}({\varvec{X}}^S))$). Edges’ width denotes the intensity of the connection between two nodes, while edges’ opacity indicates the uncertainty as measured by the edges' standard deviation

By using GL coupled with StARS on $\hat{{\varvec{Y}}}^{M}({\varvec{X}}^S)$, the metabolite network using SNP information $\hat{{\varvec{W}}}(\hat{{\varvec{Y}}}^{M}({\varvec{X}}^S))$ was estimated and is visualized in Fig. 1. The optimal regularization parameter $\lambda ^S$ equalled 0.651, resulting in 98 edges between the metabolites. In the same figure, the network using the original metabolite values (${\varvec{Y}}^{M}$), i.e. $\hat{{\varvec{W}}}({\varvec{Y}}^{M})$ is depicted. In order to compare the two networks, we controlled the sparsity of $\hat{{\varvec{W}}}({\varvec{Y}}^{M})$: select the regularization parameter giving the same number of edges (98 out of 2016 possible edges resulting in sparsity of 0.049). Therefore, the tuning parameter governing the network sparsity in $\hat{{\varvec{W}}}({\varvec{Y}}^{M})$ was selected to be 0.554.

#### Results and comparison

By examining Fig. 1, we first see that the uncertainty of the edges is lower in $\hat{{\varvec{W}}}(\hat{{\varvec{Y}}}^{M}({\varvec{X}}^S))$ compared to $\hat{{\varvec{W}}}({\varvec{Y}}^{M})$. The top connected (hub-nodes) metabolites in $\hat{{\varvec{W}}}({\varvec{Y}}^{M})$ are *Proline, Valine, Threonine, Xylose*, and *Serine* with 16, 13, 12, 12, and 10 edges respectively. On the other hand, when we see the network of metabolites with respect to SNP variation, the top connected metabolites are *Serine, (2-Hydroxyethyl)-methanamine, Isoleucine*, and *Proline* with 10, 9, 9, and 7 edges, respectively.

**Fig. 2:**
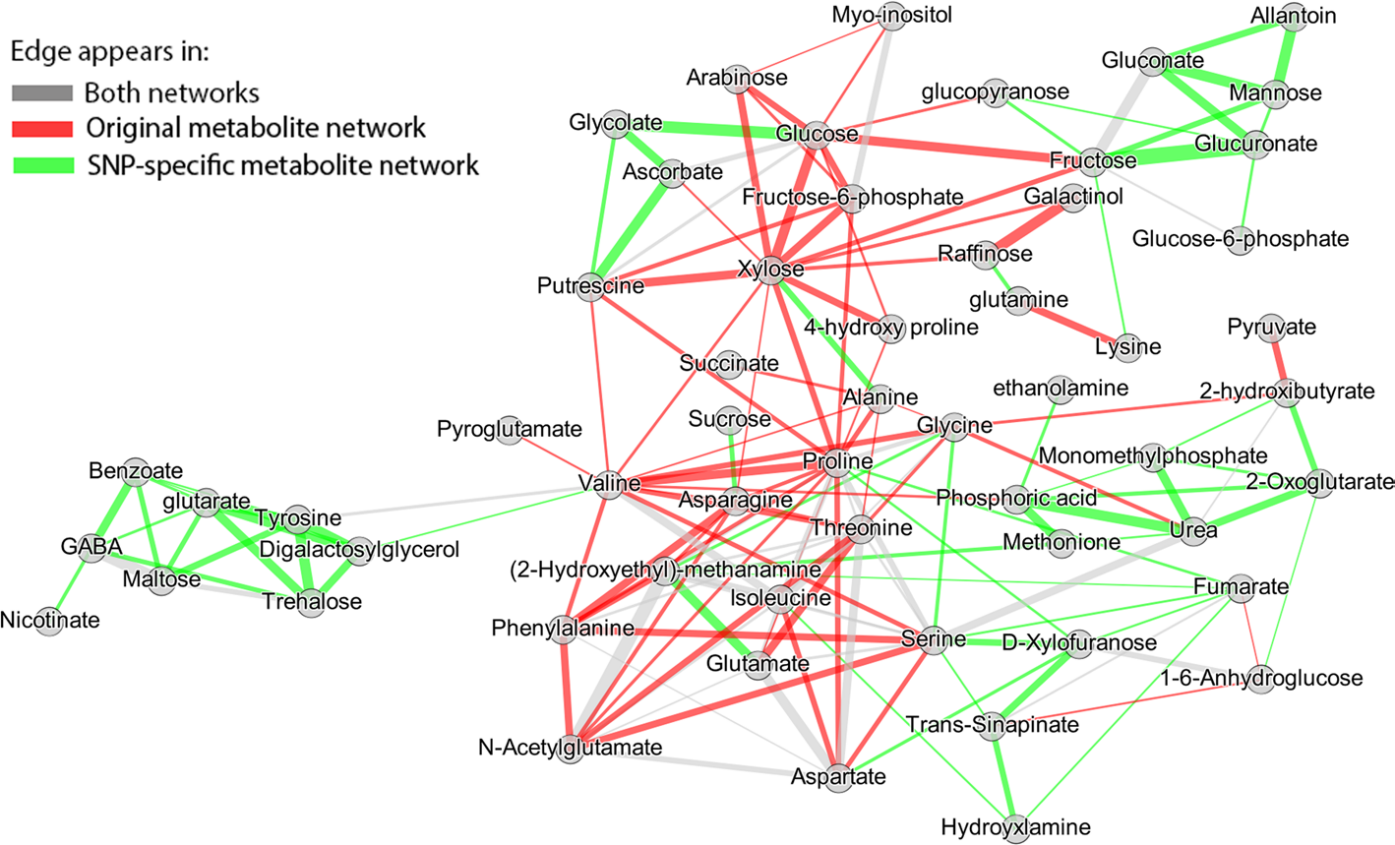
Difference between network based on the original metabolite values (${\varvec{W}}({\varvec{Y}}^{M})$) and network reconstructed when SNP information is used (${\varvec{W}}(\hat{{\varvec{Y}}}^{M}({\varvec{X}}^S))$). Green edges denote the unique edges that appear in ${\varvec{W}}(\hat{{\varvec{Y}}}^{M}({\varvec{X}}^S))$. Red denote the unique edges appearing in ${\varvec{W}}({\varvec{Y}}^{M})$. Grey edges are the common edges between ${\varvec{W}}(\hat{{\varvec{Y}}}^{M}({\varvec{X}}^S))$ and ${\varvec{W}}({\varvec{Y}}^{M})$. The width of the edges denotes the difference between the connections’ intensity of ${\varvec{W}}(\hat{{\varvec{Y}}}^{M}({\varvec{X}}^S))$ and ${\varvec{W}}({\varvec{Y}}^{M})$

Here, we highlight the major differences between the networks by comparing them. Differences between the networks are visualized in Fig. 2. Edges are colored with green if they only appear in $\hat{{\varvec{W}}}(\hat{{\varvec{Y}}}^{M}({\varvec{X}}^S))$, red if they only appear in $\hat{{\varvec{W}}}({\varvec{Y}}^{M})$, and grey if they appear in both.

Interestingly, the metabolite losing the most edges by conditioning on SNP information (12) is *Xylose*, showing that the similarity with other metabolites was due to the non-genetic variation. Other metabolites losing multiple edges when we use SNP information are: *Proline* (11), *Valine *(11), *Asparagine *(7), and *Glucose* (7).

On the other hand, the metabolites that gained multiple edges by conditioning on SNP information are *Glutarate* (with 7) and *2-Oxoglutarate, Benzoate, Digalactosylglycerol, D-Xylofuranose, Fumarate, Glucuronate, Phosphoric acid*, and *Tyrosine* (with 5 edges each), showing that their genetic similarity with other metabolites was stronger but it was concealed by their non-genetic variation part.

Finally, the top metabolites retaining many edges are: *Isoleucine* (8), *Serine* (6), *Threonine* (6), *(2-Hydroxyethyl)-methanamine* (5), and *Proline* (5) showing that their genetic similarity with other metabolites was stronger than the non-genetic.

#### Connection between QTLs and metabolite network

**Fig. 3:**
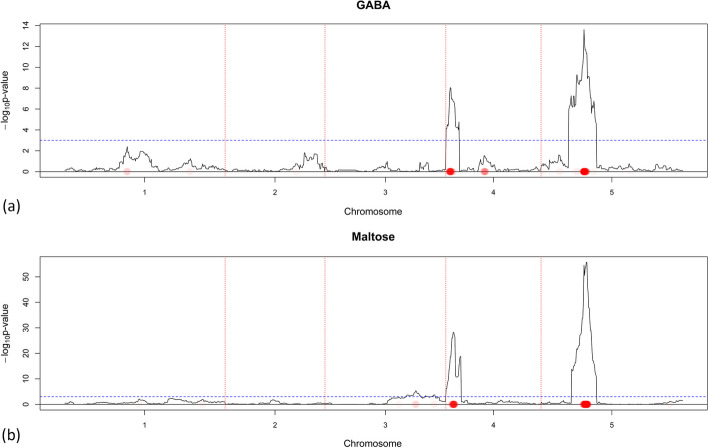
QTL detection for GABA (**a**) and Maltose (**b**) using CIM and NCR when the guiding dataset is SNP data. GABA and Maltose share an edge in both ${\varvec{W}}(\hat{{\varvec{Y}}}^{M}({\varvec{X}}^S))$ and $\hat{{\varvec{W}}}({\varvec{Y}}^{M})$ which can be justified by their QTL profile. The $-\log _{10}$ *p*-value score for every marker is plotted when CIM is used. The red dotted vertical lines are plotted as a visual separation between the 5 chromosomes and the chromosome number is indicated on the x-axis below each segment. The dotted horizontal blue line marks the $-\log _{10}$ *p*-value score of 3. Red dots on the x-axis are placed on marker positions for which NCR estimated non-zero coefficients. The color transparency indicates the magnitude of the regularized estimated coefficient. The correspondence between CIM and NCR can be seen by noticing that red dots on the x-axis are in most areas where the $-\log _{10}$ *p*-value score has high values

**Fig. 4:**
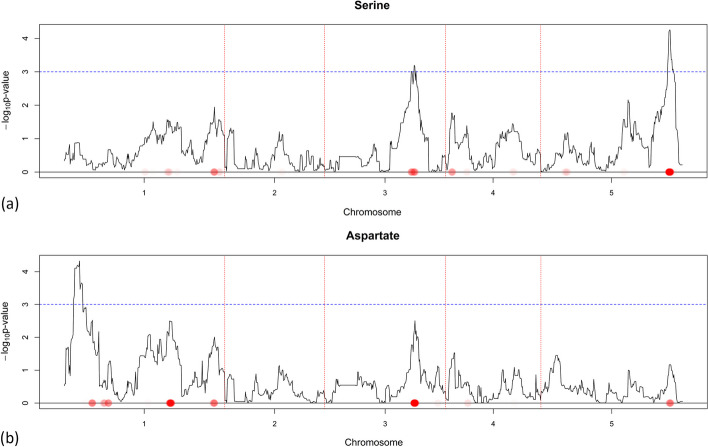
QTL detection for Serine (**a**) and Aspartate (**b**) using CIM and NCR when the guiding dataset is SNP data. They only share an edge in ${\varvec{W}}({\varvec{Y}}^{M})$, but do not share an edge in ${\varvec{W}}(\hat{{\varvec{Y}}}^{M}({\varvec{X}}^S))$, which can indicate that the unique QTLs (for a pair of metabolites) can neutralize correlation induced by common QTLs. In this case unique QTLs are responsible for a bigger part of the metabolic variation. The $-\log _{10}$ *p*-value score for every marker is plotted when CIM is used. The red dotted vertical lines are plotted as a visual separation between the 5 chromosomes and the chromosome number is indicated on the x-axis below each segment. The dotted horizontal blue line marks the $-\log _{10}$ *p*-value score of 3. Red dots on the x-axis are placed on marker positions for which NCR estimated non-zero coefficients. The color transparency indicates the magnitude of the regularized estimated coefficient. The correspondence between CIM and NCR can be seen by noticing that red dots on the x-axis are in most areas where the $-\log _{10}$ *p*-value score has high values

**Fig. 5:**
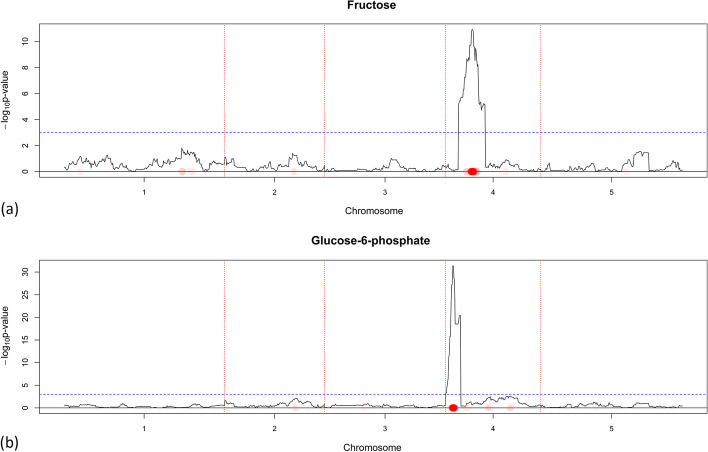
Fructose (**a**) and Glucose-6-phosphate (**b**) do not have similar QTLs and therefore do not share an edge in ${\varvec{W}}(\hat{{\varvec{Y}}}^{M}({\varvec{X}}^S))$. The $-\log _{10}$ *p*-value score for every marker is plotted when CIM is used. The red dotted vertical lines are plotted as a visual separation between the 5 chromosomes and the chromosome number is indicated on the x-axis below each segment. The dotted horizontal blue line marks the $-\log _{10}$ *p*-value score of 3. Red dots on the x-axis are placed on marker positions for which NCR estimated non-zero coefficients. The color transparency indicates the magnitude of the regularized estimated coefficient. The correspondence between CIM and NCR can be seen by noticing that red dots on the x-axis are in most areas where the $-\log _{10}$ *p*-value score has high values

**Fig. 6:**
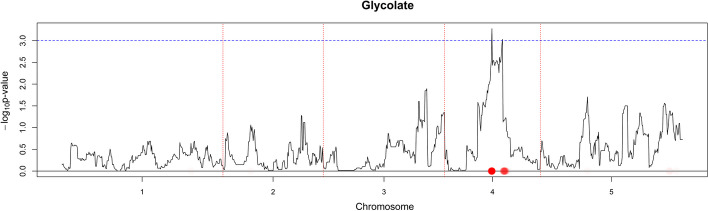
Glycolate’s QTL profile using CIM and NCR. The $-\log _{10}$ *p*-value score for every marker is plotted when CIM is used. The red dotted vertical lines are plotted as a visual separation between the 5 chromosomes and the chromosome number is indicated on the x-axis below each segment. The dotted horizontal blue line marks the $-\log _{10}$ *p*-value score of 3. Red dots on the x-axis are placed on marker positions for which NCR estimated non-zero coefficients. The color transparency indicates the magnitude of the regularized estimated coefficient. The correspondence between CIM and NCR can be seen by noticing that red dots on the x-axis are in most areas where the $-\log _{10}$ *p*-value score has high values

The vector of estimated SNP coefficients can also be used to detect QTLs. Regions where we find SNPs with non-zero coefficients should be highlighted as possible QTL regions. In Figs. 3, 4, 5 and 6 we provide some results of the correspondence between *Composite Interval Mapping* (CIM; *qtl* R-package) [27] and QTL detection using NCR while in the Additional file 1 we present all metabolites. By closely inspecting Figs. 3, 4, 5 and 6, it is evident that by using NCR we find positions on the chromosome with high CIM test statistic and subsequently possible QTL regions.

The GABA/Maltose pair (Fig. 3) clearly had similar QTLs and thus share an edge. The Serine/Aspartate (Fig. 4) and the GABA/Glucose-6-phosphate (Figs. 3, 5) pairs had an overlap in their QTL profiles but share no edge, which might indicate that the non common potentially identified QTLs are responsible for a big part of the metabolic variation. Finally, the Fructose/Glucose-6-phosphate (Fig. 5) and GABA-Glycolate (Figs. 3, 6) pairs do not have overlap in QTLs justifying why there are no edges in $\hat{{\varvec{W}}}(\hat{{\varvec{Y}}}^{M}({\varvec{X}}^S))$.

Summarizing, when two metabolites are connected, we usually observe a similarity in QTLs. On the other hand, when metabolites do not share an edge, this is generally due to dissimilar QTLs. Still, there can be situations where metabolites with similar QTLs are not connected, because of either measurement noise, or because non-overlapping QTLs account for a big part of the metabolic variation.

#### Multigraph representation

**Fig. 7:**
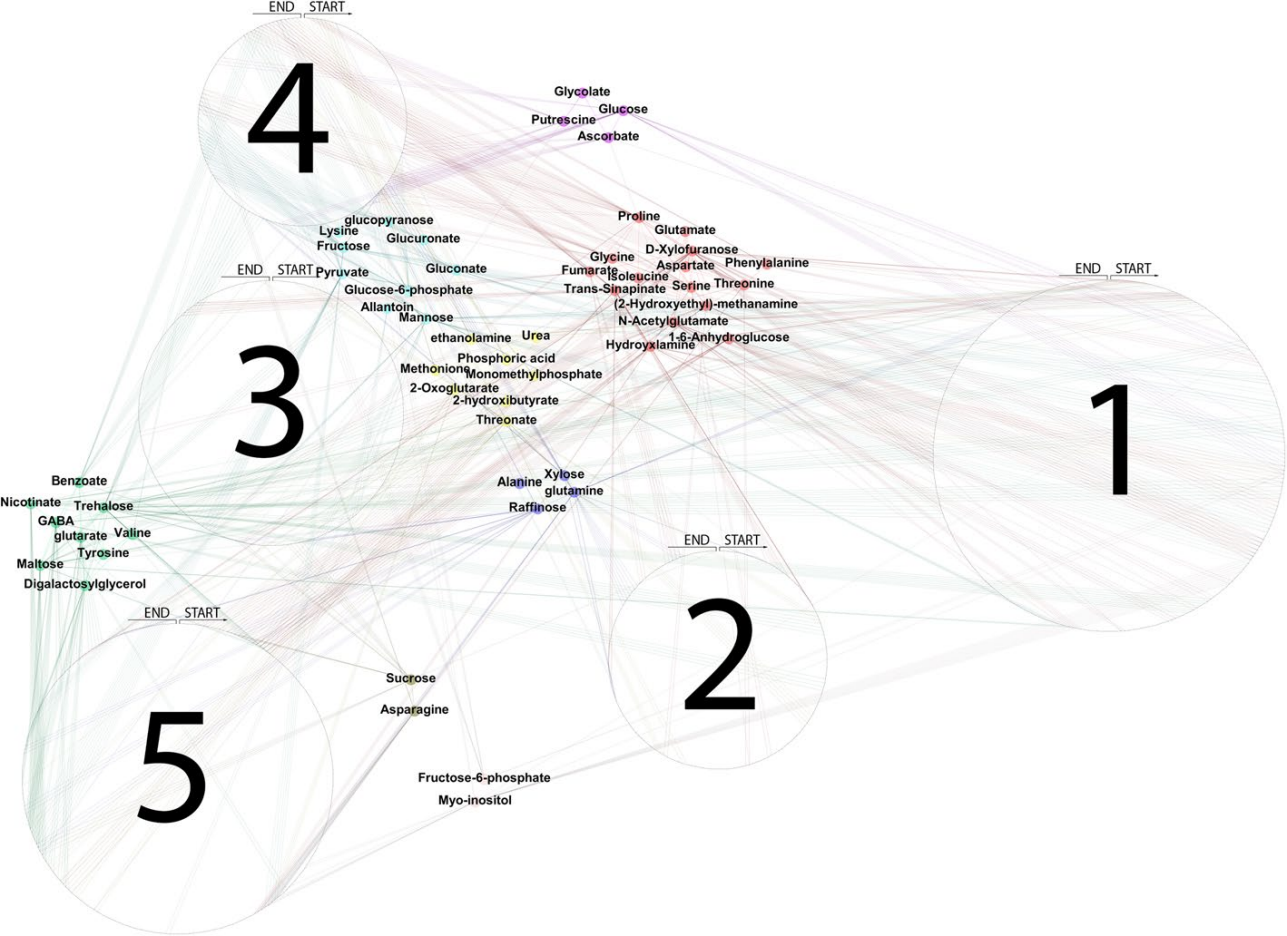
Combined network of metabolites and SNPs. ${\varvec{W}}(\hat{{\varvec{Y}}}^{M}({\varvec{X}}^S))$ is visualized together with the five chromosomes which have been folded to be represented by five circular structures. The start and end of each chromosome is at the topmost part (moving clockwise for proceeding from start to end). Non-zero SNP coefficients for every individual model have been visualized as edges connecting metabolites and SNPs. Metabolites have been colored to ease visual inspection

An informative representation can be obtained by visualizing a network that combines all data used here. In Fig. 7, $\hat{{\varvec{W}}}(\hat{{\varvec{Y}}}^{M}({\varvec{X}}^S))$ and all markers have been visualized; the nodes have been colored so that visual inspection is easier. The 5 chromosomes have been depicted as circular with the start and end being at the topmost point (one moves clockwise from start to end). Edges between a metabolite *p* and SNPs denote the non-zero estimated coefficients $\hat{\varvec{\beta }}^{S}_{p}$.

By looking at Figs. 2 and 7 we identify three interesting metabolite groups. The first consists of: *Nicotinate, GABA, Benzoate, Glutarate, Tyrosine, Digalactosyglycerol, Valine, Trehalose*, and *Maltose*. In Fig. 2, the edges between the metabolites are green meaning that they are grouped together after using SNP information. Some of them are connected to the biosynthesis of alkaloids derived from shikimate pathway. In Fig. 7 they have been represented as the dark green colored cluster sharing many edges with chromosome 5.

The second interesting metabolite group consists of: *Allantoin, Gluconate, Fructose, Glucuronate, Mannose, Glucopyranose*, and *Glucose-6-phosphate* and has been colored as ciel blue in Fig. 7. These metabolites did not share any connections with any metabolites in $\hat{{\varvec{W}}}({\varvec{Y}}^{M})$ but formed a cluster when SNP information was used ($\hat{{\varvec{W}}}(\hat{{\varvec{Y}}}^{M}({\varvec{X}}^S))$). Those metabolites are involved in sucrose metabolism, glycolysis and are either sugars or closely related to sugars.

Finally, the most interesting metabolite group contains: *Phenylalanine, Proline, Isoleucine, Aspartate, N-Acetylglutamate, Glutamate, (2-Hydroxyethyl)-methanamine, Glycine, Serine,* and *Threonine*. This metabolite group is the one with most grey edges in Fig. 2, showing that it retained most of its edges when we include non-genetic SNP variation. All metabolites in this group are contained in the biosynthesis of amino-acids. They have been colored red in Fig. 7 and show strong association with chromosomes 1, 4, and 5.

### From genes to metabolites

In the first example we used SNP data, where the network structure was simple. Nevertheless, in many applications, e.g. gene expression data, the underlying network structure is far more complicated than a linear distance-based network and not known *a priori*. In this second example we recover metabolite networks by utilizing gene information.

#### Step 1: Reconstruction of the gene expression network

In order to reconstruct the gene expression network, we use GL coupled with StARS on ${\varvec{X}}^G$. The selected regularization parameter based on subsampling was equal to 0.82, resulting in 3347 edges (sparsity of 0.00078). Our strict selection was based on the intention to minimize edges between metabolites due to false positives in gene expression data. The sparse gene expression intensity matrix $\hat{{\varvec{W}}}({\varvec{X}}^G)$ contains the absolute values of the resulting inverse covariance matrix.

#### Step 2: Estimating the metabolite part related to transcriptional variation

We use expression (2) with ${\varvec{Y}}^M$ as response and the gene expression data ${\varvec{X}}^G$ as predictors, having an estimated network structure $\hat{{\varvec{W}}}({\varvec{X}}^G)$. The *p*-th metabolite is regressed on all genes. The vector of estimated coefficients $\hat{\varvec{\beta }}^G_{p}$ related to the *Q*1 genes is used for recovering the fitted metabolite values related to transcriptional variation ($\hat{{\varvec{Y}}}^{M}({\varvec{X}}^G)$) as:

6 $$\begin{aligned} \hat{{\varvec{y}}}_{p}^{M}({\varvec{X}}^G)={\varvec{X}}^G\hat{\varvec{\beta }}^G_{p} \end{aligned}$$

#### Step 3: Metabolite networks related to gene variation

**Fig. 8:**
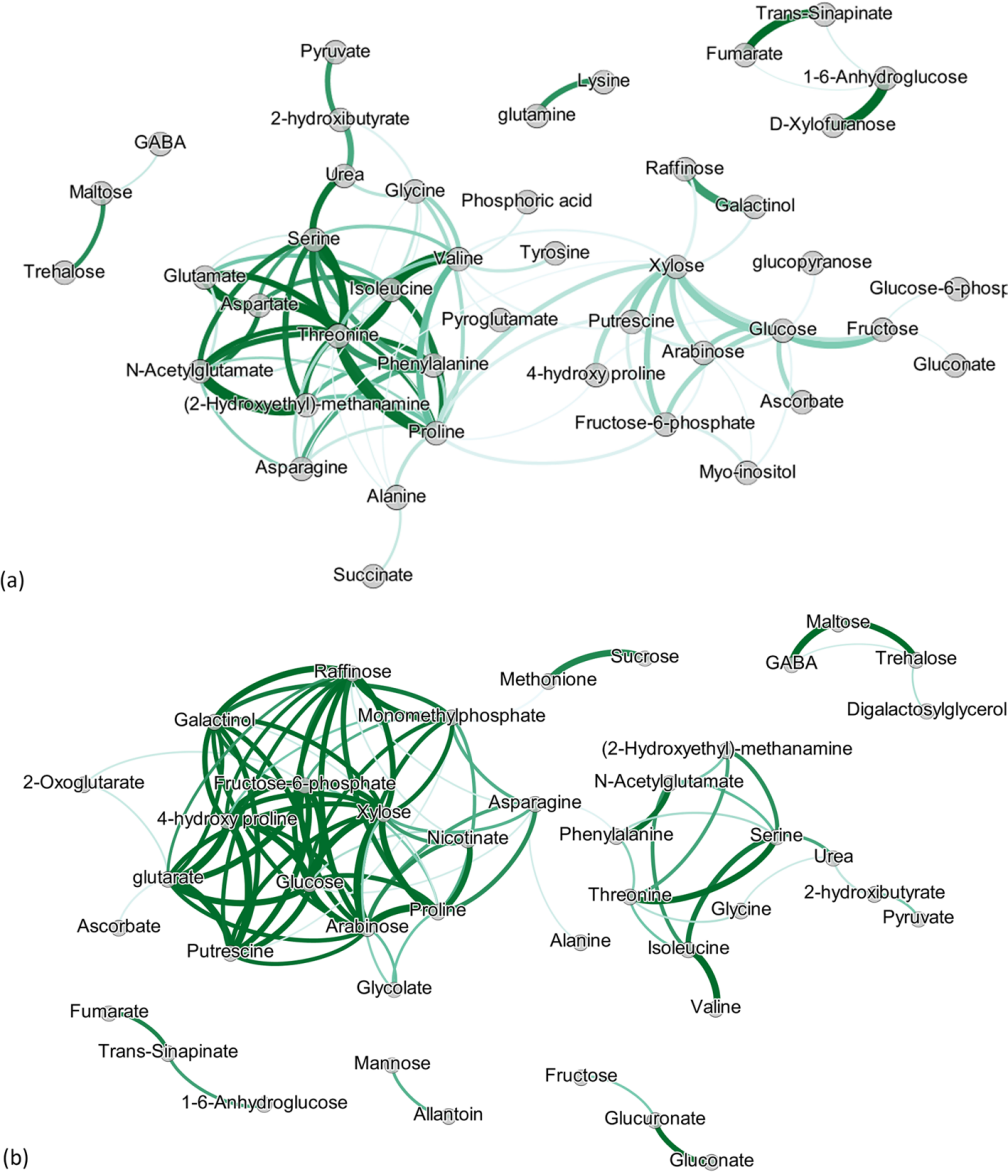
Estimated metabolite networks when: (**a**) using the original metabolite data (${\varvec{W}}({\varvec{Y}}^{M})$), and (**b**) using information on SNPs and their network structure (${\varvec{W}}(\hat{{\varvec{Y}}}^{M}(X^G))$). Edges’ width denotes the intensity of the association between two nodes, while edges’ opacity indicates the uncertainty as measured by the edges' standard deviation

To estimate metabolite networks, we use GL on the fitted metabolite values related to transcriptional variation, i.e. $\hat{{\varvec{Y}}}^{M}({\varvec{X}}^G)$. For comparing with $\hat{{\varvec{W}}}({\varvec{Y}}^{M})$, the regularization parameter $\lambda ^G$ was selected equal to 0.69, resulting in 98 edges for the metabolite network related to gene variation ($\hat{{\varvec{W}}}(\hat{{\varvec{Y}}}^{M}({\varvec{X}}^G))$). The resulting network has been visualized in Fig. 8 together with $\hat{{\varvec{W}}}({\varvec{Y}}^{M})$. The edges’ width in both figures, denotes the intensity of the connection between the metabolites. The opacity represents the uncertainty for the edge intensity and has been computed based on resampling as in example 1. In Fig. 8, we see that the uncertainty of the edges is lower in $\hat{{\varvec{W}}}(\hat{{\varvec{Y}}}^{M}({\varvec{X}}^G))$ compared to $\hat{{\varvec{W}}}({\varvec{Y}}^{M})$. By examining $\hat{{\varvec{W}}}(\hat{{\varvec{Y}}}^{M}({\varvec{X}}^G))$, we see that the top connected metabolites are *Arabinose, Xylose, Glucose, Raffinose, Fructose-6-phosphate*, and *Monomethylphosphate* with 13, 13, 12, 12, 11, and 11 edges, respectively.

Metabolites are mainly connected because they are associated to similar (or connected) genes. On the other hand, metabolites that are not connected are usually associated with different sets of genes.

#### Network of differences between ${\varvec{W}}(\hat{{\varvec{Y}}}^{M}({\varvec{X}}^G))$ and ${\hat{{\varvec{W}}}}({\varvec{Y}}^{M})$

**Fig. 9:**
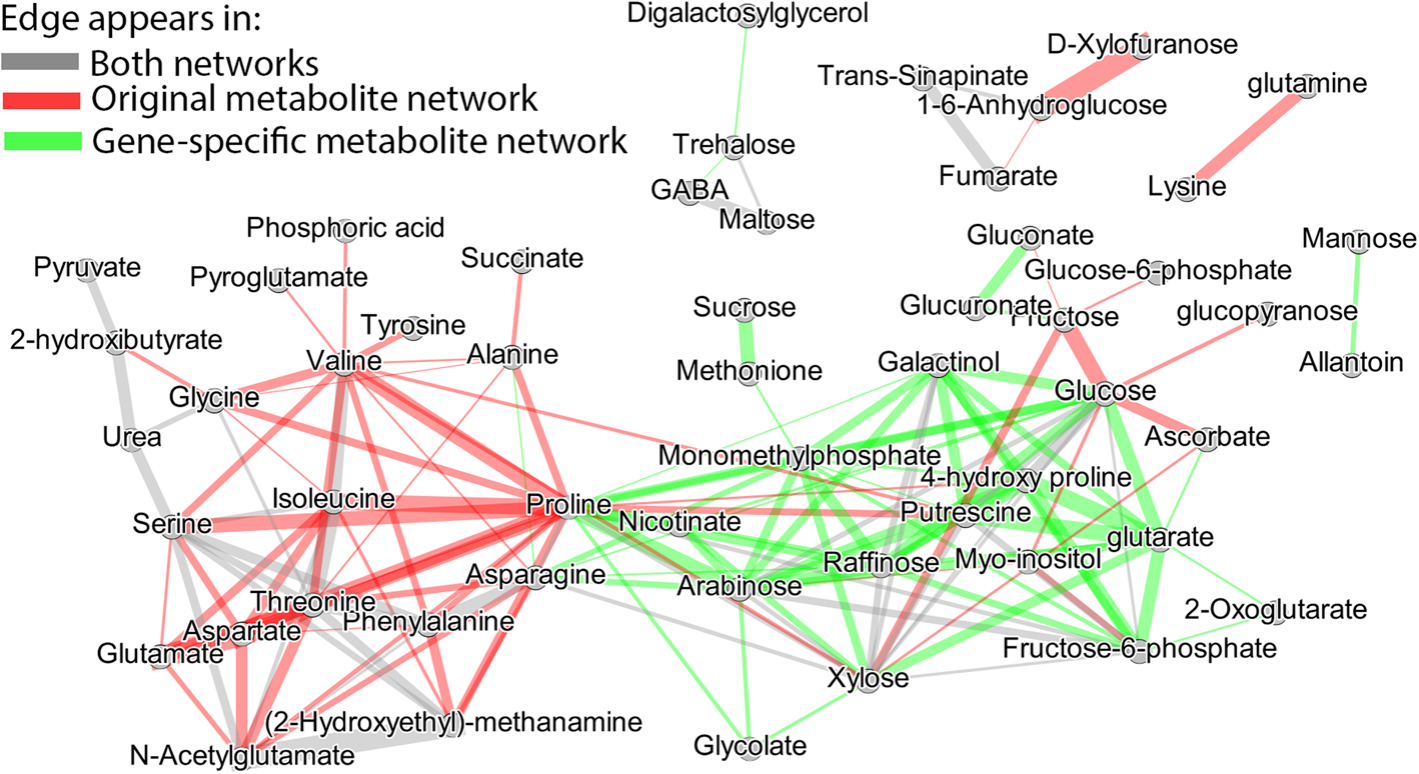
Difference between network based on the original metabolite values (${\varvec{W}}({\varvec{Y}}^{M})$) and network reconstructed when gene expression is used (${\varvec{W}}(\hat{{\varvec{Y}}}^{M}(X^G))$). Green edges denote the unique edges that appear in ${\varvec{W}}(\hat{{\varvec{Y}}}^{M}(X^G))$. Red denote the unique edges appearing in ${\varvec{W}}({\varvec{Y}}^{M})$. Grey edges are the common edges between ${\varvec{W}}(\hat{{\varvec{Y}}}^{M}(X^G))$ and ${\varvec{W}}({\varvec{Y}}^{M})$. The width of the edges denotes the difference between the connections’ intensity of ${\varvec{W}}(\hat{{\varvec{Y}}}^{M}(X^G))$ and ${\varvec{W}}({\varvec{Y}}^{M})$

To highlight the major differences between the networks we visualize their differences in Fig. 9. Edges are colored with green if they only appear in $\hat{{\varvec{W}}}(\hat{{\varvec{Y}}}^{M}({\varvec{X}}^G))$, red if they only appear in $\hat{{\varvec{W}}}({\varvec{Y}}^{M})$, and grey if they appear in both. By examining the differences between the networks, we make the following observations.

The metabolites losing most of their edges are *Proline* (13) and *Valine* (12) showing that their similarity with other metabolites is not due to the transcriptional part of variation. Those metabolites lost many edges in the SNP example as well, showing that their correlation with other metabolites is driven by other sources of variation. Other metabolites losing multiple edges when we use only gene information are: *Aspartate* (7), *Threonine*(7), and *Glutamate* (6).

On the other hand, the metabolites gaining edges when gene variation is used are *Monomethylphosphate* (11), *Arabinose* (10), *Glutarate* (10), *Raffinose* (10), and *Galactinol* (8). Finally, the metabolites keeping most of their edges are: *Xylose* (9), *Serine* (6), *Fructose-6-phosphate* (5), *Glucose* (5), and *Threonine* (5).

Another finding standing out when looking at Fig. 9 is the two metabolite clusters. One consists of the following amino-acids: *Proline*, *Phenylalanine*, *t Threonine*, *Isoleucine*, *Valine*, *Glycine* and *Serine*. Lastly, the cluster containing many green edges is composed of several metabolites that are related to abiotic stress responses in plants like those related to the Raffinose family of oligosaccharides [22].

## Discussion

In this work, we studied whether estimating the network structure of a particular omics level can be supported by using information coming from the network organization of another omics level. We proposed a three-step approach (Sect. 2.5) based on regularized regression that was demonstrated in two applications. Using this approach, in both applications the recovered networks contained edges with lower uncertainty compared to the original data.

For addressing missingness within guiding and target datasets, an expectation-maximization (EM) algorithm adapted for penalized network estimation offers a theoretical solution, but may escalate computational complexity. Alternatively, matrix completion techniques provide a pragmatic preprocessing step to impute missing data (e.g., [18]), thereby preparing the dataset for our network-based approach.

A natural extension of our three-step method is by using more than two datasets, e.g. SNPs, genes, and metabolites. To estimate such networks, we work sequentially from one omics source to the next. We start from SNP data and their linear structure and work our way to estimate gene expression data subject to SNP variation. Then we use the fitted gene expression values and their estimated network organization to estimate metabolite networks. Even though the rationale of such application is intuitive (propagate information from one omics level to the next), the interpretation is challenging.

By taking a step forward, since metabolites determine many quality traits (nutritional value, drought tolerance, etc) [25] and are closely related to the phenotypes [3], we could also study phenotypic associations using network analysis. By using our three-step approach for modeling phenotypic associations, we would be able to identify metabolites, genes, and DNA regions responsible for these traits. Using this approach, in plant genetics, plant breeders and physiologists can improve adaptation to environmental stress, food quality, and crop yield [20].

Finally, an interesting point of discussion is the choice of NCR in step 2 over other candidate methods, e.g., the LASSO or elastic net. In [15], these three methods have been compared in different scenarios with respect to sensitivity (true positives), specificity (true negatives) and prediction mean squared error (PMSE). The NCR procedure resulted in better PMSE making it a principal candidate for our multi-step approach. Another alternative candidate method to relate the guiding and the target datasets would be to use L2 regularization instead of L1 in (2) making it a Ridge-NCR procedure. The solution of the Ridge-NCR problem with application in genomic prediction may be more interesting, as the L1 regularization tends to drop collinear variables from the model that can potentially carry relevant information. We have presented the results of a Ridge-NCR analysis elsewhere [2]. Lastly, a hybrid between L1 and L2 penalties, aka elastic net-NCR can also be considered. Similar to LASSO, this alternative can produce reduced models by estimating zero-valued coefficients. In addition, not all collinear variables are eliminated, potentially retaining relevant information (similar to Ridge).

### Supplementary Information

**Additional file 1**. QTL concordance.

## Data Availability

The Arabidopsis thaliana data are available upon resonable request from the Authors of Joosen et al. [10]. R-code can be found through the following publicly available GitHub repository: https://github.com/bartzis-georgios/guided-network-multi-omic.
