# Supplementary material for: A guided network estimation approach using multi-omic information
Source: BMC Bioinformatics. 2024 May 30;25:202. doi: 10.1186/s12859-024-05778-7 (PMC11137963; doi:10.1186/s12859-024-05778-7)
Supplement: Supplementary file 1 — Additional file 1. QTL concordance. [file 12859_2024_5778_MOESM1_ESM.pdf]

A guided network estimation approach using multi-omic information
SUPPLEMENTARY MATERIAL

Georgios Bartzis, Carel F.W. Peeters, Wilco Ligterink, Fred A.v. Eeuwijk

1 List of SNPs associated to each metabolite:

- **(2-Hydroxyethyl)-methanamine**: RSM_5_24.75, RSM_5_24.85, RSM_5_24.65, RSM_1_28.65, RSM_1_20.95, RSM_1_21.05, RSM_5_24.95, RSM_1_20.85, RSM_4_18.25, RSM_1_28.55, RSM_1_21.15, RSM_4_18.35, RSM_1_28.75, RSM_1_20.75 RSM_3_18.15, RSM_3_18.05, RSM_4_18.15, RSM_4_18.45, RSM_1_21.25, RSM_1_28.45, RSM_4_14.45, RSM_4_14.35, RSM_4_14.55, RSM_4_14.25, RSM_2_9.75, RSM_5_25.05, RSM_4_14.65, RSM_1_28.85, RSM_2_9.85, RSM_4_1.05, RSM_4_1.15, RSM_4_14.15, RSM_5_24.55, RSM_3_18.25, RSM_1_20.65, RSM_3_17.75, RSM_3_17.65, RSM_4_14.75, RSM_3_17.85

- **1-6-Anhydroglucose**: RSM_2_1.15, RSM_5_24.95, RSM_2_1.05, RSM_5_24.85, RSM_5_25.05, RSM_5_9.35, RSM_1_6.75, RSM_1_6.65, RSM_5_24.75, RSM_5_26.25, RSM_5_9.05, RSM_5_25.15, RSM_5_9.25, RSM_2_0.95, RSM_2_1.35, RSM_5_9.15, RSM_5_9.45, RSM_5_26.35, RSM_2_19.55, RSM_1_6.05, RSM_1_6.85, RSM_1_6.55, RSM_2_19.65, RSM_2_2.15, RSM_1_7.05, RSM_5_24.65, RSM_1_5.95, RSM_2_2.05, RSM_1_6.95, RSM_2_19.45, RSM_1_6.15, RSM_4_0.85, RSM_2_0.85, RSM_5_26.15, RSM_1_5.85, RSM_5_26.05, RSM_1_7.15, RSM_5_26.45, RSM_1_6.45, RSM_5_9.75, RSM_4_0.75, RSM_1_7.25, RSM_5_25.25, RSM_4_0.95, RSM_5_25.95, RSM_1_6.25, RSM_2_2.25, RSM_5_25.35, RSM_1_6.35, RSM_1_5.75, RSM_1_7.35, RSM_5_25.85, RSM_2_1.45, RSM_5_26.55, RSM_2_18.45, RSM_4_0.65, RSM_1_22.25, RSM_5_25.75, RSM_2_19.35, RSM_1_22.15, RSM_1_22.35, RSM_1_22.45, RSM_5_9.55

- **2-hydroxibutyrate**: RSM_5_25.45, RSM_5_25.55, RSM_5_25.35, RSM_3_23.25, RSM_3_23.15, RSM_3_8.75, RSM_3_8.85, RSM_1_3.45, RSM_1_3.55, RSM_3_23.35, RSM_5_6.25, RSM_1_3.35, RSM_2_1.05, RSM_5_1.45, RSM_3_8.15, RSM_2_1.15, RSM_5_2.15, RSM_2_0.95, RSM_5_2.05, RSM_3_23.45

- **GABA**: RSM_5_7.35, RSM_5_7.45, RSM_4_0.75, RSM_5_7.55, RSM_4_0.85, RSM_4_0.65, RSM_5_7.65, RSM_5_7.25, RSM_5_7.75, RSM_4_8.95, RSM_4_8.85, RSM_4_0.95, RSM_4_9.05, RSM_5_7.85, RSM_4_1.05, RSM_1_11.15, RSM_4_0.55, RSM_4_1.15, RSM_1_11.05, RSM_4_9.15, RSM_5_6.15, RSM_4_1.25, RSM_5_7.15, RSM_5_6.05, RSM_5_9.05, RSM_1_24.35, RSM_4_1.35, RSM_5_3.05, RSM_5_0.35, RSM_4_1.45, RSM_4_9.25, RSM_1_11.25, RSM_5_9.15, RSM_5_3.15, RSM_1_24.25, RSM_4_8.75, RSM_1_11.35, RSM_4_8.25, RSM_5_17.75, RSM_2_14.75, RSM_5_17.85, RSM_2_14.85, RSM_1_24.15, RSM_1_24.45, RSM_5_6.25, RSM_2_14.65, RSM_2_14.95, RSM_1_10.75, RSM_5_3.25, RSM_1_24.05, RSM_5_3.45, RSM_1_27.95

- **4-hydroxy-proline**: RSM_3_10.55, RSM_3_10.65, RSM_3_10.35, RSM_4_18.05, RSM_4_18.15, RSM_4_18.25, RSM_4_17.95, RSM_4_5.15, RSM_3_10.75, RSM_4_5.25, RSM_3_10.25, RSM_4_4.85, RSM_4_2.15, RSM_5_23.15, RSM_4_17.85, RSM_4_2.05, RSM_4_4.25, RSM_4_2.25, RSM_4_4.15, RSM_4_4.05, RSM_4_1.95

- **Alanine**: RSM_3_15.65, RSM_3_15.55, RSM_3_15.45, RSM_3_15.75, RSM_3_15.25, RSM_1_28.45, RSM_3_13.05, RSM_3_14.05, RSM_1_28.35, RSM_1_28.55, RSM_3_12.55, RSM_3_14.25, RSM_1_22.25, RSM_1_22.15, RSM_1_28.25, RSM_3_12.45, RSM_1_28.65, RSM_3_14.35, RSM_3_15.85, RSM_3_11.95, RSM_3_14.75, RSM_1_17.45, RSM_1_17.35, RSM_1_18.25, RSM_3_16.35, RSM_5_6.05, RSM_1_18.35, RSM_1_18.15, RSM_3_15.95, RSM_1_17.55

- **2-Oxoglutarate**: RSM_5_25.75, RSM_5_10.05, RSM_5_25.65, RSM_5_25.35, RSM_5_25.55, RSM_1_22.95, RSM_5_25.45, RSM_5_7.25, RSM_5_9.35, RSM_3_23.45, RSM_5_10.25, RSM_1_23.05, RSM_4_12.75, RSM_4_12.65, RSM_3_23.35, RSM_1_23.15, RSM_3_8.85, RSM_5_9.45, RSM_5_7.35, RSM_5_25.25, RSM_1_7.85, RSM_1_7.95, RSM_1_7.75, RSM_3_23.25, RSM_5_17.35, RSM_4_12.15, RSM_5_17.45, RSM_5_17.25

- **Allantoin**: RSM_4_7.45, RSM_1_24.15, RSM_4_7.65, RSM_4_7.35, RSM_1_24.05, RSM_4_7.55, RSM_1_24.45, RSM_4_2.65, RSM_1_24.25, RSM_1_24.35, RSM_3_15.25, RSM_3_15.45, RSM_4_2.75, RSM_1_23.95, RSM_1_24.55, RSM_5_4.35, RSM_5_4.45, RSM_4_7.75, RSM_4_2.55, RSM_1_19.25, RSM_5_4.25, RSM_5_4.55, RSM_1_19.35, RSM_1_19.15, RSM_5_4.65, RSM_5_4.15, RSM_1_22.75, RSM_5_4.75, RSM_3_15.15, RSM_4_7.85, RSM_1_22.85, RSM_1_29.35, RSM_1_23.85, RSM_1_22.65, RSM_3_15.55, RSM_1_22.15, RSM_1_22.25, RSM_1_23.25, RSM_1_23.35, RSM_1_23.65, RSM_1_29.15, RSM_1_19.45, RSM_1_29.25, RSM_5_5.05, RSM_1_23.75, RSM_1_22.05

- **Arabinose**: RSM_1_25.45, RSM_5_16.95, RSM_3_11.15, RSM_4_10.25, RSM_3_11.85, RSM_5_17.05, RSM_3_11.95, RSM_3_12.45, RSM_1_25.35, RSM_3_11.25, RSM_1_25.55, RSM_1_24.75, RSM_1_24.65, RSM_5_16.85, RSM_5_16.75, RSM_3_12.55, RSM_5_16.65, RSM_4_10.35, RSM_3_13.05, RSM_1_24.85, RSM_1_24.55, RSM_3_14.05, RSM_1_22.95, RSM_3_11.55, RSM_4_10.15, RSM_3_14.25, RSM_3_11.75

- **Ascorbate**: RSM_4_12.65, RSM_4_12.55, RSM_4_12.75, RSM_4_12.85, RSM_4_13.95, RSM_5_7.75, RSM_5_7.85, RSM_4_12.45, RSM_4_0.55, RSM_4_13.85, RSM_3_10.85, RSM_4_12.95, RSM_5_8.75, RSM_5_8.85, RSM_2_18.45, RSM_4_13.75, RSM_3_10.95, RSM_5_8.95, RSM_3_11.05, RSM_3_11.15, RSM_4_0.45, RSM_1_26.45, RSM_4_13.05

- **Asparagine** , RSM_3_9.55, RSM_5_25.35, RSM_5_25.25, RSM_3_9.45, RSM_5_25.15, RSM_5_25.45, RSM_5_6.05, RSM_5_6.75, RSM_5_6.65, RSM_4_5.45, RSM_3_9.65, RSM_5_25.05, RSM_5_6.15, RSM_5_5.95, RSM_4_5.55, RSM_4_5.35, RSM_5_25.55, RSM_5_17.25, RSM_5_17.15, RSM_5_17.35, RSM_5_6.55, RSM_5_17.05, RSM_5_17.45, RSM_5_25.65, RSM_3_22.25, RSM_5_16.95, RSM_3_22.35, RSM_5_5.85, RSM_5_25.75, RSM_3_18.55, RSM_5_2.45, RSM_5_6.85, RSM_1_29.45, RSM_5_17.55, RSM_5_6.25, RSM_3_9.75, RSM_5_2.05, RSM_5_2.15, RSM_3_18.65, RSM_5_6.45, RSM_5_2.55, RSM_3_22.15

- **Aspartate**: RSM_3_18.15, RSM_3_18.25, RSM_1_20.95, RSM_1_21.05, RSM_1_21.15, RSM_3_18.05, RSM_1_4.85, RSM_1_20.85, RSM_3_18.35, RSM_1_28.65, RSM_1_28.75, RSM_1_7.55, RSM_1_7.65, RSM_5_24.85, RSM_5_24.75, RSM_1_4.75, RSM_1_21.25,

RSM_1_6.85, RSM_3_18.45, RSM_5_24.95, RSM_1_28.55, RSM_1_6.95, RSM_5_24.65, RSM_1_4.95, RSM_1_7.05, RSM_4_5.85, RSM_4_5.95, RSM_1_6.75, RSM_1_28.85, RSM_3_22.15, RSM_1_28.45, RSM_1_5.05, RSM_3_22.25, RSM_4_14.05, RSM_5_25.05, RSM_1_3.85, RSM_1_17.15, RSM_1_5.95, RSM_1_17.05, RSM_1_6.65

- **Benzoate**: RSM_5_6.85, RSM_4_1.15, RSM_5_6.75, RSM_4_1.25, RSM_5_6.65, RSM_2_8.35, RSM_4_1.35, RSM_4_9.25, RSM_5_6.95, RSM_4_1.05, RSM_4_9.15, RSM_5_6.55, RSM_2_8.25, RSM_2_2.05, RSM_2_2.15, RSM_5_9.05, RSM_2_8.45, RSM_4_9.35, RSM_5_6.45, RSM_2_18.05, RSM_5_9.15, RSM_5_5.65, RSM_5_5.55, RSM_5_5.15, RSM_2_17.95, RSM_2_18.15, RSM_5_1.55, RSM_4_1.45, RSM_2_1.95, RSM_5_0.95, RSM_2_16.05, RSM_2_15.55, RSM_2_18.25, RSM_2_17.85, RSM_5_0.85, RSM_2_2.25, RSM_5_5.05, RSM_5_7.05, RSM_5_6.35, RSM_2_15.45, RSM_5_4.65, RSM_4_9.45, RSM_3_8.15, RSM_5_4.55, RSM_5_9.25, RSM_5_9.35, RSM_4_9.05, RSM_2_15.95, RSM_2_16.15, RSM_5_4.75, RSM_2_5.95, RSM_5_5.25, RSM_2_10.15, RSM_2_1.85, RSM_2_10.25, RSM_2_2.35, RSM_2_10.05, RSM_5_4.45, RSM_4_10.25, RSM_4_0.95, RSM_5_1.45, RSM_3_8.25, RSM_5_1.65, RSM_5_1.05, RSM_4_8.85, RSM_5_4.85, RSM_4_10.85, RSM_2_9.85
- **Citrate**: RSM_1_24.35, RSM_1_24.45, RSM_1_24.25, RSM_1_24.55, RSM_1_24.15, RSM_4_0.75, RSM_4_0.65, RSM_2_8.95, RSM_4_0.85, RSM_2_9.05, RSM_2_8.85, RSM_1_24.05, RSM_1_30.45, RSM_4_0.95, RSM_4_0.55, RSM_1_30.35, RSM_2_9.15, RSM_1_24.65, RSM_2_8.75, RSM_1_30.25, RSM_1_23.05, RSM_3_20.75, RSM_1_11.15, RSM_3_6.35, RSM_2_0.05, RSM_1_23.95, RSM_3_20.85, RSM_3_6.25, RSM_1_22.85, RSM_1_22.75, RSM_1_22.95, RSM_1_23.15, RSM_2_0.15, RSM_3_6.15, RSM_1_22.65, RSM_4_0.45, RSM_1_18.95, RSM_3_20.65, RSM_3_7.35, RSM_1_30.15, RSM_1_11.05, RSM_1_19.05, RSM_3_2.05, RSM_3_1.95, RSM_1_22.55, RSM_3_7.45, RSM_1_4.45, RSM_1_4.55, RSM_5_4.35, RSM_3_0.95, RSM_3_6.05, RSM_2_9.25, RSM_5_4.45, RSM_5_4.25, RSM_3_2.15, RSM_1_16.35, RSM_1_16.25, RSM_3_20.95, RSM_1_11.25, RSM_3_1.85, RSM_3_1.05, RSM_1_24.75, RSM_4_5.15, RSM_1_22.45, RSM_3_2.25, RSM_4_5.25, RSM_2_8.65, RSM_3_6.45, RSM_1_30.05, RSM_1_16.45, RSM_1_16.15, RSM_1_4.35, RSM_5_4.55, RSM_1_23.25, RSM_4_0.35, RSM_3_5.95, RSM_1_4.65, RSM_3_20.55, RSM_5_4.15, RSM_3_0.85, RSM_3_7.25, RSM_5_17.65, RSM_3_17.35, RSM_1_23.85, RSM_3_0.35, RSM_4_1.05, RSM_3_0.45, RSM_2_0.25, RSM_3_17.45, RSM_4_1.55, RSM_1_22.35, RSM_3_21.05, RSM_3_17.25, RSM_3_5.85, RSM_3_0.75, RSM_5_26.45, RSM_3_1.75, RSM_1_16.65, RSM_3_17.65, RSM_3_7.85, RSM_3_7.95, RSM_3_0.55, RSM_3_5.75, RSM_5_26.35, RSM_2_7.35, RSM_3_0.65, RSM_1_23.65, RSM_5_4.65, RSM_4_4.85, RSM_5_17.75, RSM_5_17.55, RSM_4_2.35, RSM_1_18.75, RSM_1_6.75, RSM_3_7.55, RSM_3_7.75, RSM_1_29.95, RSM_3_17.55, RSM_1_11.35, RSM_3_19.95, RSM_1_6.65, RSM_3_8.05, RSM_1_24.85, RSM_3_17.75, RSM_4_0.25, RSM_3_7.65, RSM_5_15.95, RSM_2_9.35, RSM_3_5.65, RSM_2_8.55, RSM_4_2.55, RSM_2_7.45, RSM_5_15.85, RSM_3_21.65, RSM_1_15.95, RSM_3_21.55, RSM_1_23.35, RSM_3_20.45, RSM_4_2.45, RSM_5_26.55, RSM_3_1.65, RSM_3_20.05, RSM_4_5.35, RSM_5_25.95, RSM_1_23.75, RSM_4_4.25, RSM_3_17.15, RSM_3_21.45, RSM_5_17.45, RSM_5_17.85, RSM_2_7.25, RSM_5_25.85, RSM_3_7.15, RSM_1_13.25, RSM_3_2.35, RSM_4_1.85, RSM_3_1.15, RSM_1_18.65, RSM_1_13.15, RSM_1_19.15, RSM_5_25.45, RSM_1_6.85, RSM_4_1.45, RSM_4_2.25, RSM_4_2.65, RSM_1_29.45, RSM_3_21.15, RSM_2_18.55, RSM_1_5.65, RSM_1_4.75, RSM_1_10.75, RSM_1_5.55, RSM_3_6.55, RSM_1_17.95, RSM_4_6.85, RSM_5_26.65, RSM_1_11.45, RSM_1_16.75, RSM_1_18.55
- **Digalactosylglycerol**: RSM_5_5.15, RSM_5_5.25, RSM_5_5.35, RSM_5_7.05, RSM_5_5.45, RSM_5_6.95, RSM_5_5.05, RSM_5_7.15, RSM_5_8.95, RSM_5_9.05,

RSM_5_8.85, RSM_5_7.75, RSM_5_7.85, RSM_5_7.25, RSM_5_6.85, RSM_5_5.55, RSM_3_8.05, RSM_3_8.15, RSM_5_8.75, RSM_4_7.85, RSM_5_7.35, RSM_4_7.75

- **D-Xylofuranose**: RSM_5_24.85, RSM_5_24.95, RSM_5_24.75, RSM_5_25.05, RSM_1_6.75, RSM_1_6.65, RSM_3_17.65, RSM_5_24.65, RSM_4_0.85, RSM_3_17.75, RSM_4_0.75, RSM_3_17.55, RSM_5_25.15, RSM_1_6.85, RSM_2_1.35, RSM_2_1.45, RSM_2_18.95, RSM_2_19.05, RSM_2_1.15, RSM_1_6.95, RSM_3_17.85, RSM_5_19.35, RSM_5_25.35, RSM_2_19.15, RSM_4_5.95, RSM_5_25.25, RSM_4_5.85, RSM_2_2.05, RSM_2_18.85, RSM_1_7.05, RSM_1_22.05, RSM_5_19.45, RSM_1_22.15, RSM_1_6.55, RSM_5_19.15, RSM_5_19.25, RSM_2_1.55, RSM_2_2.15, RSM_1_21.95, RSM_1_29.75, RSM_1_26.15, RSM_1_29.65, RSM_2_0.15, RSM_5_25.45, RSM_5_25.55, RSM_1_21.85, RSM_5_24.55, RSM_5_25.75, RSM_1_21.05, RSM_1_20.95, RSM_5_25.65, RSM_1_26.05, RSM_1_22.25, RSM_5_19.05, RSM_4_0.95, RSM_1_28.55, RSM_1_20.85, RSM_1_21.15, RSM_1_29.85, RSM_1_26.25, RSM_3_17.45, RSM_2_0.05, RSM_3_17.95, RSM_1_28.45, RSM_1_7.25, RSM_2_0.25, RSM_5_9.05, RSM_1_29.55, RSM_2_1.95, RSM_5_8.95, RSM_5_2.65, RSM_5_2.75, RSM_1_21.75, RSM_1_20.75, RSM_1_7.35, RSM_4_0.65, RSM_2_18.45, RSM_5_26.25, RSM_5_8.85, RSM_4_6.05, RSM_1_28.65, RSM_1_21.25, RSM_4_7.55, RSM_4_7.65, RSM_1_7.15, RSM_3_18.05, RSM_2_1.65, RSM_5_2.55, RSM_5_2.85, RSM_2_19.25, RSM_2_18.55, RSM_1_29.45, RSM_2_18.75, RSM_5_9.65, RSM_5_8.75, RSM_5_17.75, RSM_5_17.85, RSM_5_9.45, RSM_1_16.75, RSM_2_1.85, RSM_2_1.75, RSM_1_27.55, RSM_5_9.35, RSM_3_9.65, RSM_1_20.65, RSM_1_6.45, RSM_1_1.45, RSM_4_6.15, RSM_4_6.55, RSM_5_9.75, RSM_4_0.35, RSM_2_0.35, RSM_1_25.95, RSM_4_7.75, RSM_4_7.45
- **Ethanolamine**: RSM_5_22.05, RSM_5_21.95, RSM_2_1.15, RSM_5_21.85, RSM_5_22.15, RSM_2_1.35, RSM_4_18.05, RSM_2_1.05, RSM_1_29.75, RSM_4_18.15, RSM_1_29.65, RSM_1_29.85, RSM_4_0.65, RSM_4_17.75, RSM_5_22.25, RSM_4_18.25, RSM_1_28.25, RSM_1_29.55, RSM_5_21.75, RSM_4_7.75, RSM_5_21.35, RSM_5_21.45, RSM_4_17.95, RSM_5_19.25, RSM_1_28.35
- **Fructose**: RSM_4_6.65, RSM_4_6.75, RSM_4_6.55, RSM_4_6.85, RSM_4_7.15, RSM_4_6.45, RSM_4_7.55, RSM_4_7.45, RSM_4_7.25, RSM_4_5.75, RSM_1_23.05, RSM_4_7.75, RSM_4_6.35, RSM_4_7.65, RSM_1_22.95, RSM_4_5.65, RSM_1_23.15, RSM_4_7.85, RSM_4_7.35, RSM_2_14.25, RSM_1_23.95, RSM_4_12.65, RSM_1_2.85, RSM_4_12.75, RSM_1_2.75, RSM_2_14.35, RSM_4_5.55, RSM_2_14.45, RSM_1_24.65, RSM_4_6.25, RSM_1_2.65, RSM_1_24.75, RSM_2_14.55, RSM_4_12.55, RSM_1_25.15, RSM_2_0.05, RSM_1_25.05, RSM_2_14.65, RSM_1_2.95, RSM_1_23.25, RSM_5_25.35, RSM_1_24.55, RSM_1_7.85, RSM_4_5.85, RSM_4_9.95, RSM_1_24.05, RSM_2_14.75, RSM_5_17.35, RSM_5_17.25, RSM_2_0.15, RSM_1_7.75, RSM_1_7.95, RSM_5_25.25, RSM_5_17.45, RSM_1_25.35, RSM_4_12.85, RSM_5_17.15, RSM_1_24.45, RSM_2_14.85, RSM_5_17.55
- **Fructose-6-phosphate**: RSM_2_9.85, RSM_2_9.75, RSM_2_9.65, RSM_4_10.35, RSM_2_9.95, RSM_5_25.75, RSM_5_25.65, RSM_5_25.55, RSM_4_10.45, RSM_3_11.55, RSM_3_11.75, RSM_4_10.25, RSM_3_15.55, RSM_3_15.45, RSM_3_11.25, RSM_3_11.85, RSM_1_22.95, RSM_3_15.25, RSM_2_10.45, RSM_4_12.55, RSM_4_12.45, RSM_2_9.55, RSM_3_15.15, RSM_1_23.05, RSM_4_12.35, RSM_4_10.55, RSM_3_15.65, RSM_5_7.95
- **Fumarate**: RSM_4_0.65, RSM_5_24.75, RSM_4_0.75, RSM_5_24.85, RSM_4_0.85, RSM_5_24.65, RSM_5_24.95, RSM_4_0.95, RSM_2_10.05, RSM_2_9.95, RSM_2_11.15, RSM_4_0.55, RSM_1_6.95, RSM_3_17.75, RSM_1_18.15, RSM_3_11.85, RSM_2_11.25,

RSM_2_10.15, RSM_1_7.05, RSM_3_17.65, RSM_2_11.35, RSM_1_18.05, RSM_2_9.85, RSM_2_11.05, RSM_1_22.25, RSM_1_22.15, RSM_1_17.95, RSM_1_6.85, RSM_1_22.35, RSM_1_18.25, RSM_2_11.45, RSM_1_22.45, RSM_2_10.95, RSM_5_25.05, RSM_2_2.25, RSM_1_22.05, RSM_2_2.15, RSM_3_11.95, RSM_5_10.05, RSM_2_1.05, RSM_3_17.85, RSM_1_24.55, RSM_2_11.55, RSM_1_23.75, RSM_1_18.35, RSM_1_23.85, RSM_1_24.65, RSM_1_28.35, RSM_3_11.05, RSM_1_22.55, RSM_1_24.45, RSM_5_24.55, RSM_2_2.05, RSM_3_10.95, RSM_2_0.95, RSM_1_28.45, RSM_3_11.75, RSM_2_10.85, RSM_1_6.05, RSM_1_21.95, RSM_1_28.55, RSM_3_11.15, RSM_1_24.75, RSM_1_28.65, RSM_5_26.25, RSM_3_2.35, RSM_1_6.75, RSM_1_29.75, RSM_3_2.45, RSM_5_25.35, RSM_4_0.45, RSM_1_5.85, RSM_3_2.05, RSM_3_1.95, RSM_1_29.65, RSM_1_29.85, RSM_1_17.85, RSM_1_28.25, RSM_2_10.25, RSM_1_5.75, RSM_1_5.95, RSM_5_25.15, RSM_3_12.45, RSM_2_10.75, RSM_2_1.15, RSM_5_25.25, RSM_1_29.25, RSM_3_2.25, RSM_1_24.25, RSM_1_24.15

- **Galactinol**: RSM_4_13.05, RSM_4_13.15, RSM_2_9.35, RSM_4_12.95, RSM_5_1.45, RSM_5_21.35, RSM_5_21.25, RSM_3_11.85, RSM_2_9.45, RSM_4_13.25, RSM_5_1.55, RSM_5_25.55, RSM_5_25.65, RSM_3_11.75, RSM_2_9.25, RSM_5_21.45, RSM_5_25.45, RSM_4_12.85, RSM_3_11.55, RSM_4_13.35, RSM_5_1.35, RSM_5_1.65, RSM_5_26.15, RSM_2_9.15, RSM_5_25.75, RSM_3_11.95, RSM_3_11.15, RSM_3_11.25, RSM_2_9.55, RSM_2_7.85, RSM_5_25.35, RSM_2_7.95, RSM_5_7.85, RSM_5_26.05, RSM_2_9.05, RSM_5_26.25, RSM_1_3.45, RSM_1_3.55, RSM_5_16.35, RSM_5_16.25, RSM_2_7.75, RSM_4_12.75, RSM_1_3.35, RSM_1_3.65, RSM_5_1.25, RSM_1_29.95, RSM_5_16.45, RSM_5_7.75, RSM_1_29.85, RSM_4_12.15, RSM_5_16.15, RSM_5_21.15, RSM_5_21.55, RSM_4_13.45, RSM_1_29.75, RSM_2_18.45, RSM_1_30.05, RSM_2_8.95, RSM_3_11.05, RSM_1_29.65, RSM_5_7.95, RSM_5_6.15, RSM_1_3.25, RSM_4_12.05, RSM_2_8.65, RSM_2_8.55, RSM_5_6.05, RSM_3_12.45, RSM_5_16.55, RSM_5_6.25, RSM_4_2.75, RSM_2_8.45, RSM_1_30.15, RSM_4_2.65, RSM_5_16.05, RSM_2_8.85, RSM_3_10.95, RSM_5_25.95, RSM_2_8.75, RSM_5_1.15, RSM_2_18.55, RSM_1_29.55, RSM_5_25.85, RSM_4_12.65, RSM_4_1.55, RSM_3_10.85, RSM_5_6.95, RSM_2_9.65, RSM_1_25.35, RSM_3_1.65, RSM_5_6.85, RSM_5_6.35, RSM_5_1.05, RSM_4_1.15, RSM_3_1.75, RSM_3_1.25, RSM_3_1.35, RSM_3_1.45, RSM_3_1.55, RSM_1_25.25, RSM_5_6.75, RSM_2_10.65, RSM_2_10.55, RSM_4_1.05, RSM_1_25.15, RSM_1_30.25, RSM_3_12.55, RSM_4_1.25, RSM_1_25.05, RSM_5_21.65, RSM_4_1.35, RSM_5_0.55, RSM_4_1.45, RSM_5_6.45, RSM_1_26.45, RSM_1_26.35

- **Gluconate**: RSM_4_6.75, RSM_4_6.85, RSM_4_6.65, RSM_4_6.55, RSM_4_7.15, RSM_1_21.65, RSM_1_21.75, RSM_1_21.55, RSM_1_21.85, RSM_4_7.25, RSM_4_6.45, RSM_1_10.65, RSM_1_21.95, RSM_1_10.45, RSM_3_23.45, RSM_1_10.75, RSM_1_21.45, RSM_3_23.35, RSM_4_7.75, RSM_1_23.85, RSM_1_23.95, RSM_3_23.25, RSM_1_22.05, RSM_1_23.75, RSM_3_7.65, RSM_4_7.85, RSM_4_17.75, RSM_4_5.65, RSM_4_7.55, RSM_4_17.85, RSM_4_7.65, RSM_4_6.35, RSM_4_17.95, RSM_1_25.35, RSM_1_24.65, RSM_1_24.75, RSM_3_7.75, RSM_1_18.15, RSM_1_11.05, RSM_3_23.15, RSM_4_5.55, RSM_1_25.45, RSM_1_25.55

- **Glucopyranose**: RSM_4_7.75, RSM_4_7.65, RSM_1_10.75, RSM_1_11.05, RSM_4_7.85, RSM_1_10.65, RSM_4_7.55, RSM_1_11.15, RSM_4_7.45, RSM_1_10.45, RSM_4_7.95, RSM_1_3.85, RSM_1_3.75, RSM_2_7.25, RSM_2_7.35, RSM_3_11.25

- **Glucose**: , RSM_4_12.55, RSM_4_12.65, RSM_4_12.25, RSM_4_12.45, RSM_4_12.35, RSM_4_12.75, RSM_4_12.15, RSM_4_12.05, RSM_1_23.25, RSM_1_23.35, RSM_1_23.15, RSM_4_10.05, RSM_4_9.95, RSM_4_12.85, RSM_3_11.55, RSM_1_23.05,

RSM_1_23.45, RSM_3_11.25, RSM_1_22.95, RSM_4_11.95, RSM_3_11.75, RSM_4_2.65, RSM_4_2.75, RSM_3_11.15, RSM_4_12.95, RSM_4_9.85, RSM_1_26.45, RSM_1_26.65, RSM_4_13.05, RSM_1_26.75, RSM_4_2.55, RSM_1_23.95, RSM_3_11.85, RSM_1_26.15, RSM_1_26.05, RSM_4_10.15

- **Glucose-6-phosphate**: RSM_4_1.35, RSM_4_1.25, RSM_4_1.45, RSM_4_1.55, RSM_4_1.15, RSM_4_1.05, RSM_4_1.85, RSM_4_5.75, RSM_2_0.05, RSM_4_9.95, RSM_4_9.85, RSM_4_13.35, RSM_4_9.75, RSM_4_13.45, RSM_4_9.65, RSM_4_13.25, RSM_4_5.65, RSM_4_13.55, RSM_2_0.15, RSM_4_9.55, RSM_4_13.15, RSM_4_1.95, RSM_3_6.65, RSM_4_13.65, RSM_2_14.75, RSM_2_14.85, RSM_2_14.65, RSM_4_9.45, RSM_5_9.55, RSM_2_14.95, RSM_2_15.05, RSM_3_6.75, RSM_2_14.55, RSM_2_18.55, RSM_4_5.85, RSM_4_9.35, RSM_2_0.25, RSM_2_16.65, RSM_2_16.75, RSM_2_14.45, RSM_3_6.85, RSM_3_23.45, RSM_5_9.65, RSM_3_23.35, RSM_4_13.75

- **Glucuronate**: RSM_4_6.75, RSM_4_6.85, RSM_4_6.65, RSM_4_7.15, RSM_4_6.55, RSM_4_7.25, RSM_4_6.45, RSM_4_6.35, RSM_4_0.65, RSM_4_6.25, RSM_2_18.35, RSM_4_0.55, RSM_5_26.35, RSM_2_18.45

- **Glutamate**: RSM_5_24.75, RSM_5_24.85, RSM_1_21.85, RSM_1_21.75, RSM_5_24.65, RSM_5_24.95, RSM_1_21.95, RSM_4_13.95, RSM_4_14.05, RSM_1_21.65, RSM_1_22.05, RSM_3_18.65, RSM_1_22.15, RSM_3_18.75, RSM_4_13.85, RSM_3_22.25, RSM_1_21.55, RSM_3_18.45, RSM_3_18.55, RSM_3_22.15, RSM_3_22.35, RSM_1_22.25, RSM_3_18.85, RSM_3_21.85, RSM_1_21.45, RSM_5_25.05, RSM_4_13.75

- **Glutamine**: RSM_3_11.25, RSM_3_11.15, RSM_1_11.05, RSM_1_3.15, RSM_3_11.55, RSM_4_12.75, RSM_4_12.65, RSM_1_11.15, RSM_1_3.05, RSM_1_3.45, RSM_5_7.85, RSM_2_7.75, RSM_5_25.65, RSM_5_25.55, RSM_5_7.75, RSM_3_11.05, RSM_1_3.55, RSM_1_3.25, RSM_5_25.45, RSM_4_12.85, RSM_1_3.35, RSM_1_7.25, RSM_2_2.85, RSM_1_7.35, RSM_1_11.25, RSM_2_2.55, RSM_1_7.05, RSM_5_25.35, RSM_2_0.95, RSM_5_5.35, RSM_5_3.95, RSM_2_2.95, RSM_2_7.85, RSM_5_5.25, RSM_5_25.75, RSM_2_2.45, RSM_1_7.15, RSM_5_7.95, RSM_2_18.55, RSM_4_12.95, RSM_5_4.05, RSM_5_5.45, RSM_1_7.45, RSM_2_3.05, RSM_1_13.15, RSM_1_13.25, RSM_5_17.25, RSM_2_18.45, RSM_5_17.35, RSM_5_26.25, RSM_3_10.95, RSM_3_11.75, RSM_5_17.15, RSM_2_2.35, RSM_2_0.85, RSM_5_17.45, RSM_3_10.65, RSM_4_12.55, RSM_2_4.35, RSM_4_17.75, RSM_1_6.95, RSM_3_10.55, RSM_3_10.15, RSM_5_17.55, RSM_5_17.05, RSM_1_8.25, RSM_5_4.15, RSM_5_4.25, RSM_2_1.05, RSM_5_4.35

- **Glutarate**: RSM_5_7.25, RSM_5_7.35, RSM_5_8.45, RSM_5_8.35, RSM_5_8.55, RSM_5_2.05, RSM_5_7.15, RSM_5_2.15, RSM_5_1.95, RSM_5_2.75, RSM_4_13.55, RSM_5_2.65, RSM_5_2.85, RSM_4_13.65, RSM_4_7.15, RSM_4_13.45, RSM_4_7.25, RSM_5_2.95, RSM_5_2.55, RSM_5_2.25, RSM_4_13.15, RSM_4_13.35, RSM_4_13.25, RSM_5_7.05, RSM_4_13.75, RSM_4_6.55, RSM_5_2.35

- **Glycerate**: RSM_3_2.65, RSM_3_2.75, RSM_3_2.55, RSM_3_2.85, RSM_5_25.55, RSM_3_2.95, RSM_5_25.45, RSM_1_18.95, RSM_3_2.45, RSM_1_19.05, RSM_5_25.65, RSM_3_3.05, RSM_3_23.05, RSM_3_9.05, RSM_3_9.15, RSM_5_25.35, RSM_1_16.95, RSM_3_3.15, RSM_3_8.95, RSM_5_25.75, RSM_3_22.95, RSM_3_2.35, RSM_1_18.75, RSM_3_9.25, RSM_3_8.85, RSM_1_19.15, RSM_1_17.05, RSM_1_16.85, RSM_1_16.75, RSM_2_11.35, RSM_2_11.45, RSM_5_25.25, RSM_4_5.75, RSM_5_25.15, RSM_3_23.15, RSM_1_16.65, RSM_1_29.65, RSM_1_29.75, RSM_3_3.25, RSM_3_8.75, RSM_1_29.55, RSM_2_11.55, RSM_2_11.25, RSM_1_17.15, RSM_5_25.05, RSM_4_7.75, RSM_1_29.85,

RSM_1_16.45, RSM_1_7.85, RSM_4_5.65, RSM_1_7.75, RSM_4_7.85, RSM_1_29.45, RSM_4_5.85, RSM_1_7.95, RSM_1_19.25, RSM_3_3.35, RSM_3_23.25, RSM_1_29.95, RSM_3_22.85, RSM_3_18.45, RSM_1_18.65, RSM_4_5.95, RSM_3_0.85, RSM_3_0.75, RSM_1_7.65, RSM_3_18.55, RSM_1_29.35, RSM_1_17.25

- **Glycine**: RSM_5_23.95, RSM_5_24.05, RSM_1_29.35, RSM_1_29.25, RSM_5_23.85, RSM_1_27.65, RSM_5_24.15, RSM_1_28.05, RSM_5_22.05, RSM_5_23.75, RSM_1_29.15, RSM_1_29.45, RSM_4_5.45, RSM_1_28.85, RSM_4_5.55, RSM_1_28.15, RSM_1_29.05, RSM_1_28.95, RSM_1_27.75, RSM_5_22.55, RSM_5_22.45, RSM_5_22.65, RSM_5_22.35, RSM_5_22.15, RSM_5_23.65, RSM_1_28.75, RSM_5_24.25, RSM_4_5.35, RSM_5_22.25, RSM_1_27.55, RSM_1_28.25, RSM_4_5.65, RSM_1_29.55, RSM_5_23.55, RSM_4_11.75, RSM_1_28.65, RSM_5_24.35, RSM_1_27.85, RSM_4_13.55, RSM_4_13.45, RSM_1_27.95, RSM_4_11.85, RSM_4_13.65, RSM_4_11.65, RSM_4_13.35, RSM_5_22.75, RSM_4_13.25, RSM_1_28.35, RSM_4_13.15, RSM_4_5.75, RSM_5_23.45, RSM_5_21.95, RSM_4_13.05

- **Glycolate**: RSM_4_10.35, RSM_4_10.25, RSM_4_12.45, RSM_4_12.35, RSM_4_12.55, RSM_4_12.65, RSM_4_12.75, RSM_4_10.45, RSM_4_12.25, RSM_4_10.15, RSM_4_13.05, RSM_5_24.65, RSM_1_24.85, RSM_5_24.75, RSM_5_8.05, RSM_5_24.85, RSM_5_25.95, RSM_4_13.15, RSM_5_25.85, RSM_4_12.85, RSM_2_7.25, RSM_2_7.35

- **Hexonate**: RSM_1_30.15, RSM_1_30.25, RSM_1_30.35, RSM_1_30.45, RSM_1_30.05, RSM_4_0.85, RSM_3_2.55, RSM_3_2.45, RSM_1_29.95, RSM_3_2.35, RSM_4_0.95, RSM_3_2.65, RSM_3_2.25, RSM_4_0.75, RSM_3_2.75, RSM_3_2.15, RSM_3_2.85, RSM_1_19.25, RSM_1_19.35, RSM_4_1.05, RSM_1_19.45, RSM_1_19.15, RSM_1_29.85, RSM_3_2.95, RSM_5_4.15, RSM_4_14.35, RSM_4_14.25, RSM_5_4.05, RSM_4_14.45, RSM_3_2.05, RSM_4_14.15, RSM_3_3.05, RSM_4_1.15, RSM_4_14.55, RSM_4_15.95, RSM_1_19.55, RSM_5_4.25, RSM_4_16.05, RSM_4_15.85, RSM_2_15.05, RSM_4_15.75, RSM_3_22.75, RSM_5_3.95, RSM_2_9.05, RSM_3_22.65, RSM_4_14.05, RSM_1_26.05, RSM_2_9.15, RSM_4_15.65, RSM_1_26.15, RSM_5_3.85, RSM_4_14.65, RSM_3_22.85, RSM_2_8.95, RSM_1_19.05, RSM_2_18.05, RSM_4_16.15, RSM_3_3.15, RSM_1_29.75, RSM_4_15.55, RSM_3_22.55, RSM_3_1.95, RSM_2_17.95, RSM_2_15.15, RSM_5_2.35, RSM_1_7.55, RSM_3_1.85, RSM_1_24.75, RSM_1_24.85, RSM_1_23.35, RSM_2_18.15, RSM_5_2.25, RSM_1_7.45, RSM_5_3.75, RSM_5_4.35, RSM_1_23.25, RSM_2_9.25, RSM_2_0.65, RSM_1_7.65, RSM_4_15.45, RSM_5_2.45, RSM_2_0.75, RSM_1_7.35, RSM_2_17.85, RSM_1_1.05, RSM_2_18.25, RSM_3_22.45, RSM_1_25.95, RSM_3_22.95, RSM_1_1.15, RSM_2_8.85, RSM_1_6.65, RSM_1_24.65, RSM_1_8.05, RSM_4_16.25, RSM_4_0.65, RSM_2_15.45, RSM_2_0.85, RSM_5_3.65, RSM_1_6.75, RSM_2_10.05, RSM_1_7.25, RSM_5_5.05, RSM_2_15.55, RSM_4_13.95, RSM_1_8.15, RSM_1_0.95, RSM_5_2.15, RSM_2_0.55, RSM_1_23.45, RSM_3_20.75, RSM_3_20.55, RSM_5_4.55, RSM_3_20.65, RSM_3_20.85, RSM_4_1.25, RSM_3_23.05, RSM_3_3.25, RSM_1_1.25, RSM_4_15.35, RSM_5_5.15, RSM_1_17.25, RSM_5_4.95, RSM_1_26.25, RSM_1_7.75, RSM_1_7.15, RSM_2_8.45, RSM_4_18.05, RSM_1_18.95, RSM_2_9.95, RSM_1_6.35, RSM_3_1.75, RSM_3_20.95, RSM_2_15.25, RSM_5_4.45, RSM_1_17.35, RSM_2_14.95, RSM_3_23.15, RSM_5_4.65, RSM_1_6.25, RSM_5_7.45, RSM_1_6.45, RSM_2_8.75, RSM_1_8.25, RSM_2_0.45, RSM_2_10.15, RSM_4_13.85, RSM_1_17.45, RSM_3_20.45, RSM_4_13.35, RSM_1_1.35, RSM_5_6.95, RSM_1_6.15, RSM_3_21.05, RSM_1_7.95, RSM_2_11.65, RSM_4_16.35, RSM_1_6.55, RSM_4_13.25, RSM_2_9.35, RSM_4_13.45, RSM_1_25.35, RSM_2_14.25, RSM_4_17.95, RSM_1_23.15, RSM_1_25.25, RSM_2_8.55, RSM_2_10.35, RSM_1_7.85, RSM_5_2.05, RSM_2_0.95, RSM_4_9.85, RSM_1_25.85, RSM_2_8.35, RSM_4_9.75, RSM_2_6.15, RSM_5_6.65, RSM_1_17.55,

RSM_2_6.05, RSM_3_23.25, RSM_1_5.35, RSM_2_10.45, RSM_1_17.65, RSM_4_9.95, RSM_2_15.35, RSM_3_3.35, RSM_1_29.65, RSM_2_11.55

- **Hydroyxlamine**: RSM_4_0.45, RSM_4_0.55, RSM_1_30.05, RSM_4_0.35, RSM_1_29.95, RSM_1_30.15, RSM_1_18.05, RSM_1_17.95, RSM_5_24.65, RSM_5_24.75, RSM_1_18.15, RSM_4_0.25, RSM_5_24.85, RSM_5_24.55, RSM_1_29.85, RSM_1_30.25, RSM_5_24.05, RSM_1_22.75, RSM_5_24.95, RSM_5_23.95, RSM_1_22.85, RSM_1_28.65, RSM_4_1.55, RSM_1_22.65, RSM_1_19.85, RSM_1_18.25, RSM_5_24.45, RSM_1_18.65, RSM_5_24.15, RSM_1_13.05, RSM_1_22.95, RSM_1_23.05, RSM_1_19.95, RSM_4_0.65, RSM_1_23.15, RSM_1_29.45

- **Isoleucine**: RSM_5_24.85, RSM_5_24.75, RSM_5_24.95, RSM_5_24.65, RSM_1_28.85, RSM_1_19.25, RSM_1_20.95, RSM_1_28.75, RSM_1_19.15, RSM_1_19.35, RSM_5_9.15, RSM_1_20.85, RSM_1_28.95, RSM_1_21.05, RSM_5_25.45, RSM_2_0.95, RSM_2_1.05, RSM_5_25.35, RSM_3_17.85, RSM_5_9.25, RSM_3_18.85, RSM_1_28.65, RSM_3_17.75, RSM_3_18.75, RSM_1_17.25, RSM_2_0.85, RSM_5_25.05, RSM_1_17.35, RSM_1_4.65, RSM_1_21.15, RSM_3_18.45, RSM_3_18.65, RSM_5_9.05, RSM_1_27.75, RSM_5_25.55, RSM_1_29.05, RSM_4_12.95, RSM_3_17.65, RSM_4_13.05, RSM_1_17.45, RSM_1_27.65, RSM_3_18.55, RSM_3_17.95, RSM_4_12.85, RSM_1_19.05, RSM_2_7.25, RSM_1_4.55, RSM_1_21.25, RSM_2_1.15, RSM_1_3.05, RSM_1_3.15, RSM_5_25.65, RSM_4_5.75, RSM_4_13.15, RSM_2_6.95, RSM_5_25.25, RSM_1_4.45, RSM_1_28.55, RSM_2_7.15, RSM_1_2.55, RSM_4_11.65, RSM_2_0.25, RSM_1_3.65

- **Lysine**: RSM_4_11.45, RSM_4_7.25, RSM_4_7.15, RSM_4_11.35, RSM_4_11.55, RSM_5_25.75, RSM_5_25.65, RSM_5_25.55, RSM_5_25.35, RSM_3_8.15, RSM_3_9.55, RSM_5_25.45, RSM_5_25.85, RSM_3_9.65, RSM_4_7.35, RSM_5_25.25, RSM_4_12.65, RSM_4_12.75, RSM_4_11.65, RSM_4_12.35, RSM_4_12.45, RSM_3_9.45, RSM_1_7.35, RSM_1_7.25

- **Malate**: RSM_1_3.75, RSM_1_3.65, RSM_5_8.85, RSM_5_8.95, RSM_5_8.75, RSM_1_3.85, RSM_1_23.95, RSM_5_9.05, RSM_1_23.85, RSM_1_24.05, RSM_1_23.75, RSM_1_23.65, RSM_1_24.15, RSM_1_24.25, RSM_1_24.35, RSM_1_27.45, RSM_5_9.15, RSM_4_15.35, RSM_1_27.35, RSM_1_3.55, RSM_4_15.45, RSM_5_9.25, RSM_1_30.45, RSM_1_24.45, RSM_4_15.25, RSM_5_17.65, RSM_1_30.35, RSM_2_0.05, RSM_5_17.75, RSM_1_30.25, RSM_1_27.55, RSM_5_1.75, RSM_4_15.55, RSM_1_27.25, RSM_2_0.15, RSM_4_15.15, RSM_4_5.95, RSM_5_1.65, RSM_2_11.75, RSM_1_3.95, RSM_1_30.15, RSM_1_23.55, RSM_3_15.55, RSM_4_6.05, RSM_1_1.35, RSM_2_11.85, RSM_2_12.15, RSM_5_17.85, RSM_1_27.75, RSM_5_12.55, RSM_1_24.55, RSM_2_12.05, RSM_5_12.05, RSM_2_12.25, RSM_4_6.15, RSM_4_5.85, RSM_3_11.25, RSM_5_12.95, RSM_5_1.85, RSM_5_1.55, RSM_1_27.65, RSM_5_11.65, RSM_4_15.65, RSM_3_15.65, RSM_5_17.55, RSM_1_27.85, RSM_2_11.95, RSM_4_15.05, RSM_4_2.35, RSM_1_30.05, RSM_4_5.15, RSM_3_22.65, RSM_3_14.05, RSM_3_22.55, RSM_5_0.05, RSM_5_9.35, RSM_3_13.05, RSM_1_1.45, RSM_2_0.25, RSM_3_14.25, RSM_2_12.35, RSM_3_7.45, RSM_3_15.45, RSM_5_13.25, RSM_5_9.85, RSM_4_6.25, RSM_3_7.55, RSM_5_20.05, RSM_4_6.35, RSM_5_20.15, RSM_4_5.25, RSM_5_1.45, RSM_1_23.45, RSM_3_12.55, RSM_4_2.25, RSM_3_7.65, RSM_1_1.25, RSM_5_0.15, RSM_3_14.35, RSM_1_27.15, RSM_5_10.85, RSM_4_6.45, RSM_3_10.85, RSM_3_17.65, RSM_5_2.15, RSM_4_6.65, RSM_3_7.35, RSM_3_22.75, RSM_2_19.35, RSM_3_1.25, RSM_3_22.45, RSM_4_6.55, RSM_4_6.75, RSM_5_1.35, RSM_3_14.75, RSM_4_4.85, RSM_5_5.35, RSM_3_15.75, RSM_3_17.75, RSM_1_3.45, RSM_4_15.75,

RSM_2_12.45, RSM_1_6.95, RSM_3_12.45, RSM_4_2.15, RSM_1_29.95, RSM_4_0.75, RSM_3_10.95, RSM_5_9.45, RSM_5_2.25, RSM_2_19.25, RSM_3_15.25, RSM_5_2.05, RSM_1_7.05, RSM_2_11.65, RSM_5_5.45, RSM_4_11.55, RSM_4_0.85, RSM_5_13.35, RSM_3_11.15, RSM_3_14.95, RSM_5_0.25, RSM_3_11.05, RSM_1_23.35, RSM_3_17.55, RSM_4_6.85, RSM_4_14.95, RSM_5_17.45, RSM_1_23.15, RSM_5_1.25, RSM_5_9.75, RSM_3_11.55, RSM_2_17.25, RSM_4_4.25, RSM_3_10.75, RSM_4_2.05, RSM_2_19.45, RSM_3_22.85, RSM_2_15.55, RSM_3_15.15, RSM_1_27.05, RSM_2_17.35, RSM_2_9.05, RSM_2_8.95, RSM_3_11.95, RSM_1_8.85, RSM_2_15.65, RSM_1_23.05, RSM_5_2.35, RSM_1_9.05, RSM_2_12.55, RSM_5_10.65, RSM_1_23.25, RSM_5_5.25, RSM_3_7.25, RSM_5_0.45, RSM_1_8.95, RSM_5_17.95, RSM_4_0.65, RSM_5_19.95, RSM_5_20.25, RSM_1_8.75, RSM_3_1.15, RSM_4_1.45, RSM_1_27.95, RSM_1_24.85, RSM_4_15.85, RSM_1_9.15, RSM_1_4.05, RSM_2_9.15, RSM_4_11.65, RSM_4_11.45, RSM_5_19.65, RSM_1_29.55, RSM_1_0.25, RSM_5_9.65, RSM_2_8.85, RSM_5_19.75, RSM_4_1.55, RSM_3_1.35, RSM_2_15.75, RSM_2_19.15, RSM_1_0.35, RSM_5_10.05, RSM_3_11.75, RSM_5_9.55, RSM_5_0.35, RSM_4_4.15, RSM_1_29.85, RSM_1_26.95, RSM_3_9.55, RSM_2_14.05, RSM_2_17.15, RSM_5_19.55, RSM_1_9.25, RSM_4_5.75, RSM_4_1.95, RSM_1_11.15, RSM_1_3.05, RSM_2_13.95, RSM_3_9.45, RSM_4_1.35, RSM_1_22.95, RSM_5_8.65, RSM_1_19.55, RSM_1_1.15, RSM_2_8.75, RSM_5_1.15, RSM_4_5.35, RSM_1_8.65, RSM_2_19.55, RSM_1_29.65, RSM_1_0.15, RSM_2_17.45, RSM_1_25.05, RSM_2_15.45, RSM_4_4.05, RSM_1_3.15, RSM_1_0.45

- **Maltose**: RSM_5_7.75, RSM_5_7.65, RSM_5_7.85, RSM_5_7.55, RSM_5_7.45, RSM_4_1.35, RSM_5_7.35, RSM_5_7.95, RSM_4_1.25, RSM_4_1.45, RSM_4_1.15, RSM_5_7.25, RSM_4_1.55, RSM_5_8.05, RSM_4_1.05, RSM_3_18.35, RSM_5_7.15, RSM_3_18.45, RSM_3_18.25, RSM_5_8.15, RSM_4_0.95, RSM_3_21.55, RSM_3_21.45, RSM_3_18.15, RSM_5_7.05, RSM_1_13.15, RSM_1_26.55, RSM_1_13.25, RSM_3_18.55, RSM_5_24.85, RSM_5_24.75, RSM_3_18.05, RSM_1_13.05, RSM_1_26.65, RSM_3_21.65, RSM_1_26.75, RSM_3_21.35, RSM_3_15.45, RSM_1_26.45, RSM_3_15.25, RSM_4_0.85, RSM_3_15.55, RSM_5_24.95, RSM_5_8.25, RSM_3_11.85

- **Mannitol**: RSM_4_0.35, RSM_5_0.05, RSM_4_0.45, RSM_3_18.55, RSM_1_4.55, RSM_2_19.15, RSM_2_19.25, RSM_3_18.45, RSM_1_4.45, RSM_4_13.65, RSM_4_0.25, RSM_3_18.65, RSM_2_19.05, RSM_5_7.65, RSM_1_4.65, RSM_4_13.75, RSM_5_7.75, RSM_4_13.55, RSM_5_0.15, RSM_2_19.35, RSM_4_9.65, RSM_5_7.55, RSM_5_7.85, RSM_4_13.85, RSM_3_18.35, RSM_1_28.05, RSM_3_18.75, RSM_4_0.75, RSM_4_0.85, RSM_4_9.75, RSM_2_19.45, RSM_1_5.55, RSM_5_0.25, RSM_3_18.25, RSM_1_5.65, RSM_1_0.35, RSM_1_0.25, RSM_4_13.45, RSM_4_9.55, RSM_4_9.85, RSM_5_0.35, RSM_1_0.15, RSM_3_9.45, RSM_1_28.15, RSM_4_9.95, RSM_2_19.65, RSM_3_18.15, RSM_4_0.55, RSM_1_27.95, RSM_4_14.85, RSM_1_0.05, RSM_1_5.45, RSM_3_22.15, RSM_4_0.15, RSM_1_27.85, RSM_2_18.95, RSM_3_9.55, RSM_4_14.75, RSM_4_13.95, RSM_1_5.75, RSM_4_13.35, RSM_3_2.85, RSM_3_2.75, RSM_3_2.95, RSM_2_19.55, RSM_1_29.15, RSM_3_2.65, RSM_3_3.05, RSM_3_2.15, RSM_3_2.55, RSM_4_10.05, RSM_5_9.05, RSM_4_10.15, RSM_3_3.15

- **Mannose**: RSM_4_7.15, RSM_4_6.85, RSM_5_4.15, RSM_4_7.25, RSM_4_6.75, RSM_1_20.75, RSM_1_20.65, RSM_5_4.25, RSM_1_20.85, RSM_4_6.65, RSM_1_20.95, RSM_5_4.35, RSM_1_25.55, RSM_1_21.05, RSM_1_20.55, RSM_1_25.45, RSM_5_4.45, RSM_4_6.55, RSM_1_29.15, RSM_4_7.35, RSM_1_28.05, RSM_1_28.15, RSM_1_28.45, RSM_1_21.15, RSM_1_28.55, RSM_4_6.45, RSM_1_25.65, RSM_1_28.35, RSM_4_6.35, RSM_5_24.75, RSM_1_28.65, RSM_1_28.75, RSM_5_24.65, RSM_1_29.35, RSM_1_21.25, RSM_5_24.85, RSM_1_21.55, RSM_5_4.75, RSM_1_27.65, RSM_4_6.25,

RSM_5_23.75, RSM_1_21.45, RSM_1_21.65, RSM_5_23.65, RSM_3_9.65, RSM_5_14.65, RSM_5_14.55, RSM_4_6.05, RSM_5_24.55, RSM_1_29.25, RSM_5_4.55, RSM_4_7.75

- **Methonione**: RSM_4_17.95, RSM_4_17.85, RSM_5_25.25, RSM_5_25.15, RSM_5_25.05, RSM_5_24.95, RSM_4_18.05, RSM_4_17.75, RSM_5_25.35, RSM_5_9.25, RSM_4_2.45, RSM_3_10.85, RSM_5_24.85, RSM_5_9.85, RSM_3_10.75, RSM_3_11.15, RSM_3_11.25, RSM_5_24.35

- **Monomethylphosphate**: RSM_5_25.95, RSM_5_26.05, RSM_5_25.85, RSM_2_0.05, RSM_2_0.15, RSM_5_26.15, RSM_1_28.95, RSM_1_29.05, RSM_3_16.35, RSM_4_1.35, RSM_4_1.45, RSM_3_16.25, RSM_4_1.25, RSM_4_1.55, RSM_1_28.85, RSM_3_16.15

- **Myo-inositol**: RSM_2_8.75, RSM_2_9.45, RSM_2_8.85, RSM_2_9.35, RSM_2_8.65, RSM_3_15.55, RSM_2_9.55, RSM_3_15.65, RSM_3_15.45, RSM_3_15.75, RSM_1_20.55, RSM_1_20.65, RSM_2_8.55, RSM_2_8.95, RSM_3_15.25, RSM_2_9.25, RSM_3_11.25, RSM_1_20.45, RSM_1_20.15, RSM_1_7.65, RSM_5_18.55, RSM_3_11.15, RSM_1_20.05, RSM_1_7.55, RSM_1_20.25, RSM_2_0.15, RSM_5_17.95, RSM_2_0.05, RSM_1_7.75, RSM_1_19.95, RSM_1_20.75, RSM_1_19.85, RSM_1_7.35, RSM_1_26.45, RSM_1_7.45, RSM_5_18.65, RSM_1_7.85, RSM_1_7.25, RSM_4_12.55, RSM_1_22.95, RSM_1_20.35, RSM_4_12.25, RSM_5_17.35, RSM_5_17.45, RSM_5_17.25, RSM_2_9.05, RSM_5_17.55, RSM_1_7.95, RSM_5_17.15

- **N-Acetylglutamate**: RSM_1_20.95, RSM_1_21.05, RSM_1_8.15, RSM_3_18.25, RSM_1_28.65, RSM_1_21.15, RSM_1_28.55, RSM_1_20.85, RSM_1_8.05, RSM_3_18.15, RSM_3_9.55, RSM_1_8.25, RSM_1_28.45, RSM_3_9.65, RSM_1_28.75, RSM_1_21.25, RSM_3_18.35, RSM_1_19.25, RSM_1_13.45, RSM_5_24.75, RSM_1_19.35, RSM_1_19.15, RSM_1_13.25, RSM_3_9.45, RSM_5_25.35, RSM_5_24.85, RSM_5_25.45, RSM_5_24.65, RSM_1_11.05, RSM_1_16.75, RSM_3_18.05, RSM_1_13.15, RSM_1_8.35, RSM_1_16.85, RSM_1_6.85, RSM_1_13.55, RSM_1_6.75, RSM_1_18.75, RSM_1_20.75, RSM_1_7.05, RSM_1_28.85, RSM_5_24.95, RSM_1_6.65, RSM_1_28.35

- **Nicotinate**: RSM_5_7.85, RSM_5_7.75, RSM_3_8.25, RSM_5_19.15, RSM_4_8.95, RSM_4_8.85, RSM_5_19.05, RSM_1_8.35, RSM_1_18.15, RSM_4_1.45, RSM_1_8.25, RSM_4_1.55, RSM_4_9.05, RSM_1_4.75, RSM_4_1.35, RSM_1_18.25, RSM_5_7.95, RSM_5_7.65, RSM_4_1.25, RSM_1_18.05, RSM_5_18.25, RSM_1_18.35, RSM_2_18.45, RSM_1_4.85, RSM_3_8.35, RSM_4_1.15, RSM_3_8.15, RSM_5_18.15, RSM_1_6.95, RSM_1_8.15, RSM_1_18.45, RSM_4_9.15, RSM_5_7.35, RSM_5_19.25, RSM_5_8.15, RSM_4_1.05, RSM_5_8.05, RSM_1_4.65, RSM_5_18.05, RSM_5_8.25, RSM_1_17.95, RSM_5_7.55, RSM_2_19.35, RSM_1_17.85, RSM_5_7.45, RSM_2_18.55, RSM_1_23.45, RSM_4_9.25, RSM_3_8.45, RSM_2_19.55, RSM_1_7.05, RSM_1_17.75, RSM_1_4.55, RSM_3_10.85, RSM_5_24.45, RSM_2_19.45, RSM_1_23.35, RSM_1_24.25, RSM_2_19.65, RSM_4_9.35, RSM_5_24.65, RSM_1_8.45, RSM_4_9.85, RSM_1_17.65, RSM_3_15.15, RSM_1_1.65, RSM_4_9.95, RSM_1_23.25, RSM_4_9.75, RSM_2_19.25, RSM_1_18.55, RSM_3_8.65, RSM_3_14.95, RSM_1_4.45, RSM_4_9.45, RSM_1_6.55, RSM_3_8.05, RSM_3_8.55

- **Pentonate**: RSM_5_9.35, RSM_5_26.25, RSM_4_14.95, RSM_2_6.65, RSM_3_2.65, RSM_3_11.15, RSM_4_2.65, RSM_3_11.25, RSM_3_2.75, RSM_2_9.75, RSM_2_9.65, RSM_4_2.75, RSM_5_6.35, RSM_5_6.45, RSM_5_16.65, RSM_5_25.35, RSM_3_15.25, RSM_2_7.05, RSM_4_15.05, RSM_3_2.55, RSM_1_16.85, RSM_4_15.35, RSM_3_10.75, RSM_2_5.95, RSM_5_21.65, RSM_2_7.15, RSM_1_25.85, RSM_1_25.95, RSM_3_15.45,

RSM_1_7.85, RSM_5_21.75, RSM_2_6.05, RSM_1_7.75, RSM_3_18.55, RSM_4_12.65, RSM_3_2.85, RSM_1_7.95

- **Phenylalanine**: RSM_1_28.85, RSM_3_18.65, RSM_1_28.95, RSM_3_18.75, RSM_3_18.55, RSM_1_29.35, RSM_1_29.05, RSM_5_5.95, RSM_5_5.85, RSM_1_28.75, RSM_1_29.45, RSM_3_18.45, RSM_1_8.05, RSM_1_8.15, RSM_3_18.05, RSM_4_18.25, RSM_4_18.15, RSM_3_18.35, RSM_3_18.85, RSM_1_28.65, RSM_5_3.15, RSM_5_3.25, RSM_4_18.35, RSM_1_29.25, RSM_5_6.05, RSM_3_17.75, RSM_3_17.65, RSM_5_5.75, RSM_3_18.15, RSM_5_3.05, RSM_4_18.45, RSM_1_8.25, RSM_3_18.25, RSM_5_3.35, RSM_1_12.65, RSM_1_12.75, RSM_1_12.85, RSM_5_2.95, RSM_1_12.95, RSM_3_17.85

- **Phosphoric-acid**: RSM_5_23.75, RSM_5_23.85, RSM_5_23.65, RSM_5_25.55, RSM_5_23.95, RSM_5_25.45, RSM_5_25.65, RSM_5_23.55, RSM_5_24.05, RSM_4_18.25, RSM_5_25.35, RSM_4_18.15, RSM_4_18.35, RSM_5_23.45, RSM_4_18.45, RSM_4_18.55, RSM_5_25.75, RSM_5_4.85, RSM_5_24.15, RSM_5_24.45, RSM_5_4.95, RSM_5_21.45, RSM_5_21.35, RSM_5_23.35, RSM_5_21.85, RSM_5_21.95

- **Proline**: RSM_5_24.95, RSM_5_24.85, RSM_5_25.05, RSM_3_17.85, RSM_5_24.75, RSM_3_17.95, RSM_5_25.15, RSM_1_29.35, RSM_3_18.05, RSM_1_29.25, RSM_3_17.75, RSM_5_25.35, RSM_1_29.15, RSM_5_25.25, RSM_4_14.75, RSM_5_25.45, RSM_2_16.35, RSM_1_29.05, RSM_5_24.65, RSM_4_14.65, RSM_2_16.45, RSM_5_8.65, RSM_2_16.25, RSM_3_17.65, RSM_4_1.45, RSM_4_14.85, RSM_4_1.55, RSM_4_1.35, RSM_1_29.45, RSM_1_28.95, RSM_4_14.55, RSM_4_1.25, RSM_3_16.85, RSM_1_28.85, RSM_3_16.95, RSM_2_16.55, RSM_3_8.75, RSM_3_18.15, RSM_4_1.15, RSM_4_14.45, RSM_3_17.55, RSM_3_16.75, RSM_1_28.75, RSM_3_17.05, RSM_4_14.35, RSM_3_16.35, RSM_1_19.25, RSM_3_16.15, RSM_5_25.55, RSM_1_19.15, RSM_3_16.05, RSM_3_16.25, RSM_4_14.25, RSM_5_8.75, RSM_3_15.95, RSM_3_8.65, RSM_5_4.55, RSM_1_19.35, RSM_4_12.25, RSM_4_1.05, RSM_4_5.45, RSM_5_4.65, RSM_1_28.65, RSM_4_12.55, RSM_1_19.05, RSM_2_15.65, RSM_4_5.35, RSM_4_5.55, RSM_4_14.15, RSM_5_4.45, RSM_5_5.85, RSM_3_15.85, RSM_3_8.55, RSM_5_5.95, RSM_5_5.75, RSM_5_4.35, RSM_5_8.85, RSM_3_16.65

- **Putrescine**: RSM_4_12.85, RSM_4_12.95, RSM_4_12.75, RSM_2_0.05, RSM_4_13.05, RSM_5_25.65, RSM_4_12.65, RSM_5_25.55, RSM_2_0.15, RSM_5_25.75, RSM_3_18.45, RSM_3_21.85, RSM_3_18.25, RSM_2_0.25, RSM_4_16.15, RSM_4_16.25, RSM_4_16.35, RSM_4_16.45, RSM_1_30.45, RSM_3_18.35, RSM_4_16.05, RSM_4_13.95, RSM_1_30.35, RSM_3_21.75, RSM_3_18.55, RSM_5_25.45, RSM_1_30.25, RSM_4_16.55, RSM_3_21.95

- **Pyroglutamate**: RSM_2_1.35, RSM_1_3.95, RSM_1_4.65, RSM_5_6.05, RSM_1_3.65, RSM_2_1.45, RSM_3_11.05, RSM_3_10.95, RSM_5_6.15, RSM_5_6.35, RSM_2_17.95, RSM_5_6.25, RSM_1_3.55, RSM_2_18.05, RSM_2_17.85, RSM_1_4.05, RSM_1_3.85, RSM_5_6.45, RSM_3_11.15

- **Pyruvate**: RSM_5_25.55, RSM_5_25.45, RSM_4_1.15, RSM_4_1.25, RSM_5_25.65, RSM_4_1.05, RSM_5_25.35, RSM_4_1.35, RSM_5_25.75, RSM_4_0.85, RSM_4_0.95, RSM_4_5.85, RSM_2_10.25, RSM_4_5.95, RSM_4_0.75, RSM_3_8.55, RSM_3_8.45, RSM_2_10.15, RSM_5_8.05, RSM_5_7.55, RSM_1_12.75, RSM_5_7.95, RSM_5_7.65, RSM_1_12.85, RSM_3_8.65, RSM_1_12.65, RSM_5_7.45, RSM_5_8.15, RSM_3_8.35, RSM_1_12.55, RSM_3_23.45, RSM_1_12.95, RSM_5_25.85, RSM_3_8.75, RSM_4_1.45, RSM_2_0.05, RSM_5_7.85, RSM_5_7.75, RSM_3_0.25, RSM_3_23.35, RSM_5_26.05, RSM_5_7.35, RSM_2_0.15, RSM_2_10.05, RSM_3_8.85, RSM_5_8.25, RSM_3_0.15,

RSM_4_5.75, RSM_5_25.95, RSM_3_0.35, RSM_2_15.65, RSM_4_0.55, RSM_3_0.05, RSM_1_13.05, RSM_2_15.05, RSM_2_15.75, RSM_4_1.55, RSM_2_9.55, RSM_3_23.25, RSM_3_8.25, RSM_2_9.65, RSM_2_10.35, RSM_5_25.25, RSM_2_14.95, RSM_4_6.05, RSM_2_0.25, RSM_1_23.95, RSM_2_15.55, RSM_1_23.45, RSM_1_23.55, RSM_1_23.85, RSM_3_0.45, RSM_2_14.85, RSM_1_23.75, RSM_2_9.45

- **Raffinose**: RSM_4_0.65, RSM_3_11.25, RSM_3_11.15, RSM_4_0.55, RSM_2_18.45, RSM_3_11.55, RSM_5_7.85, RSM_5_25.65, RSM_5_7.75, RSM_3_11.85, RSM_5_5.35, RSM_5_25.55, RSM_4_13.15, RSM_4_0.45, RSM_2_15.75, RSM_3_11.75, RSM_5_5.45, RSM_5_5.25, RSM_5_19.25, RSM_2_15.85, RSM_2_7.95, RSM_4_13.05, RSM_5_25.75, RSM_2_2.15, RSM_2_2.05, RSM_5_7.95, RSM_5_19.15, RSM_5_6.95, RSM_5_1.55, RSM_5_1.45, RSM_3_9.45, RSM_4_12.15, RSM_5_6.85, RSM_5_25.45, RSM_3_11.05, RSM_5_1.65, RSM_5_19.05, RSM_5_17.25, RSM_2_18.55, RSM_5_17.35, RSM_5_19.35, RSM_2_16.05, RSM_2_15.95, RSM_4_12.95, RSM_5_25.35, RSM_4_13.25, RSM_4_12.05, RSM_5_17.45, RSM_5_6.75, RSM_5_17.15, RSM_5_1.35, RSM_2_7.75, RSM_3_9.55, RSM_5_17.55

- **Serine**: RSM_5_24.75, RSM_5_24.85, RSM_5_24.65, RSM_5_24.95, RSM_1_28.75, RSM_3_18.15, RSM_3_17.75, RSM_3_18.25, RSM_4_1.15, RSM_3_17.65, RSM_1_28.65, RSM_4_1.05, RSM_3_17.85, RSM_5_24.55, RSM_3_18.05, RSM_1_28.85, RSM_4_1.25, RSM_3_18.35, RSM_1_20.65, RSM_1_20.55, RSM_5_4.55, RSM_5_25.05, RSM_1_28.55, RSM_3_17.55, RSM_4_5.75, RSM_5_4.45, RSM_1_29.45, RSM_1_20.85, RSM_1_29.65, RSM_5_4.15, RSM_5_4.25, RSM_1_29.55, RSM_5_4.65, RSM_3_17.95, RSM_4_1.35, RSM_5_4.35, RSM_1_20.45, RSM_1_20.95, RSM_1_29.75, RSM_3_18.45, RSM_4_13.95, RSM_4_13.85, RSM_1_16.45, RSM_1_16.65, RSM_1_29.85, RSM_1_20.75, RSM_4_13.75, RSM_2_11.95, RSM_5_16.95, RSM_5_25.45, RSM_1_22.25, RSM_1_22.15, RSM_5_25.55, RSM_2_12.45, RSM_2_12.55, RSM_5_17.05, RSM_4_14.05, RSM_5_4.05, RSM_4_0.95, RSM_4_5.85, RSM_1_28.45, RSM_5_16.85, RSM_1_16.35, RSM_1_16.75, RSM_5_25.35, RSM_1_17.95, RSM_1_21.05, RSM_4_14.15

- **Succinate**: RSM_1_1.35, RSM_4_12.95, RSM_4_13.05, RSM_1_1.45, RSM_5_7.65, RSM_1_5.85, RSM_1_5.95, RSM_4_6.35, RSM_5_17.85, RSM_5_17.75, RSM_5_7.55, RSM_4_12.85, RSM_5_7.75, RSM_5_7.95, RSM_5_7.85, RSM_4_6.25, RSM_1_6.05, RSM_4_6.45, RSM_5_8.05, RSM_1_7.05, RSM_1_6.95, RSM_1_5.75, RSM_1_29.05, RSM_1_28.95, RSM_5_7.45, RSM_1_1.25, RSM_4_18.05, RSM_1_27.55, RSM_5_17.05, RSM_4_17.35, RSM_5_16.95, RSM_4_12.75, RSM_5_7.35, RSM_4_17.95, RSM_1_0.45, RSM_1_0.35, RSM_5_17.95, RSM_1_29.15, RSM_4_17.85, RSM_1_1.15, RSM_4_17.75, RSM_5_17.15, RSM_4_6.15, RSM_4_12.65, RSM_5_16.85

- **Sucrose**: RSM_5_7.85, RSM_5_7.75, RSM_5_25.55, RSM_5_25.65, RSM_5_7.95, RSM_5_25.45, RSM_3_10.95, RSM_3_11.05, RSM_5_25.75, RSM_1_3.65, RSM_3_10.85, RSM_3_11.25, RSM_3_11.15, RSM_5_25.35, RSM_1_9.45, RSM_1_24.85, RSM_1_3.75, RSM_4_17.75, RSM_3_9.55, RSM_1_25.05, RSM_1_9.35, RSM_1_9.25, RSM_1_3.85, RSM_1_3.95, RSM_5_8.05, RSM_3_10.15, RSM_1_25.15, RSM_3_9.65, RSM_4_16.35, RSM_4_16.45, RSM_4_17.85, RSM_3_9.45, RSM_1_26.35, RSM_2_0.05, RSM_4_17.95

- **Threonate**: RSM_5_26.35, RSM_5_26.25, RSM_5_26.45, RSM_5_18.45, RSM_1_23.85, RSM_1_23.75, RSM_4_7.15, RSM_2_13.75, RSM_2_13.65, RSM_5_26.05, RSM_5_26.15, RSM_4_7.25, RSM_1_30.45, RSM_5_18.35, RSM_5_18.55, RSM_3_16.55, RSM_2_13.85, RSM_1_30.35, RSM_3_16.45, RSM_3_22.75, RSM_5_18.25, RSM_3_22.85, RSM_1_23.95, RSM_2_9.75, RSM_2_9.65, RSM_4_0.65, RSM_1_30.25,

RSM_5_25.95, RSM_3_16.65, RSM_5_18.15, RSM_5_18.65, RSM_2_10.35, RSM_1_23.65, RSM_4_0.55, RSM_2_13.95, RSM_4_6.85, RSM_5_8.45, RSM_3_16.75, RSM_5_8.55, RSM_4_0.25, RSM_5_26.55, RSM_4_0.35, RSM_3_10.55, RSM_2_10.25, RSM_5_25.85, RSM_1_27.55, RSM_3_22.35, RSM_3_22.25, RSM_2_9.55, RSM_3_22.65, RSM_5_18.05, RSM_2_16.45, RSM_5_8.95, RSM_5_8.85, RSM_1_19.25, RSM_2_16.55, RSM_2_16.35, RSM_3_16.35, RSM_1_19.35, RSM_1_19.55, RSM_5_0.95, RSM_5_0.85, RSM_1_19.15, RSM_4_7.35, RSM_4_0.15, RSM_1_19.45, RSM_3_22.95, RSM_5_9.25, RSM_3_0.25, RSM_5_8.35, RSM_2_14.05, RSM_1_28.05

- **Threonine**: RSM_5_24.75, RSM_5_24.85, RSM_5_24.65, RSM_1_28.75, RSM_1_28.65, RSM_5_24.95, RSM_1_28.85, RSM_3_17.75, RSM_1_28.55, RSM_3_18.05, RSM_3_17.85, RSM_3_17.65, RSM_3_18.15, RSM_4_14.05, RSM_3_18.45, RSM_3_18.65, RSM_3_18.55, RSM_1_28.95, RSM_4_14.15, RSM_1_4.85, RSM_4_18.25, RSM_1_28.45, RSM_3_17.95, RSM_4_14.25, RSM_4_13.95, RSM_3_18.75, RSM_1_4.75, RSM_3_18.25, RSM_4_18.15, RSM_5_25.05, RSM_1_4.95, RSM_3_18.35, RSM_4_14.35, RSM_5_24.55, RSM_5_25.45, RSM_2_0.05, RSM_4_18.35, RSM_1_5.05, RSM_1_29.05, RSM_4_14.45, RSM_2_0.15, RSM_1_20.85, RSM_5_25.35, RSM_1_5.15, RSM_3_17.55, RSM_1_20.95, RSM_4_13.85, RSM_4_14.55, RSM_1_1.25, RSM_5_25.55, RSM_1_20.75, RSM_1_28.35, RSM_2_11.85, RSM_2_0.25, RSM_4_18.45, RSM_4_12.55, RSM_2_11.95, RSM_4_14.65, RSM_1_20.65, RSM_3_18.85, RSM_1_1.35, RSM_4_13.75, RSM_5_16.95, RSM_1_21.05, RSM_3_5.65, RSM_3_5.75, RSM_5_25.65, RSM_2_1.05, RSM_1_1.15, RSM_4_14.75, RSM_1_1.45, RSM_5_17.05, RSM_1_6.85, RSM_5_25.15, RSM_5_16.85, RSM_2_0.95, RSM_1_6.75, RSM_2_0.85, RSM_3_1.95, RSM_3_2.05, RSM_5_25.25, RSM_1_29.15

- **Trans-Sinapinate**: RSM_5_24.85, RSM_5_24.75, RSM_4_0.35, RSM_4_0.65, RSM_5_24.95, RSM_4_0.45, RSM_5_24.65, RSM_4_0.55, RSM_5_25.05, RSM_4_0.25, RSM_1_0.45, RSM_3_17.75, RSM_4_0.75, RSM_1_0.35, RSM_1_0.55, RSM_1_12.35, RSM_1_12.25, RSM_5_25.15, RSM_3_17.85, RSM_3_17.65, RSM_2_1.15, RSM_1_0.25, RSM_1_12.45, RSM_1_22.45, RSM_1_5.65, RSM_2_1.05, RSM_1_22.35, RSM_1_12.15, RSM_1_5.55, RSM_2_11.25, RSM_5_9.05, RSM_2_11.35, RSM_1_18.35, RSM_5_24.55, RSM_1_18.25, RSM_1_0.65, RSM_1_22.55, RSM_5_9.15, RSM_1_5.75, RSM_1_24.75, RSM_2_11.45, RSM_2_11.15, RSM_2_1.35, RSM_1_0.15, RSM_1_24.65, RSM_4_0.85, RSM_1_30.45, RSM_1_30.35, RSM_1_30.25, RSM_2_11.05, RSM_1_12.05, RSM_1_18.45, RSM_1_30.15, RSM_1_22.25, RSM_2_17.05, RSM_2_16.95, RSM_3_2.15, RSM_3_2.25, RSM_2_10.95, RSM_2_11.55, RSM_1_0.05, RSM_1_18.15, RSM_2_10.85, RSM_1_22.95, RSM_1_30.05, RSM_1_18.55, RSM_3_2.05, RSM_1_5.85, RSM_2_17.15, RSM_5_9.25, RSM_3_17.95, RSM_1_12.55, RSM_2_16.85, RSM_5_8.95, RSM_2_0.95, RSM_3_2.35, RSM_2_7.15, RSM_2_7.05, RSM_1_23.75, RSM_2_18.05, RSM_1_23.85, RSM_4_0.15, RSM_2_18.15, RSM_1_5.45, RSM_2_17.95, RSM_2_10.75, RSM_4_13.75, RSM_3_1.95, RSM_1_29.95, RSM_5_24.45, RSM_2_1.45, RSM_1_22.15, RSM_1_18.65, RSM_5_24.35, RSM_2_17.85, RSM_4_13.85, RSM_4_13.65, RSM_5_26.25, RSM_1_24.85, RSM_3_2.45, RSM_2_18.25, RSM_5_25.25, RSM_1_5.95, RSM_1_22.65, RSM_2_11.65, RSM_1_17.95, RSM_2_17.25, RSM_4_12.15, RSM_4_13.55, RSM_5_26.35, RSM_5_24.25, RSM_3_11.05, RSM_3_10.95, RSM_1_23.05, RSM_2_7.45, RSM_4_12.25, RSM_2_17.75, RSM_5_8.85, RSM_2_16.75, RSM_1_18.05, RSM_3_17.55, RSM_1_22.85, RSM_2_7.55, RSM_1_5.35, RSM_5_24.15, RSM_4_0.95

- **Trehalose**: RSM_5_7.75, RSM_5_7.85, RSM_5_7.65, RSM_5_7.55, RSM_5_7.35, RSM_5_7.45, RSM_5_7.95, RSM_5_7.25, RSM_5_8.05, RSM_5_8.15, RSM_5_7.15,

RSM_1_18.95, RSM_5_8.25, RSM_1_19.05, RSM_1_18.75, RSM_5_8.35, RSM_5_7.05, RSM_5_9.45, RSM_1_18.65, RSM_5_9.55, RSM_5_9.15, RSM_1_19.15, RSM_5_9.35, RSM_5_9.05, RSM_5_9.25, RSM_4_8.75, RSM_4_16.85, RSM_4_0.35, RSM_1_18.55, RSM_4_8.85, RSM_5_9.65, RSM_4_16.75, RSM_4_15.15, RSM_5_16.85, RSM_4_8.65, RSM_5_0.55, RSM_5_25.25, RSM_4_15.25, RSM_4_16.65, RSM_4_16.95, RSM_1_4.35, RSM_5_16.75, RSM_5_25.15, RSM_1_4.25, RSM_4_15.05, RSM_4_0.45, RSM_4_8.55, RSM_5_0.65, RSM_5_0.45, RSM_4_8.95, RSM_2_16.35, RSM_4_0.25, RSM_2_16.25, RSM_5_25.05, RSM_5_24.65, RSM_5_17.25, RSM_2_6.85, RSM_2_16.45, RSM_5_17.35, RSM_4_8.45, RSM_1_4.15, RSM_5_24.95, RSM_5_17.15, RSM_5_16.95, RSM_5_8.45, RSM_4_16.55, RSM_5_24.75, RSM_1_4.45, RSM_5_25.35, RSM_5_24.85, RSM_1_22.55, RSM_5_6.95, RSM_2_16.15, RSM_1_22.45, RSM_4_17.05, RSM_5_17.45, RSM_4_8.35, RSM_2_6.75, RSM_5_0.75, RSM_2_6.95, RSM_5_17.05, RSM_5_0.35, RSM_1_5.05, RSM_1_4.95, RSM_2_7.05, RSM_2_16.55, RSM_5_24.55, RSM_4_0.15, RSM_4_8.25, RSM_1_18.45, RSM_1_22.65, RSM_4_9.05, RSM_1_4.05, RSM_1_22.35, RSM_5_1.05, RSM_5_17.55, RSM_5_1.15, RSM_1_4.55, RSM_5_1.25, RSM_5_1.45, RSM_5_1.35, RSM_3_9.75, RSM_1_19.25, RSM_5_16.65, RSM_2_7.15, RSM_4_17.15, RSM_1_22.75, RSM_4_16.45, RSM_3_9.85, RSM_1_30.45, RSM_1_1.25, RSM_4_0.05, RSM_4_15.35, RSM_1_30.35, RSM_3_9.65, RSM_1_5.15, RSM_2_16.05, RSM_5_26.25, RSM_1_4.85, RSM_5_15.95, RSM_2_1.85, RSM_5_24.45, RSM_1_30.25, RSM_5_16.05, RSM_5_15.85, RSM_2_1.95, RSM_2_1.75, RSM_5_16.15, RSM_1_22.85, RSM_1_28.25, RSM_5_17.75, RSM_3_9.95, RSM_5_0.25, RSM_5_17.85, RSM_2_6.35

- **Tyrosine**: RSM_5_9.05, RSM_5_6.85, RSM_5_6.95, RSM_5_9.15, RSM_5_6.75, RSM_5_7.95, RSM_1_28.05, RSM_5_8.05, RSM_1_27.95, RSM_5_6.65, RSM_5_8.15, RSM_3_17.55, RSM_1_27.75, RSM_5_7.25, RSM_1_28.85, RSM_1_27.85, RSM_2_0.05, RSM_3_17.85, RSM_1_28.15, RSM_5_7.05, RSM_3_17.95, RSM_5_8.25, RSM_5_9.25, RSM_1_28.75, RSM_2_18.25, RSM_3_17.65

- **Urea**: RSM_5_25.45, RSM_5_25.55, RSM_5_25.35, RSM_5_25.65, RSM_1_28.65, RSM_1_28.75, RSM_5_25.75, RSM_3_22.15, RSM_4_1.55, RSM_3_8.05, RSM_1_28.55, RSM_1_4.05, RSM_1_4.15

- **Valine**: RSM_5_9.05, RSM_1_20.95, RSM_5_9.15, RSM_5_24.45, RSM_1_20.85, RSM_2_0.95, RSM_2_1.05, RSM_1_20.45, RSM_5_5.35, RSM_5_5.25, RSM_5_7.85, RSM_5_24.75, RSM_5_24.65, RSM_2_0.85, RSM_5_5.45, RSM_5_7.95, RSM_1_28.85, RSM_5_8.95, RSM_5_24.55, RSM_5_25.45, RSM_5_24.35, RSM_1_20.55, RSM_5_5.55, RSM_1_28.75, RSM_1_27.75, RSM_5_25.55, RSM_2_0.05, RSM_1_20.35, RSM_5_7.75, RSM_1_27.65, RSM_5_24.85, RSM_3_11.15, RSM_1_27.85, RSM_5_5.65, RSM_3_11.25, RSM_1_21.05, RSM_2_1.15, RSM_3_11.55, RSM_5_4.55, RSM_5_4.65, RSM_5_8.85, RSM_5_5.15, RSM_2_0.15, RSM_5_9.25, RSM_5_8.05, RSM_5_5.75, RSM_1_28.65, RSM_5_24.25, RSM_2_0.25, RSM_2_1.35, RSM_1_17.75, RSM_1_27.95, RSM_1_17.65, RSM_1_17.85, RSM_1_28.95, RSM_5_4.45, RSM_1_20.65, RSM_1_3.15, RSM_5_25.35, RSM_3_2.55, RSM_5_6.05, RSM_1_3.65, RSM_3_11.05, RSM_3_2.65

- **Xylose**: RSM_3_11.85, RSM_3_18.65, RSM_4_12.75, RSM_2_7.95, RSM_3_11.95, RSM_3_11.75, RSM_3_18.55, RSM_3_18.75, RSM_4_12.65, RSM_4_9.55, RSM_4_12.85, RSM_3_15.65, RSM_3_18.25, RSM_4_13.05, RSM_4_12.95, RSM_2_7.85, RSM_3_11.55, RSM_4_7.75, RSM_4_9.45, RSM_4_9.95, RSM_5_1.65, RSM_3_12.45, RSM_4_9.35, RSM_3_18.45, RSM_5_1.55, RSM_3_18.85, RSM_5_1.75

2 Correspondence between CIM and NCR

QTL detection for each metabolite using CIM and NCR when the guiding dataset is SNP data. The $-\log_{10}$ p-value score for every marker is plotted when CIM is used. The red dotted vertical lines are plotted as a visual separation between the 5 chromosomes and the chromosome number is indicated on the x-axis below each segment. The dotted horizontal blue line marks the $-\log_{10}$ p-value score of 3. Red dots on the x-axis are placed on marker positions for which NCR estimated non-zero coefficients. The color transparency indicates the magnitude of the regularized estimated coefficient.

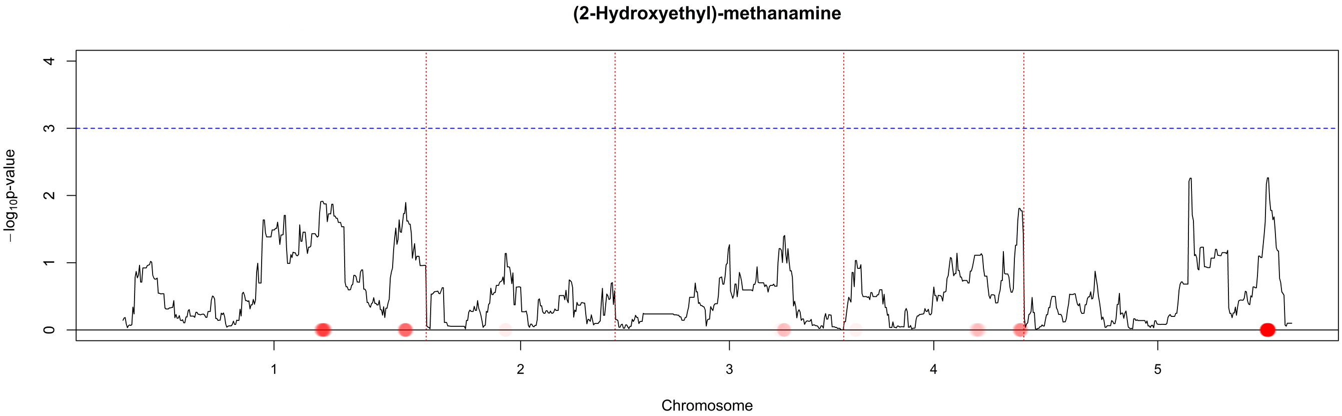
(2-Hydroxyethyl)-methanamine
$-\log_{10}$p-value
0
1
2
3
4
1
2
3
4
5
Chromosome

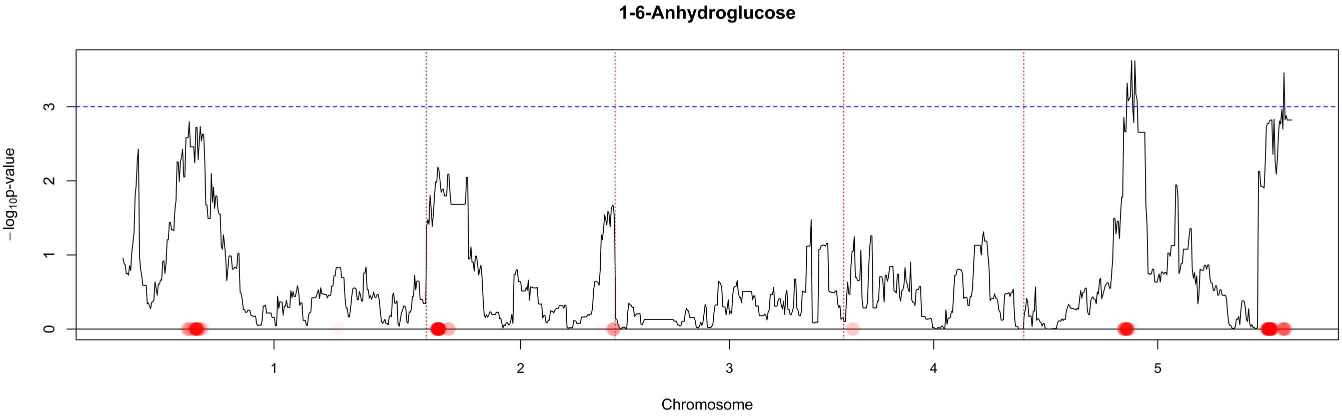
1-6-Anhydroglucose
$-\log_{10}$p-value
0
1
2
3
1
2
3
4
5
Chromosome

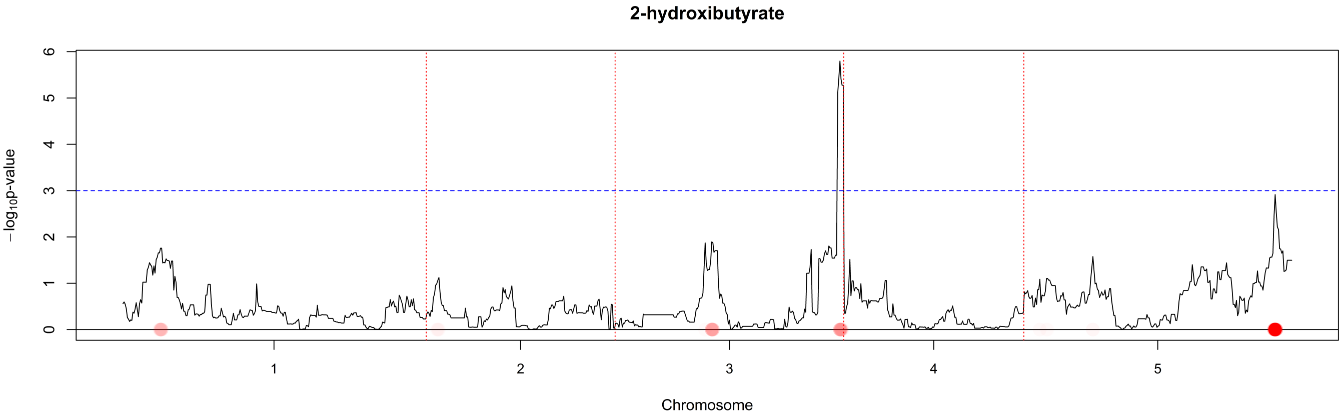
2-hydroxibutyrate
$-\log_{10}$p-value
0
1
2
3
4
5
6
1
2
3
4
5
Chromosome

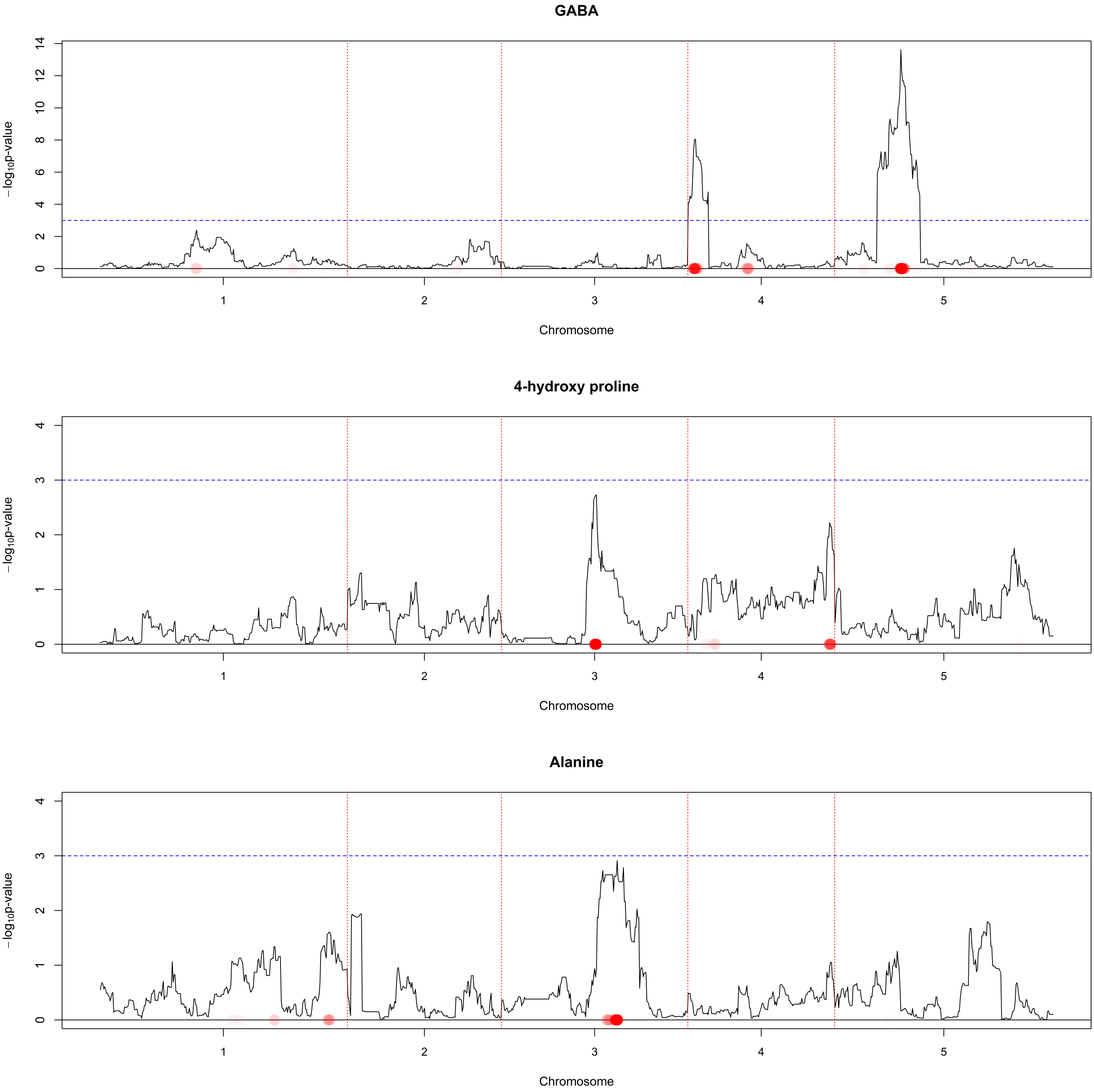
GABA
−log10p-value
Chromosome
4-hydroxy proline
−log10p-value
Chromosome
Alanine
−log10p-value
Chromosome

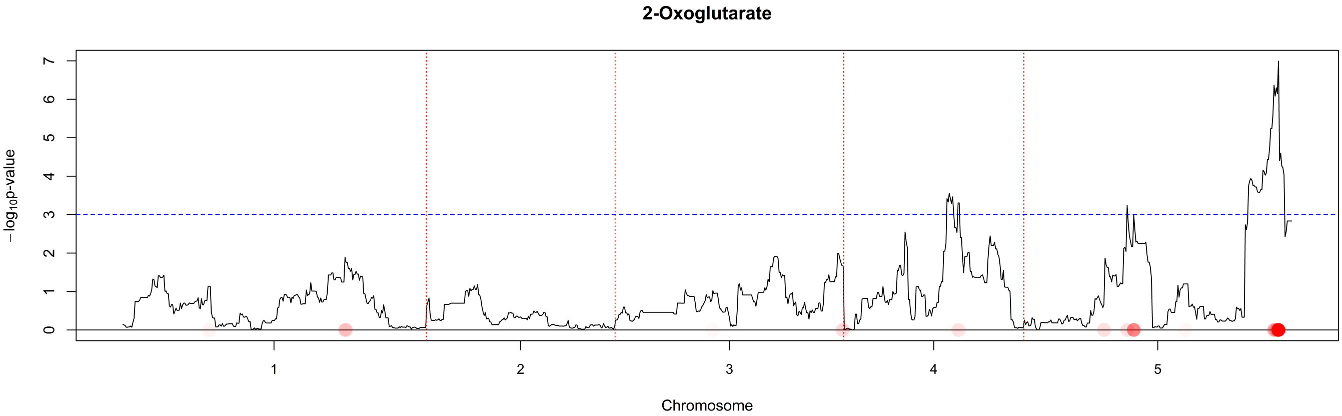
2-Oxoglutarate
−log10p-value
Chromosome
1
2
3
4
5

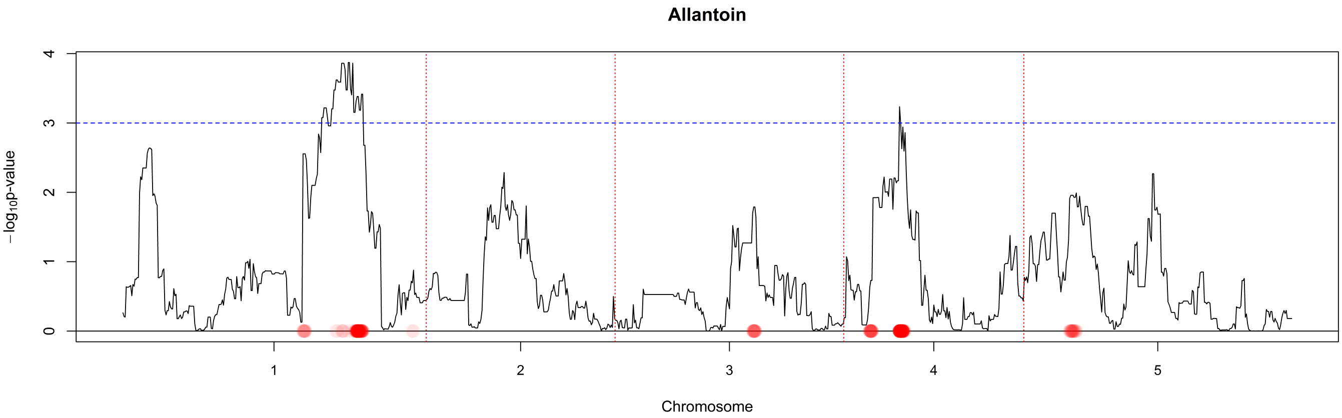
Allantoin
−log10p-value
Chromosome
1
2
3
4
5

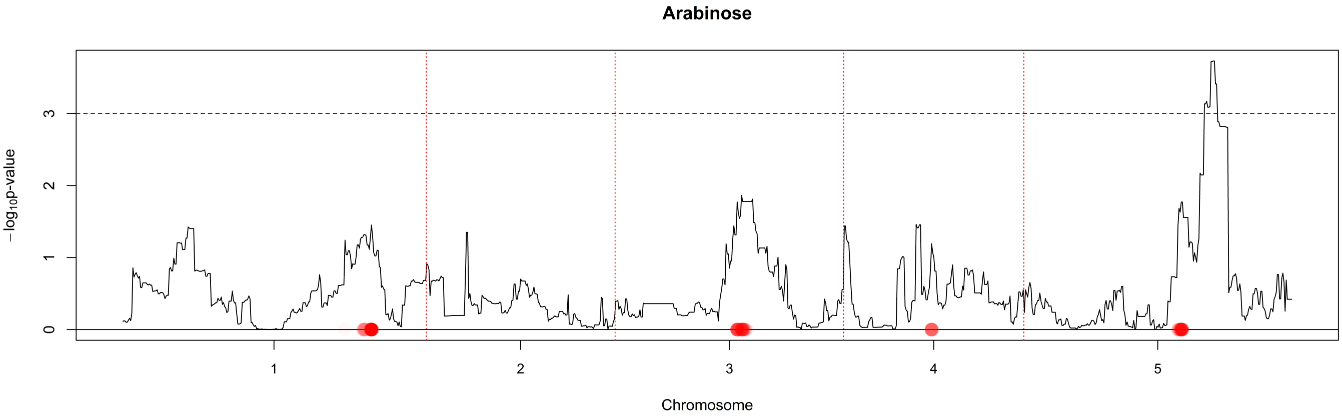
Arabinose
−log10p-value
Chromosome
1
2
3
4
5

Ascorbate

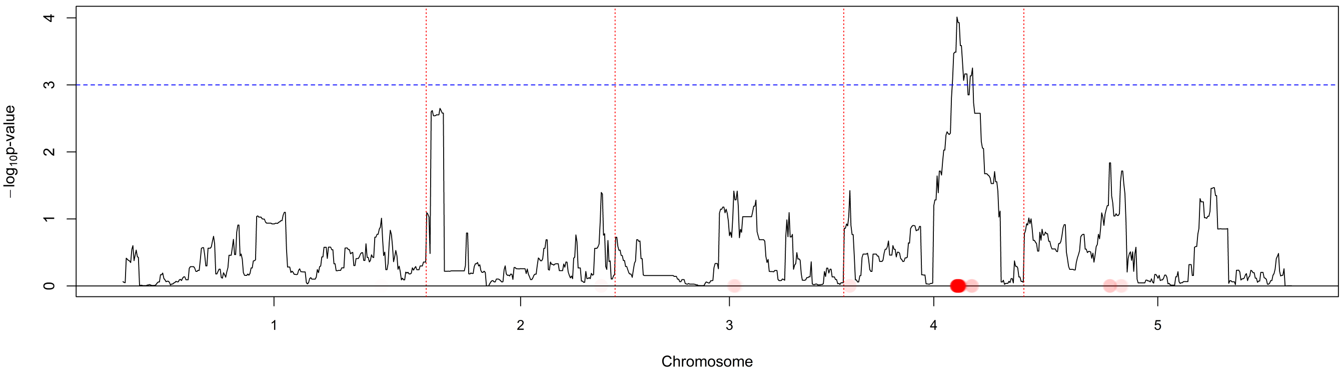

Asparagine

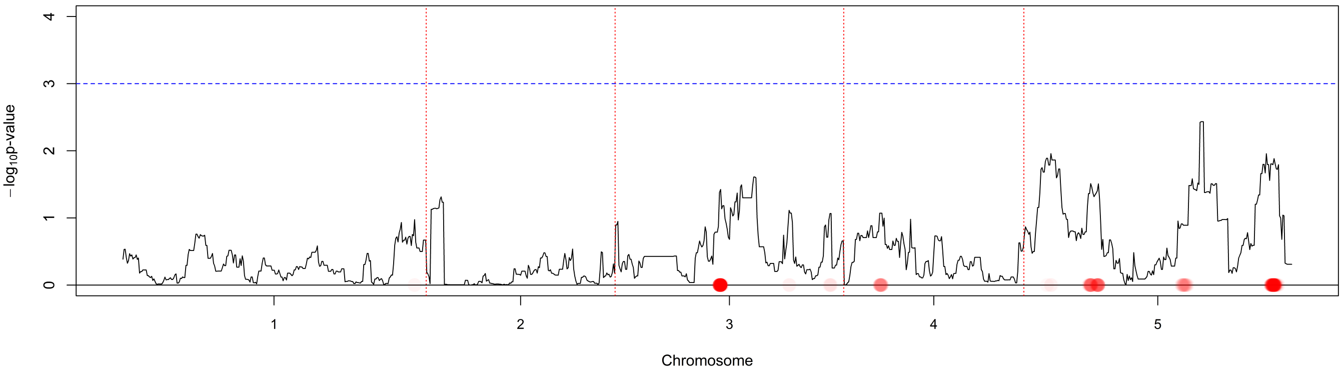

Aspartate

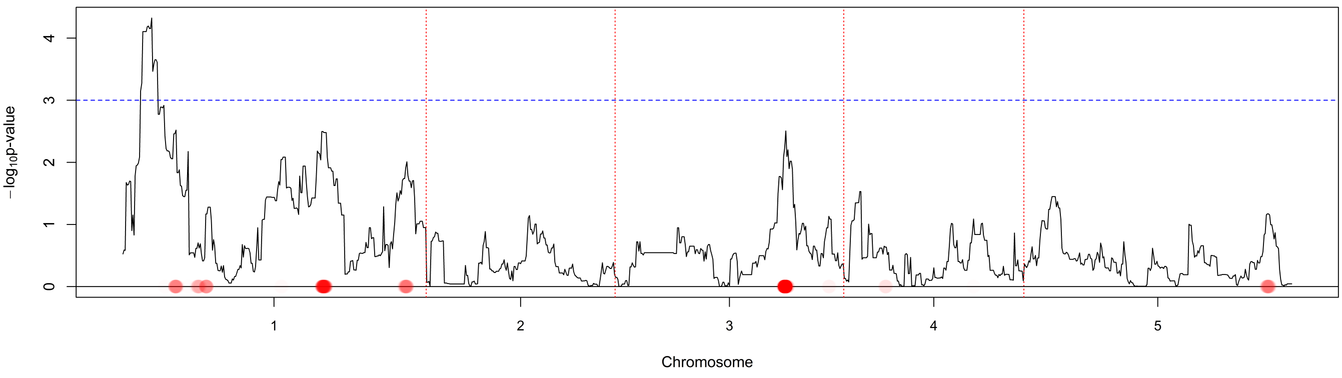

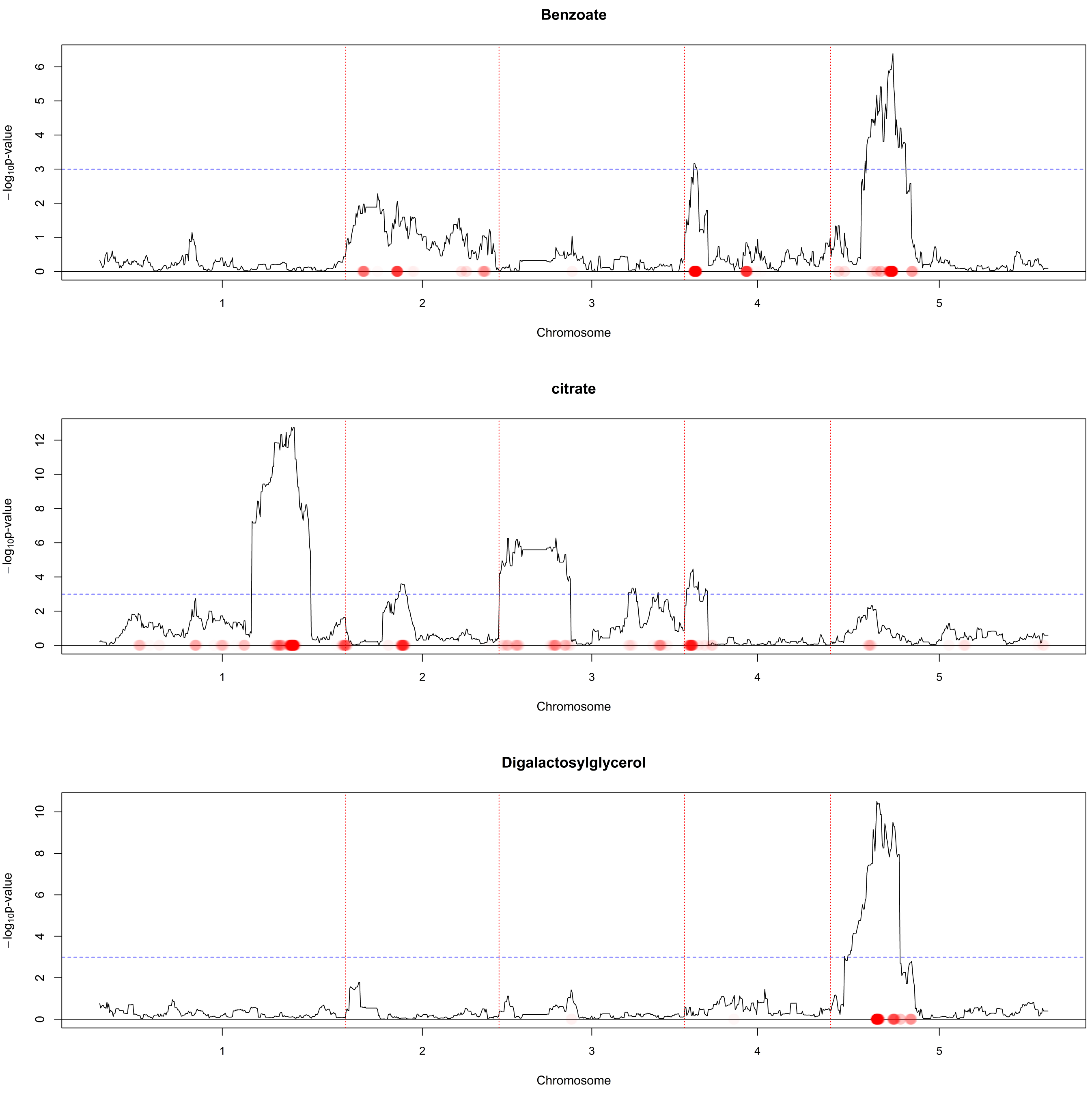
Benzoate
$-\log_{10}$p-value
Chromosome
citrate
$-\log_{10}$p-value
Chromosome
Digalactosylglycerol
$-\log_{10}$p-value
Chromosome

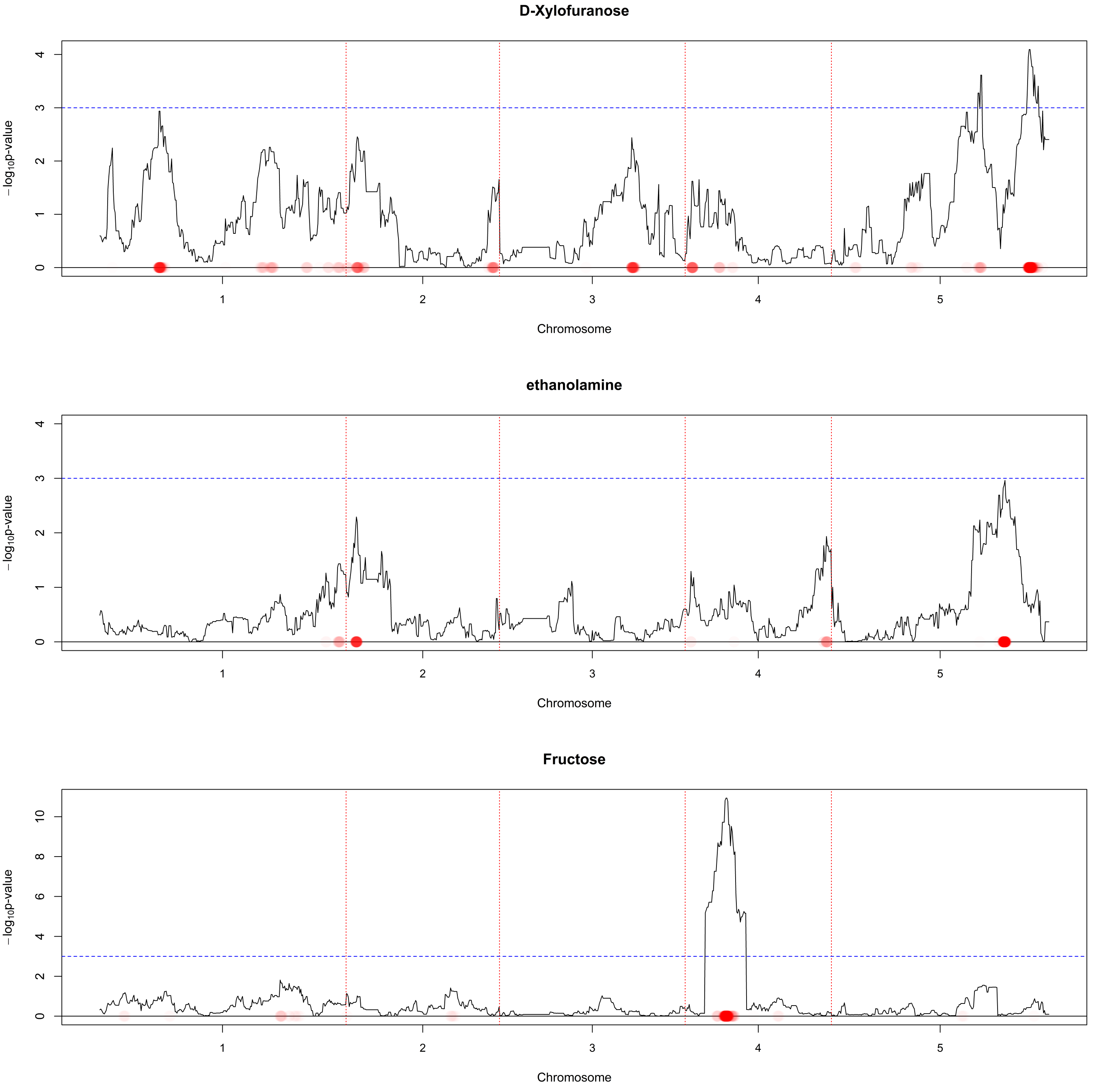
D-Xylofuranose
−log10p-value
Chromosome
ethanolamine
−log10p-value
Chromosome
Fructose
−log10p-value
Chromosome

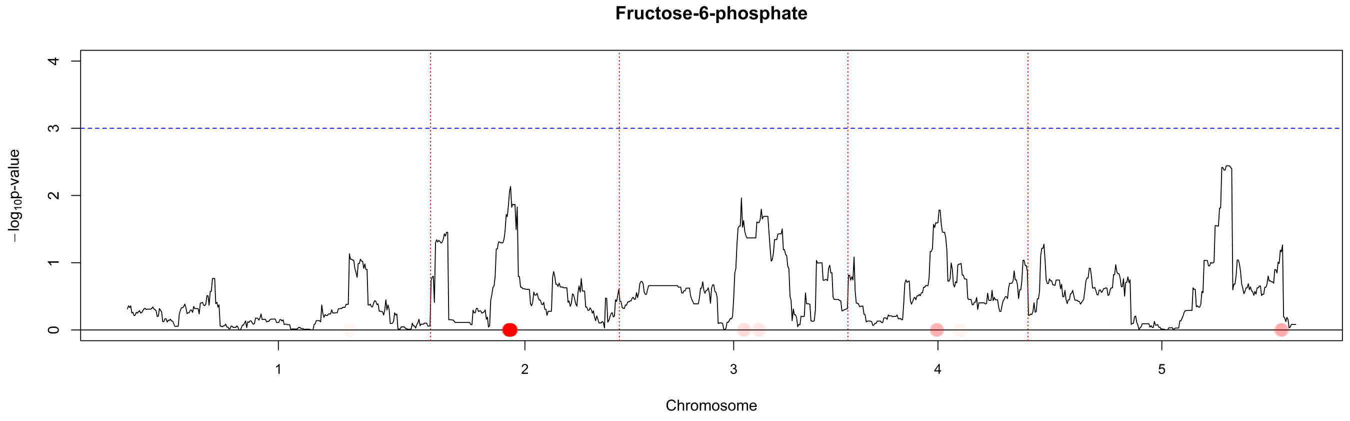
Fructose-6-phosphate
−log10p-value
0
1
2
3
4
1
2
3
4
5
Chromosome

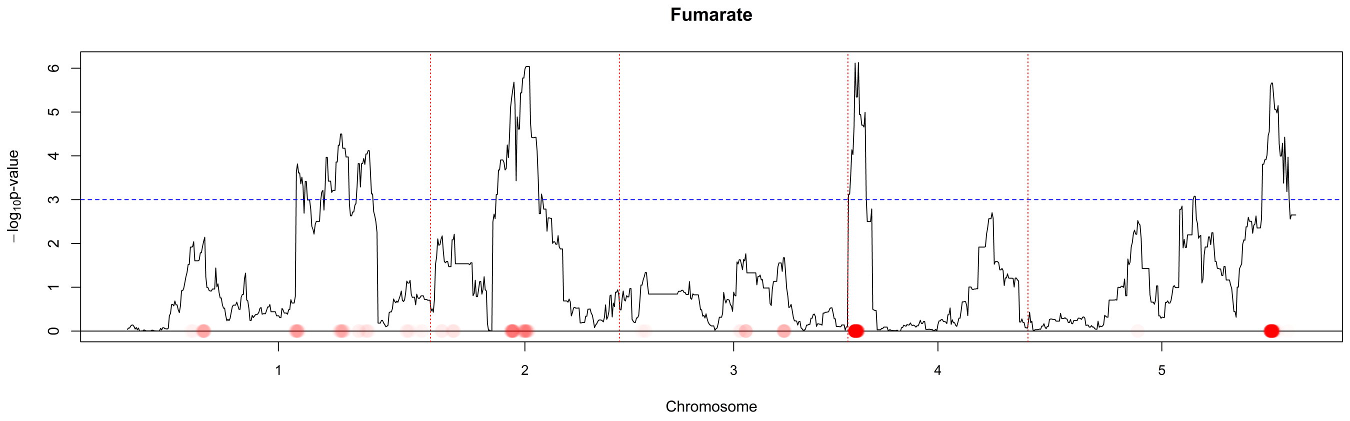
Fumarate
−log10p-value
0
1
2
3
4
5
6
1
2
3
4
5
Chromosome

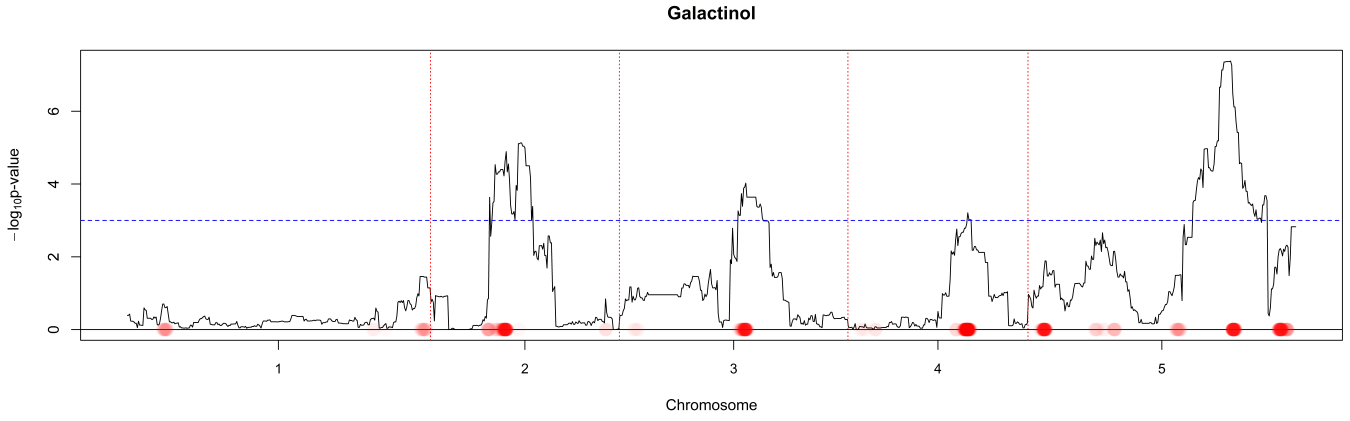
Galactinol
−log10p-value
0
2
4
6
1
2
3
4
5
Chromosome

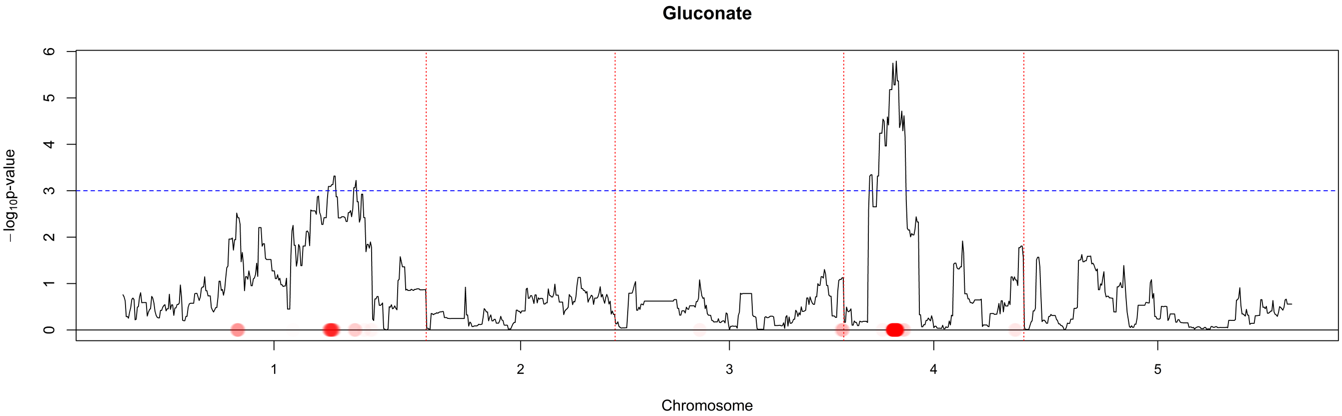
Gluconate
−log10p-value
0
1
2
3
4
5
6
1
2
3
4
5
Chromosome

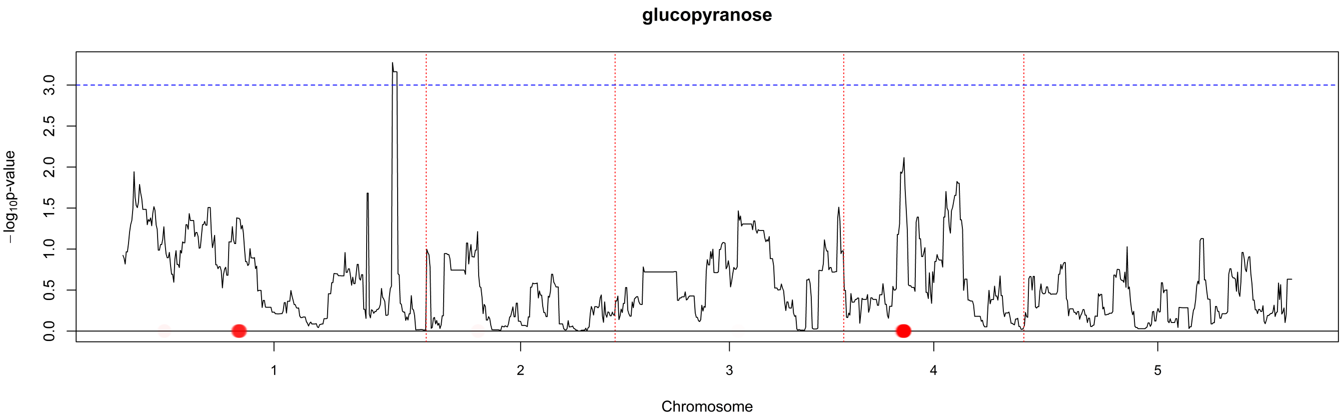
glucopyranose
−log10p-value
0.0
0.5
1.0
1.5
2.0
2.5
3.0
1
2
3
4
5
Chromosome

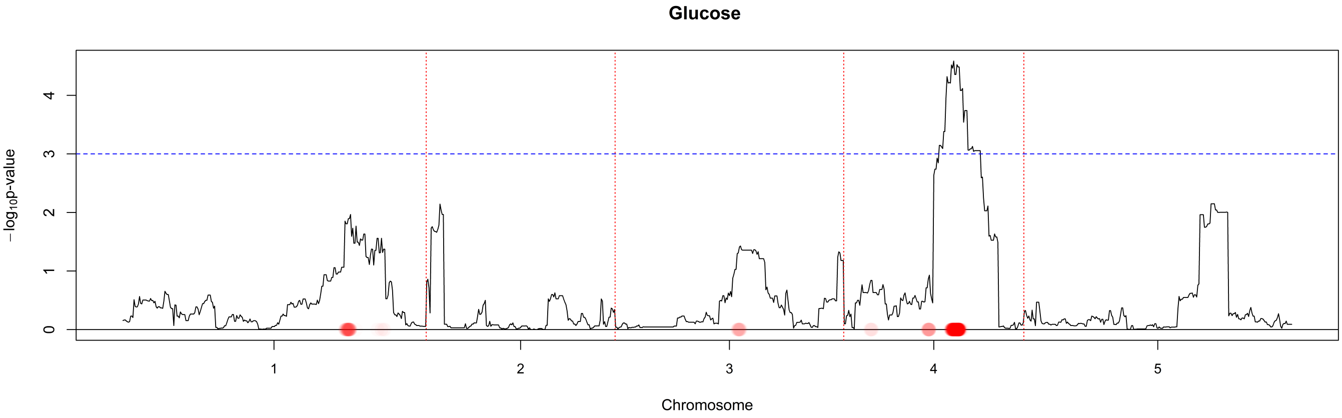
Glucose
−log10p-value
0
1
2
3
4
1
2
3
4
5
Chromosome

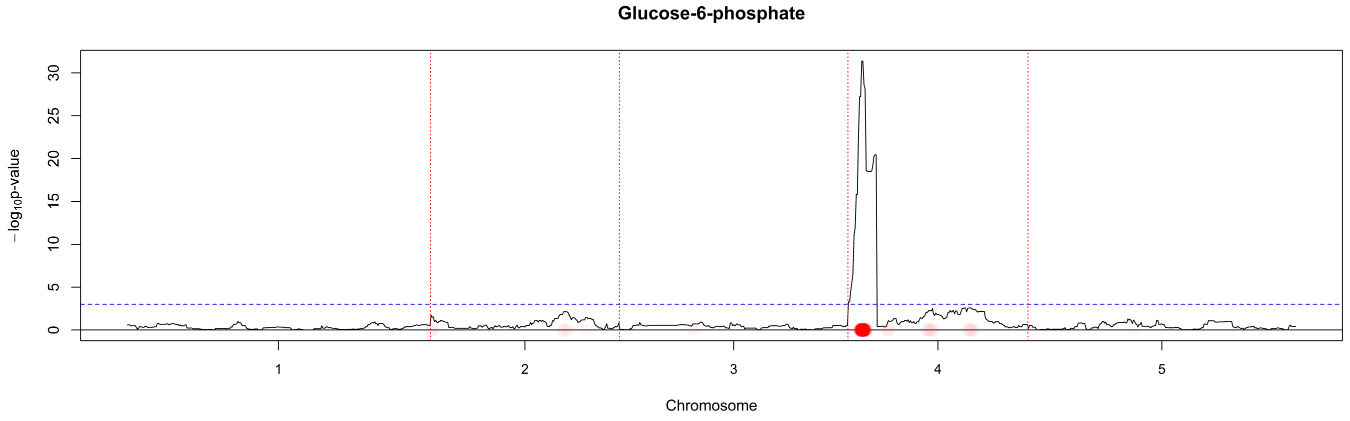
Glucose-6-phosphate
−log10p-value
0
5
10
15
20
25
30
1
2
3
4
5
Chromosome

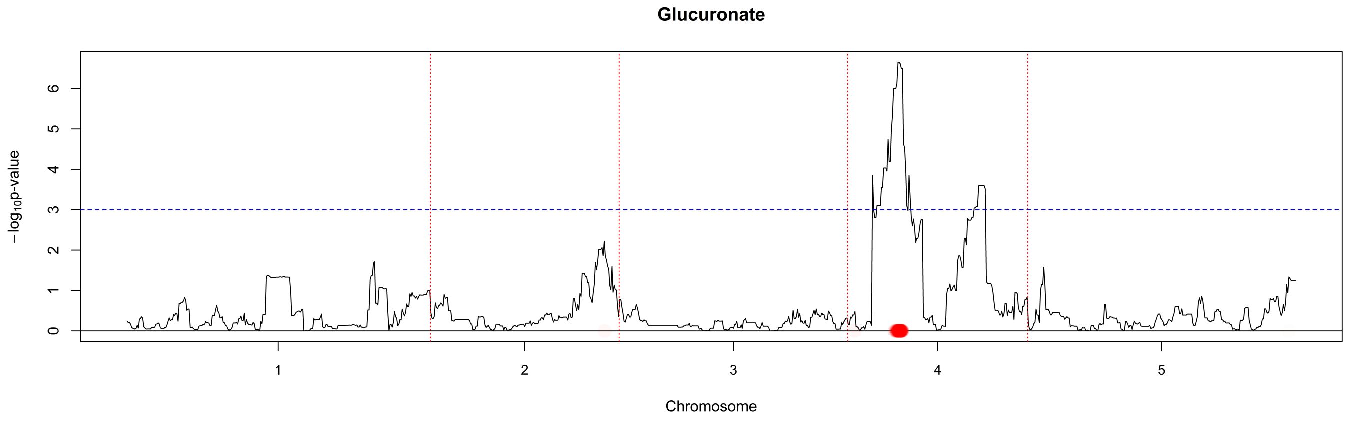
Glucuronate
−log10p-value
0
1
2
3
4
5
6
1
2
3
4
5
Chromosome

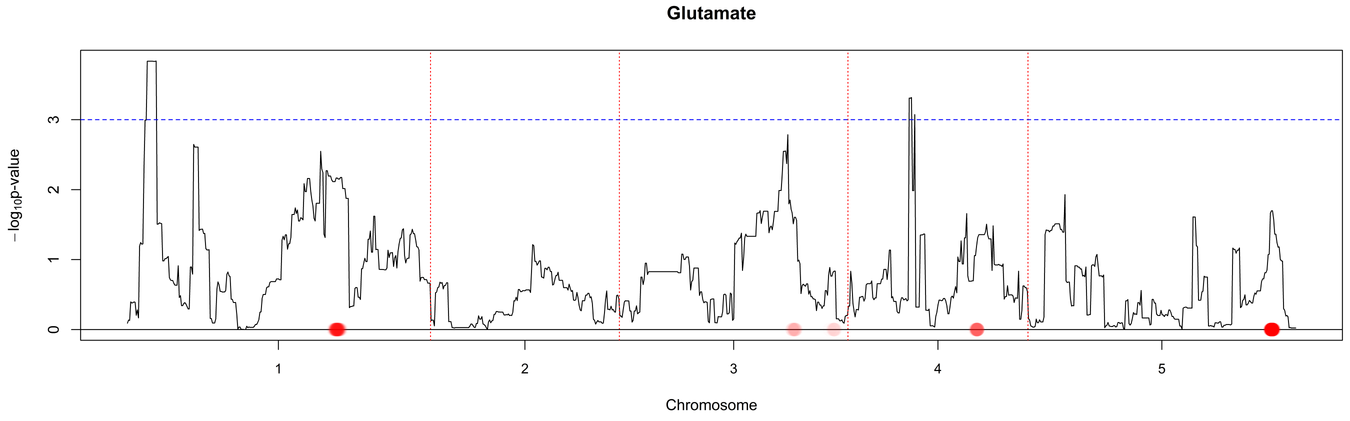
Glutamate
−log10p-value
0
1
2
3
1
2
3
4
5
Chromosome

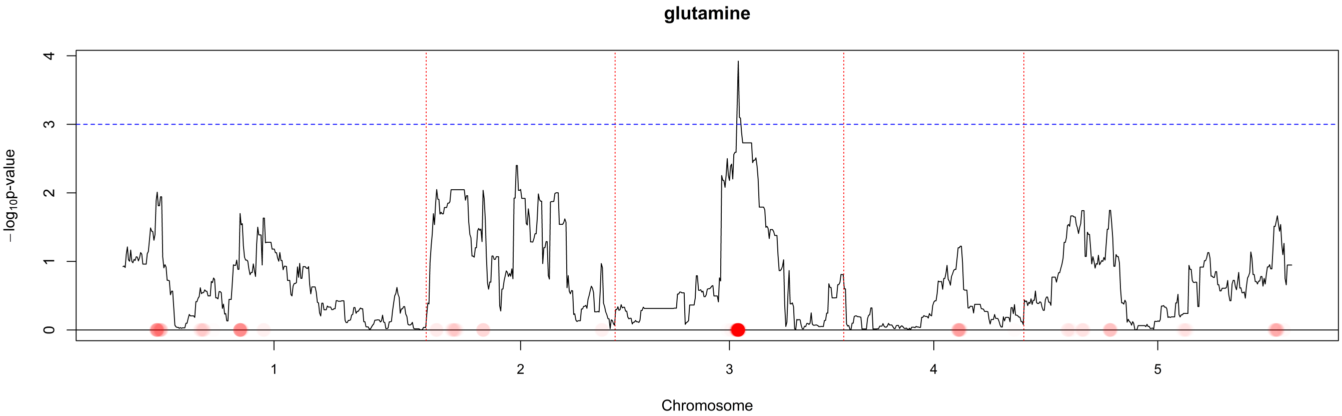
glutamine
−log10p-value
0
1
2
3
4
1
2
3
4
5
Chromosome

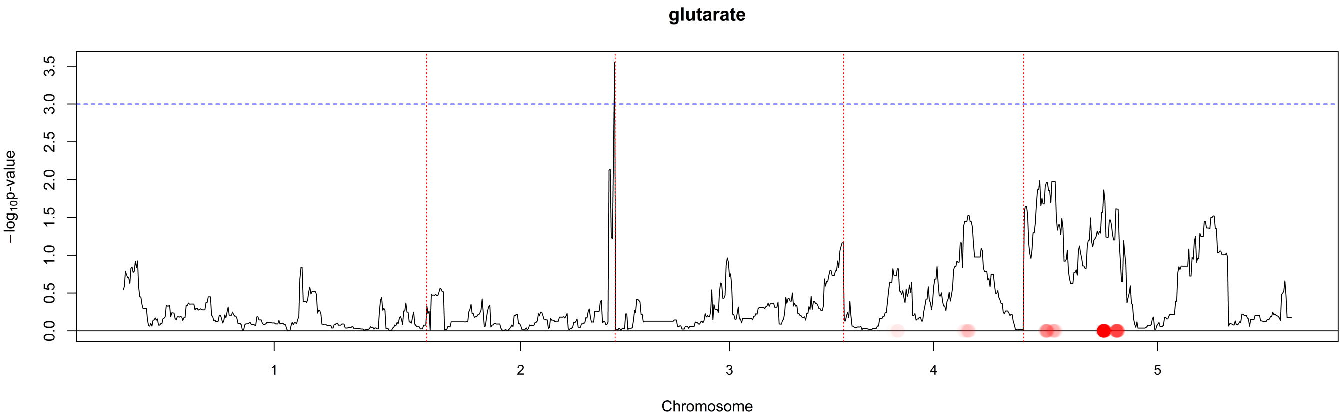
glutarate
−log10p-value
0.0
0.5
1.0
1.5
2.0
2.5
3.0
3.5
1
2
3
4
5
Chromosome

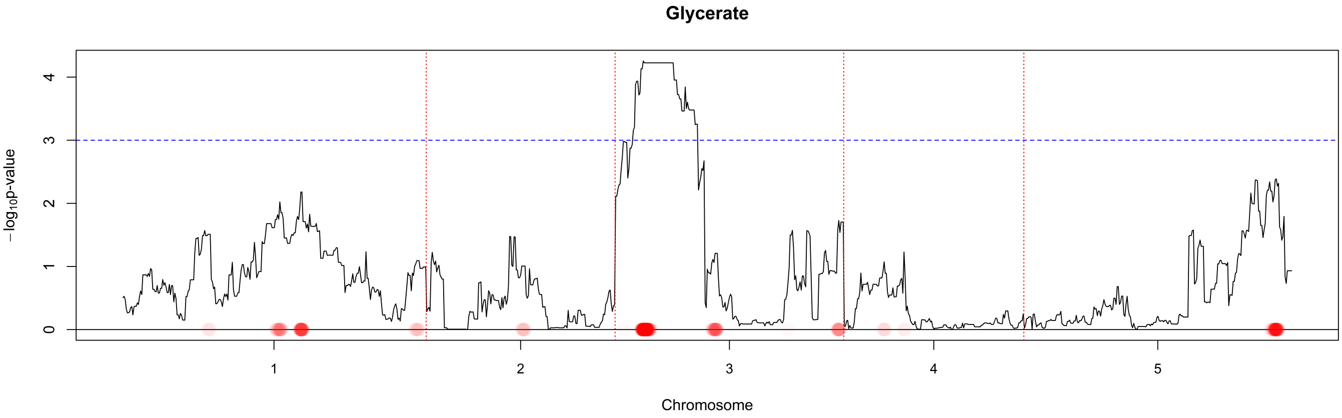
Glycerate
−log10p-value
0
1
2
3
4
1
2
3
4
5
Chromosome

Glycine

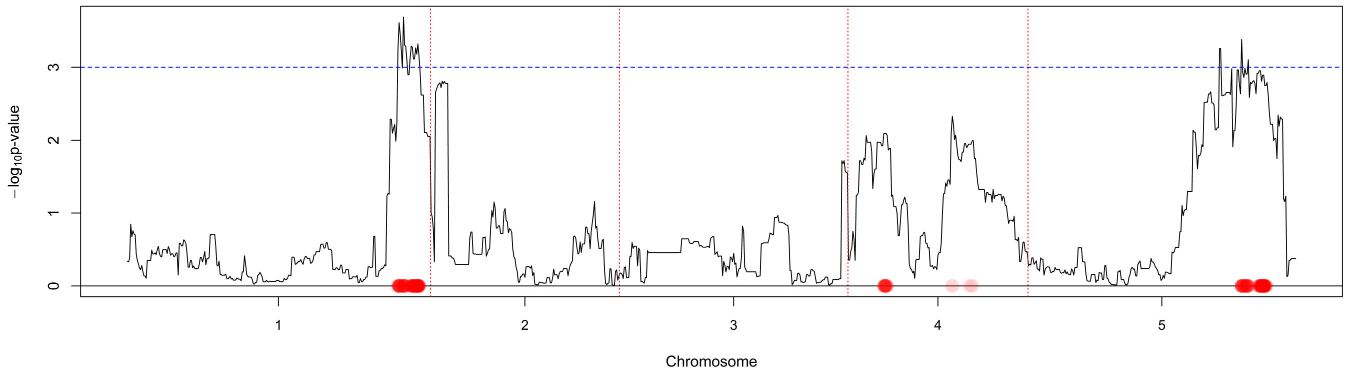

Glycolate

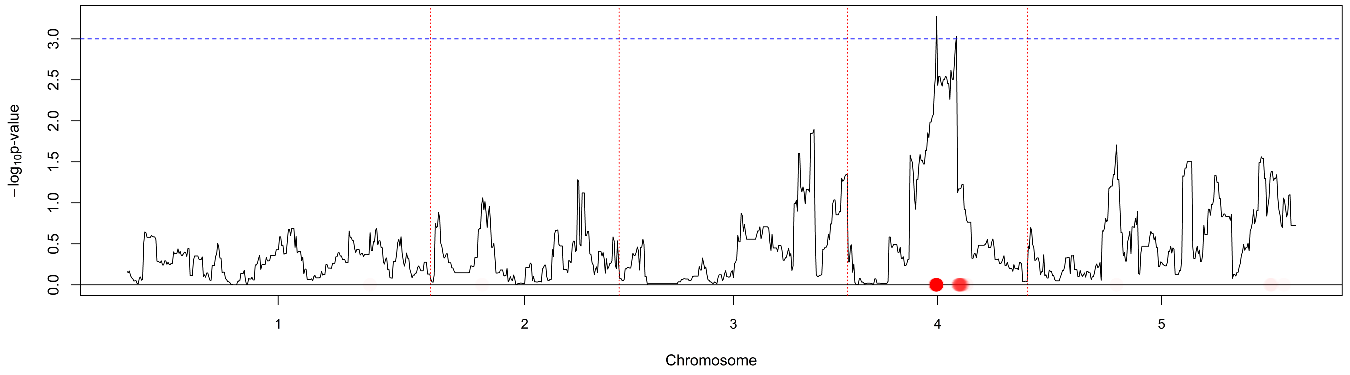

Hexonate

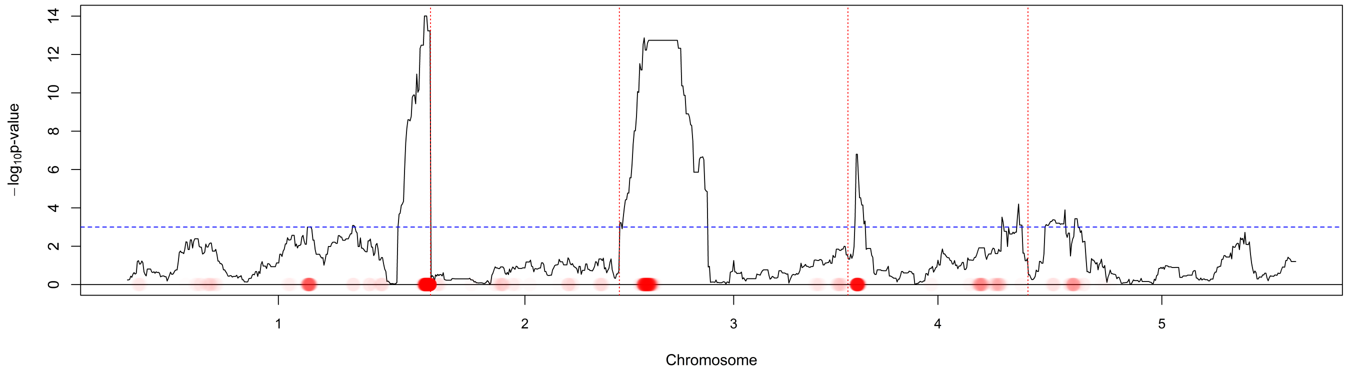

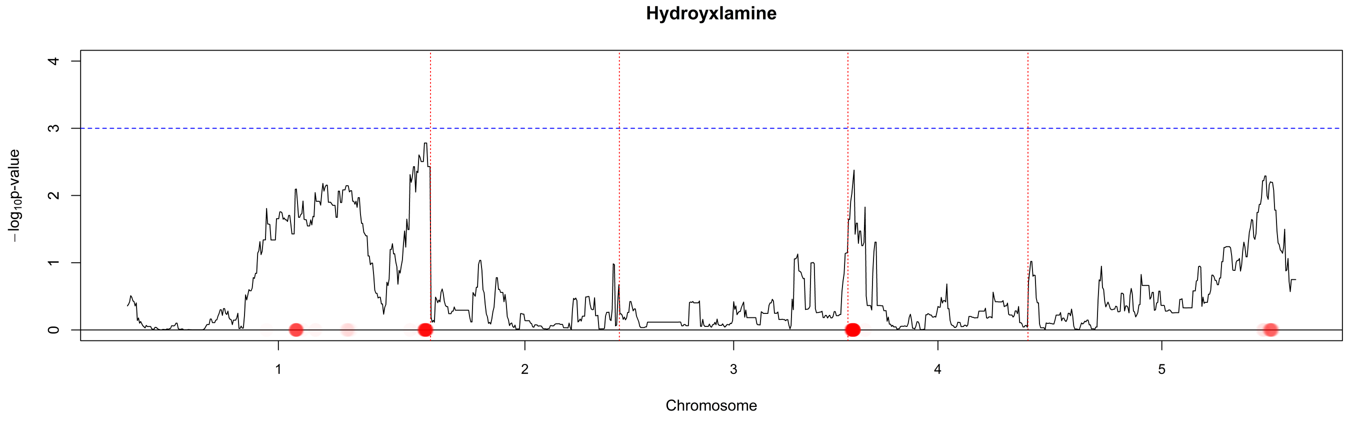
Hydroyxlamine
−log10p-value
0
1
2
3
4
1
2
3
4
5
Chromosome

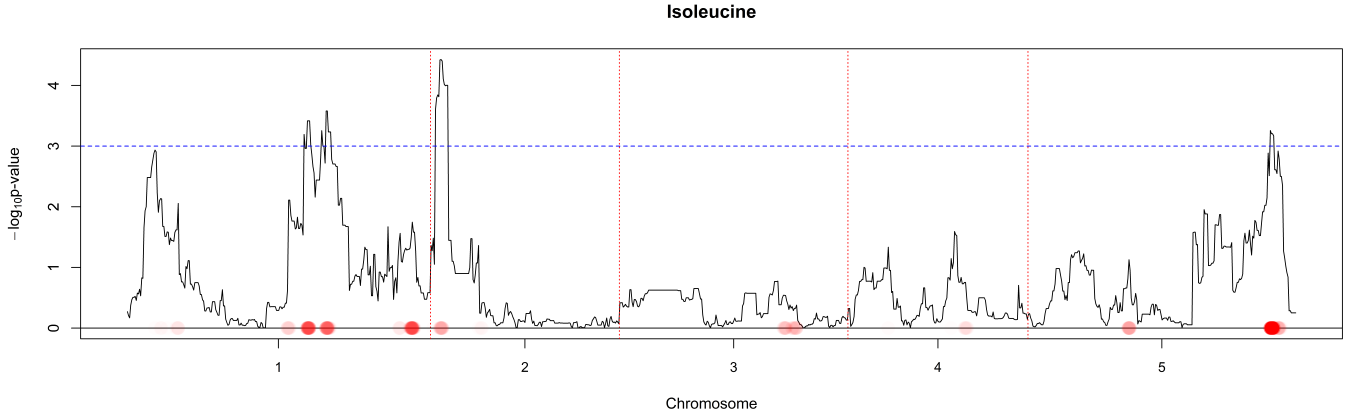
Isoleucine
−log10p-value
0
1
2
3
4
1
2
3
4
5
Chromosome

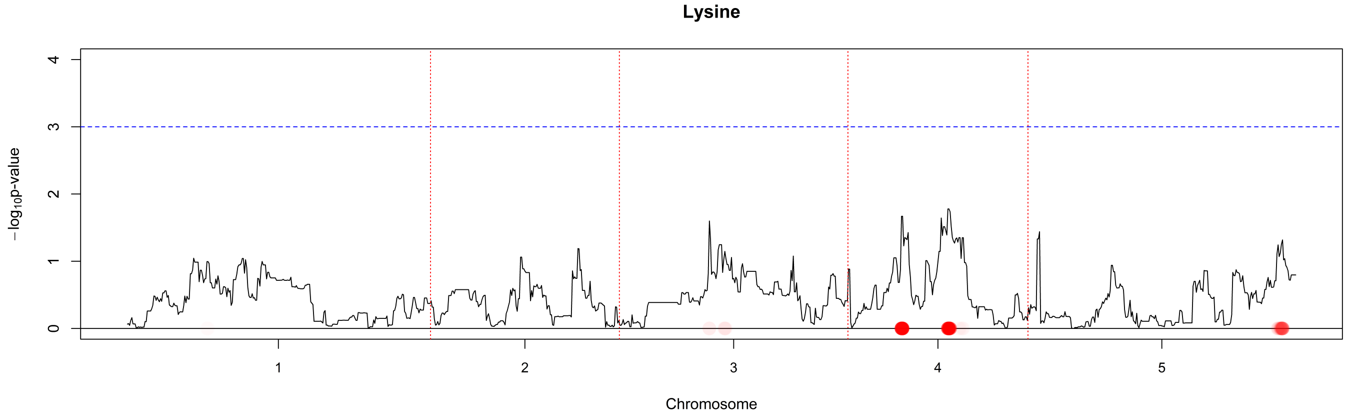
Lysine
−log10p-value
0
1
2
3
4
1
2
3
4
5
Chromosome

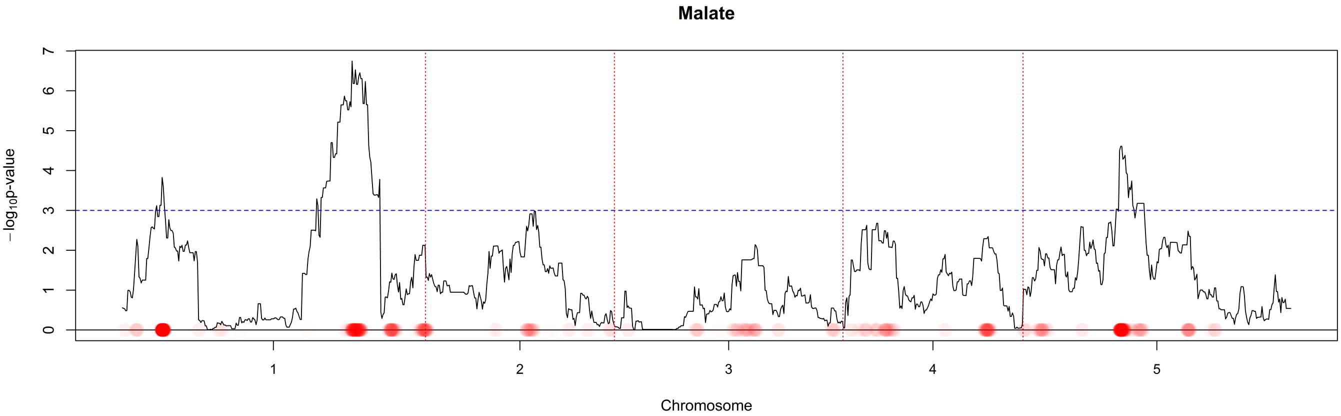
Malate
$-\log_{10}$p-value
Chromosome

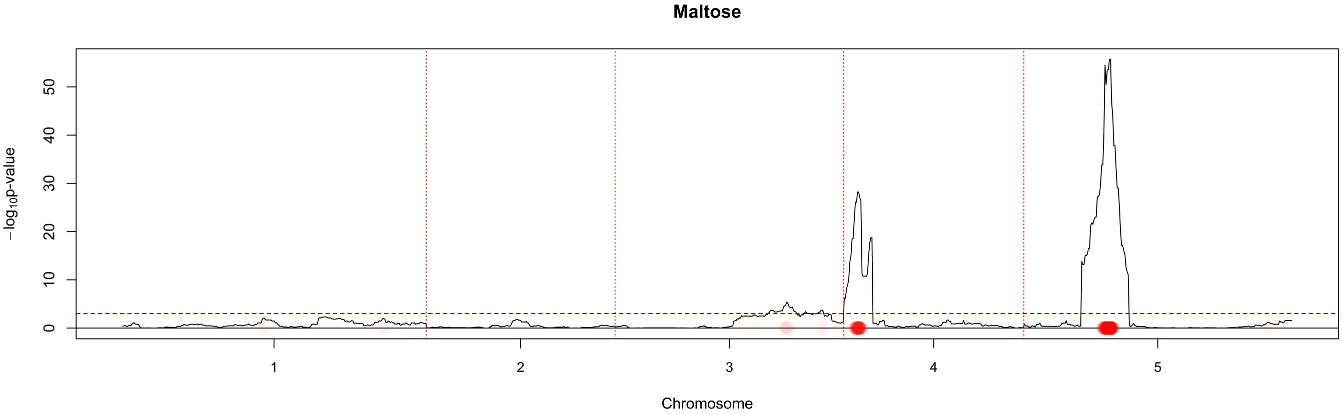
Maltose
$-\log_{10}$p-value
Chromosome

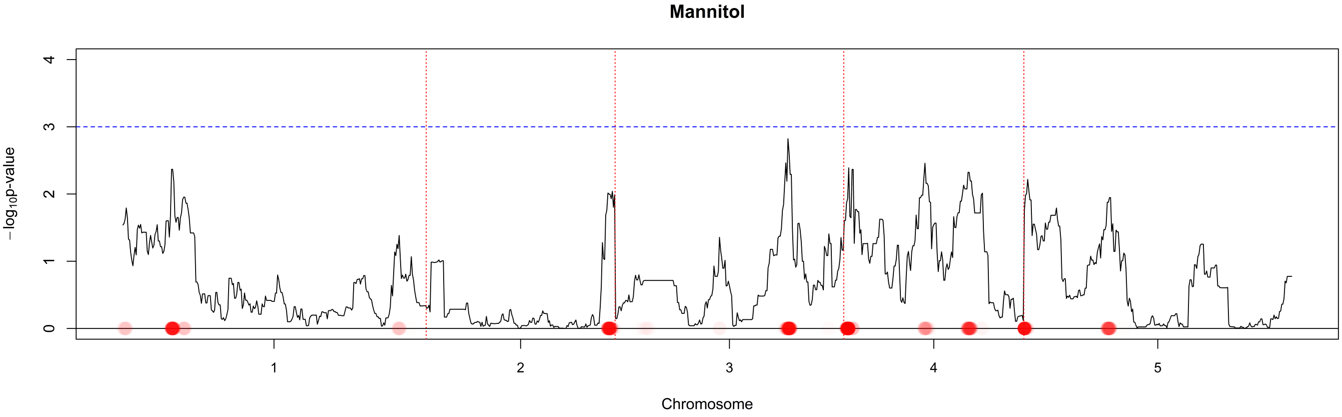
Mannitol
$-\log_{10}$p-value
Chromosome

Mannose

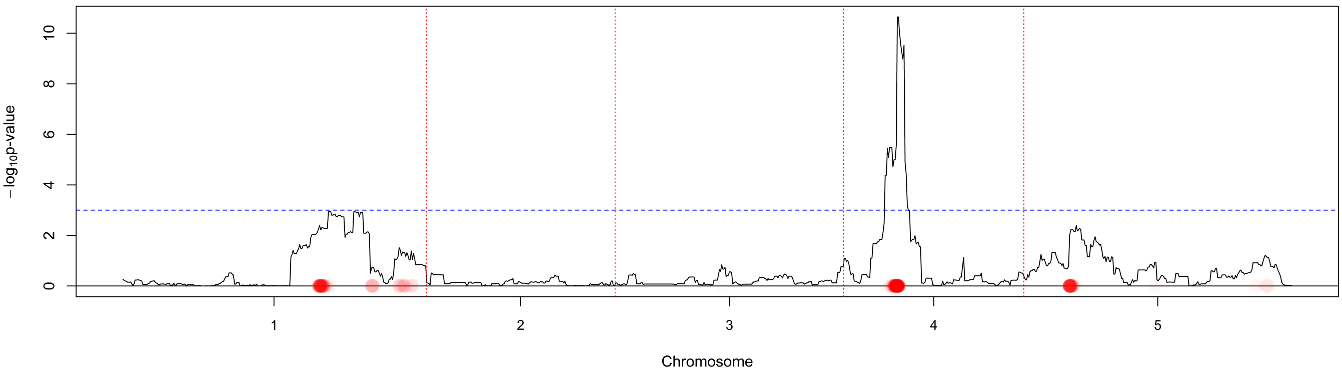

Methonione

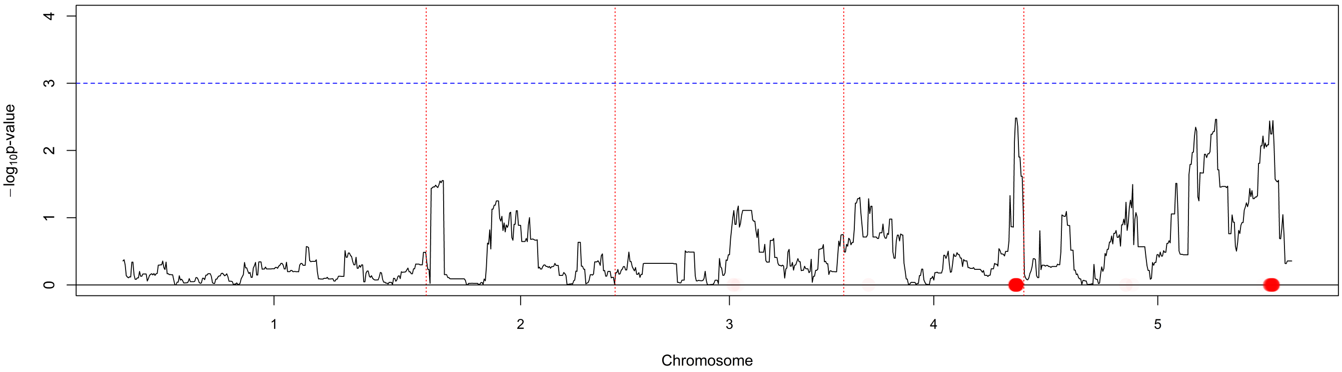

Monomethylphosphate

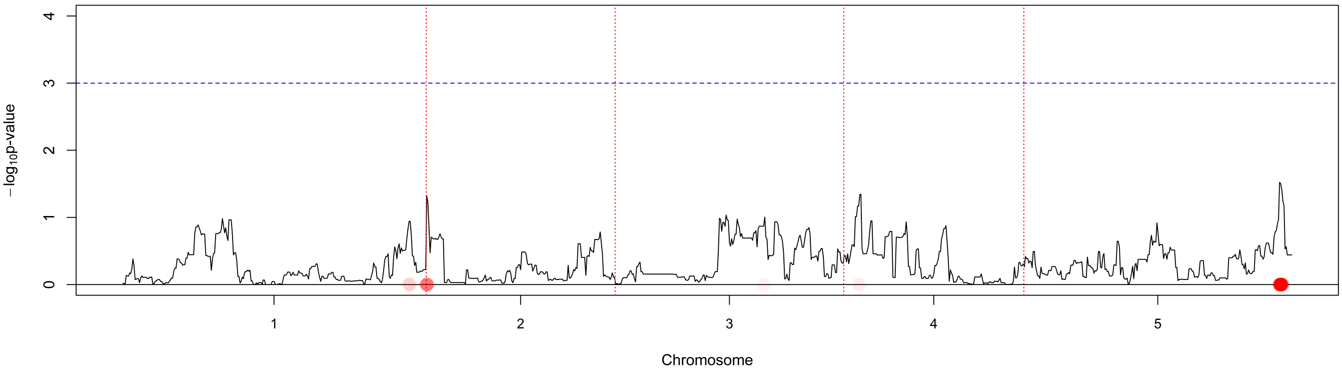

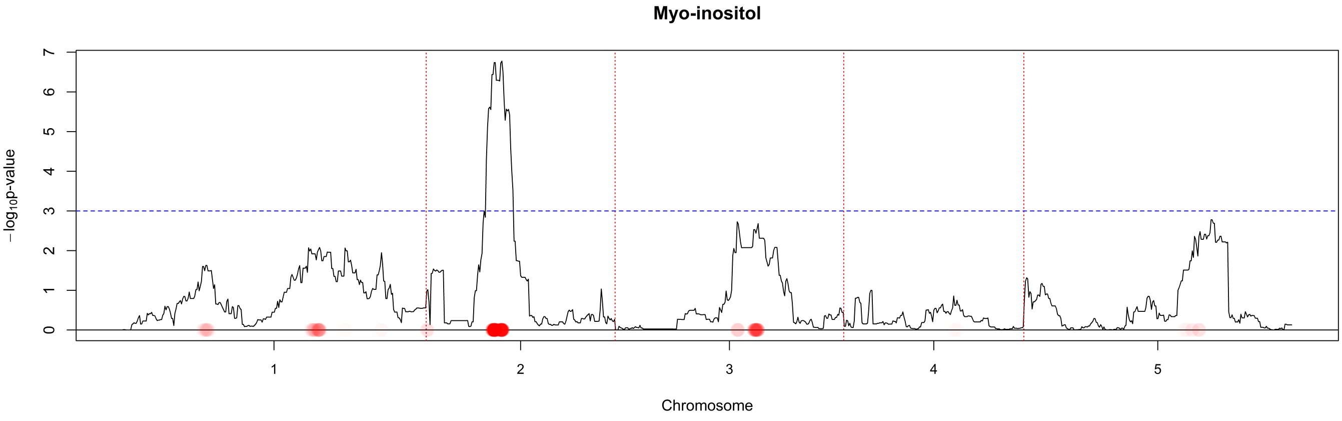
Myo-inositol
−log10p-value
0
1
2
3
4
5
6
7
1
2
3
4
5
Chromosome

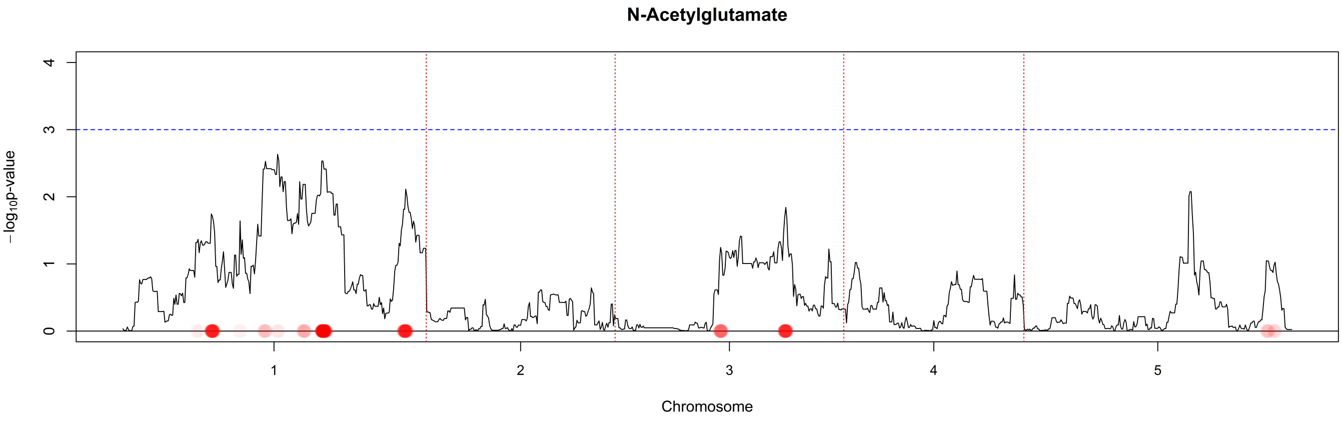
N-Acetylglutamate
−log10p-value
0
1
2
3
4
1
2
3
4
5
Chromosome

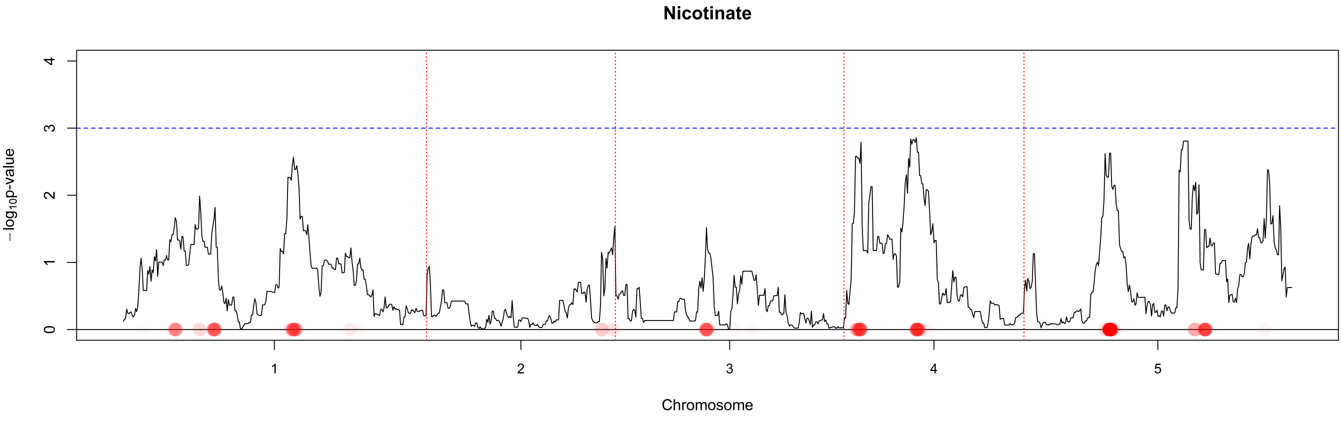
Nicotinate
−log10p-value
0
1
2
3
4
1
2
3
4
5
Chromosome

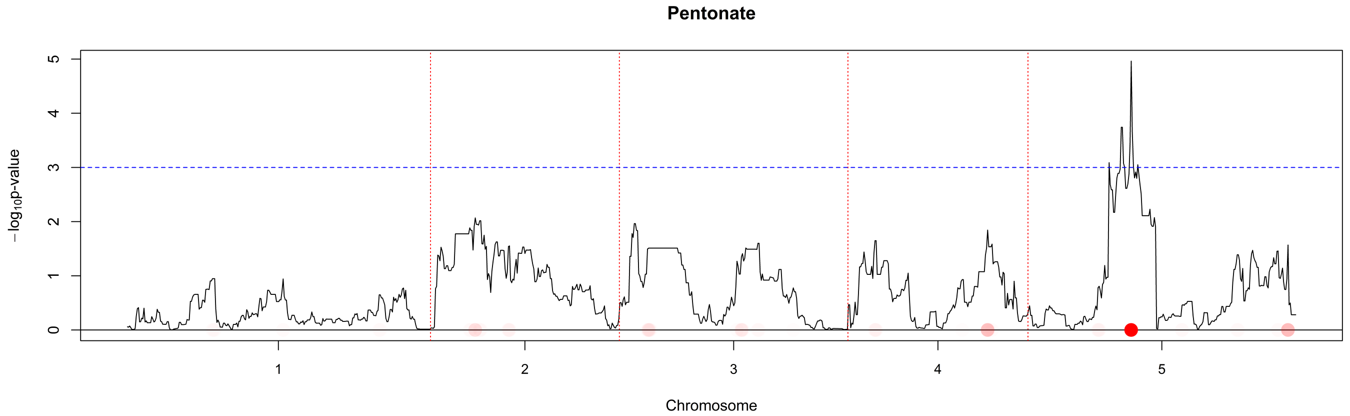
Pentonate
−log10p-value
Chromosome

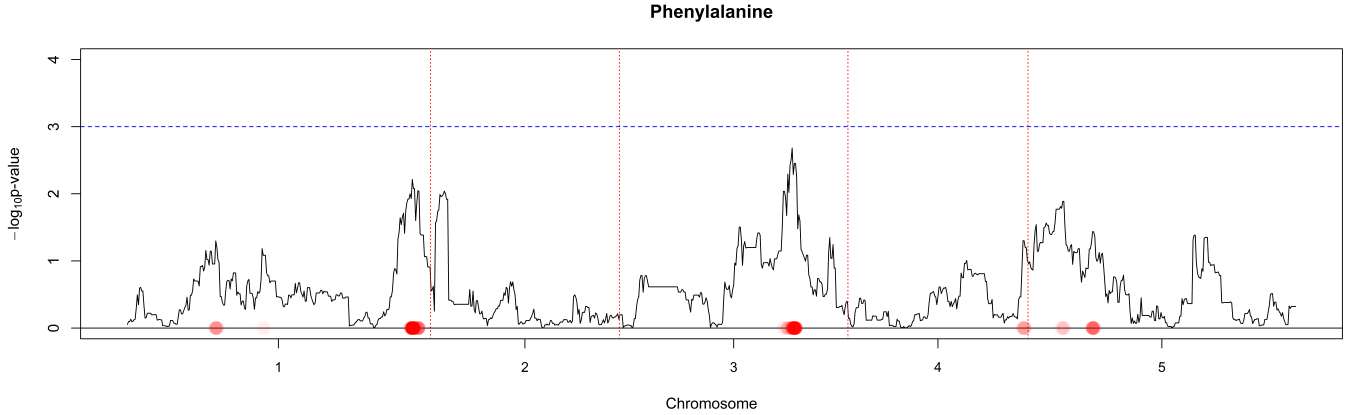
Phenylalanine
−log10p-value
Chromosome

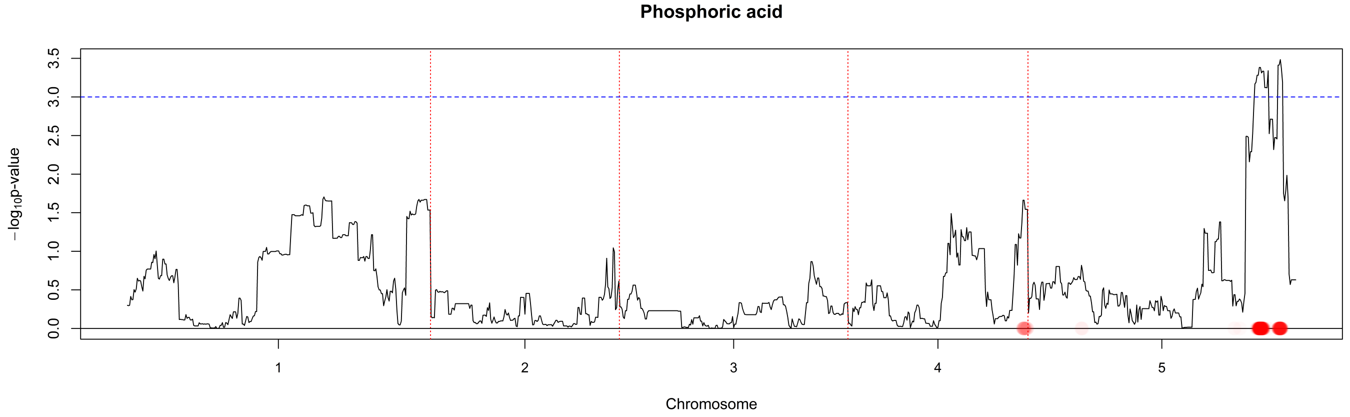
Phosphoric acid
−log10p-value
Chromosome

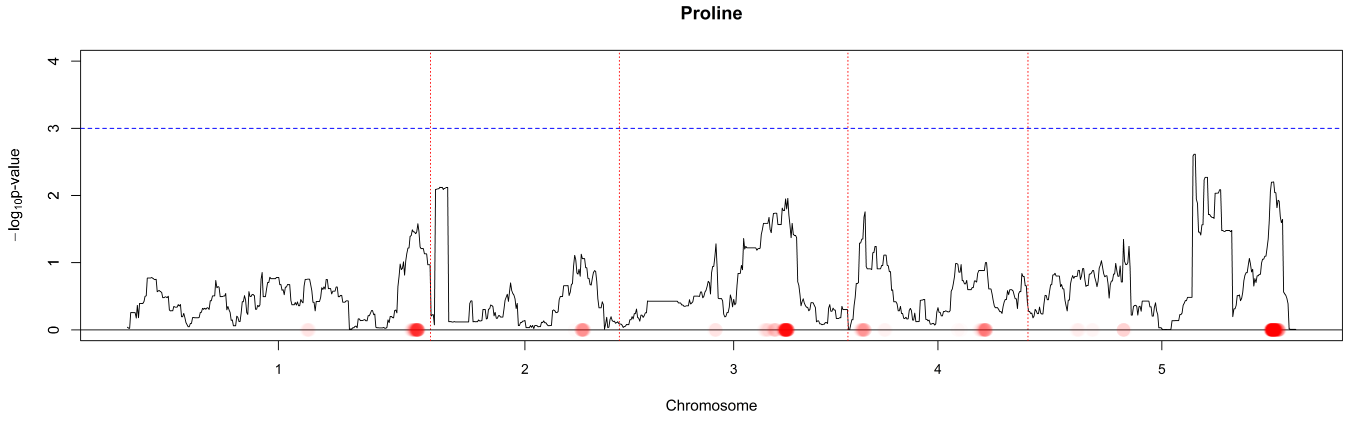
Proline
4
3
2
1
0
$-\log_{10}$p-value
1
2
3
4
5
Chromosome

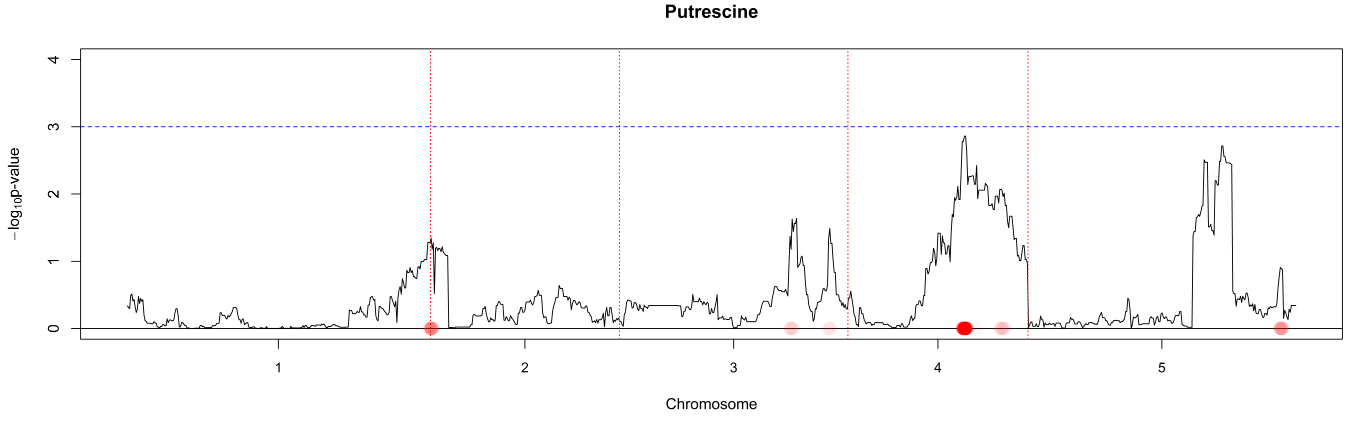
Putrescine
4
3
2
1
0
$-\log_{10}$p-value
1
2
3
4
5
Chromosome

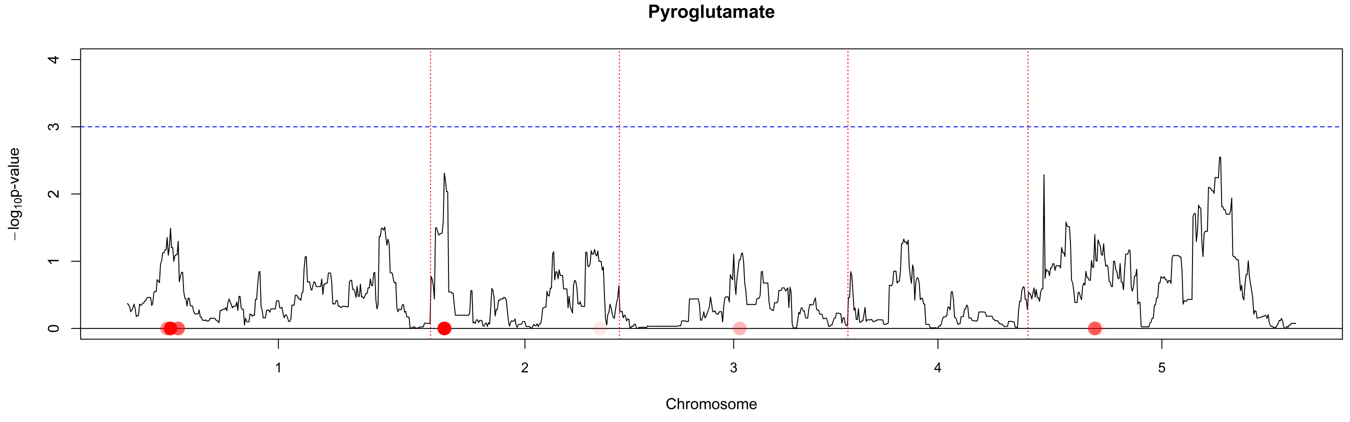
Pyroglutamate
4
3
2
1
0
$-\log_{10}$p-value
1
2
3
4
5
Chromosome

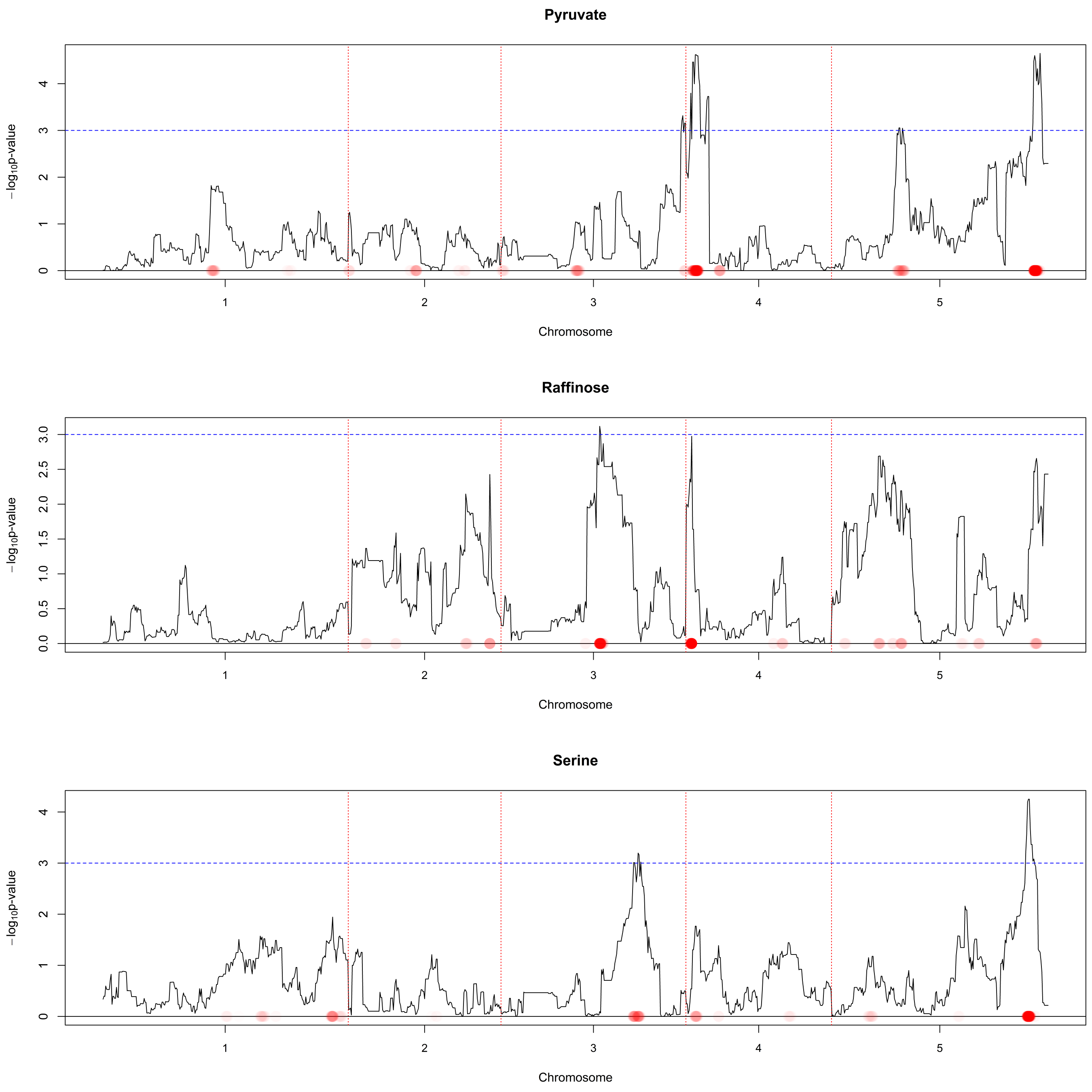

Pyruvate
$-\log_{10}$p-value
0
1
2
3
4
1
2
3
4
5
Chromosome
Raffinose
$-\log_{10}$p-value
0.0
0.5
1.0
1.5
2.0
2.5
3.0
1
2
3
4
5
Chromosome
Serine
$-\log_{10}$p-value
0
1
2
3
4
1
2
3
4
5
Chromosome

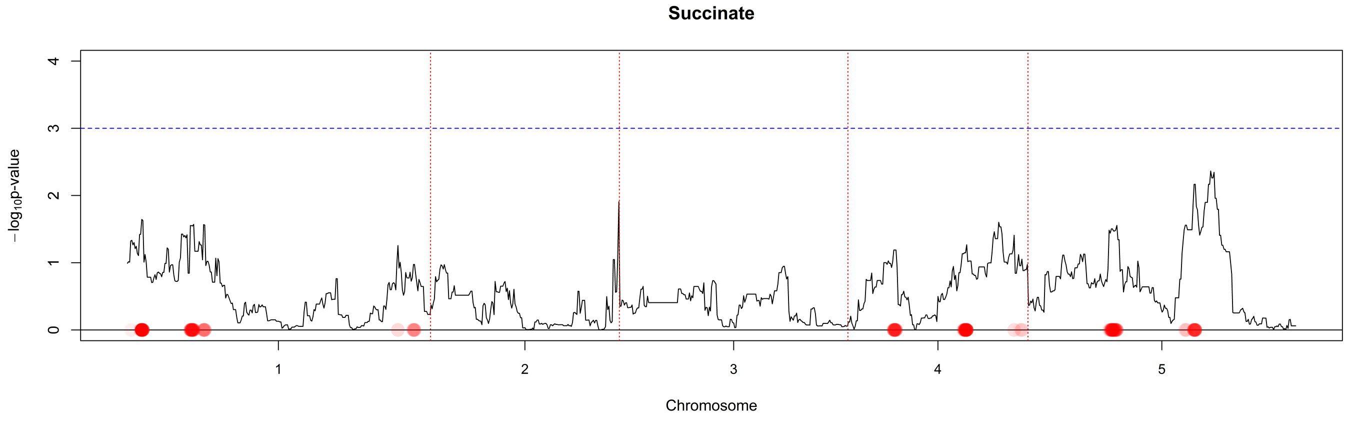
Succinate
−log10p-value
0
1
2
3
4
1
2
3
4
5
Chromosome

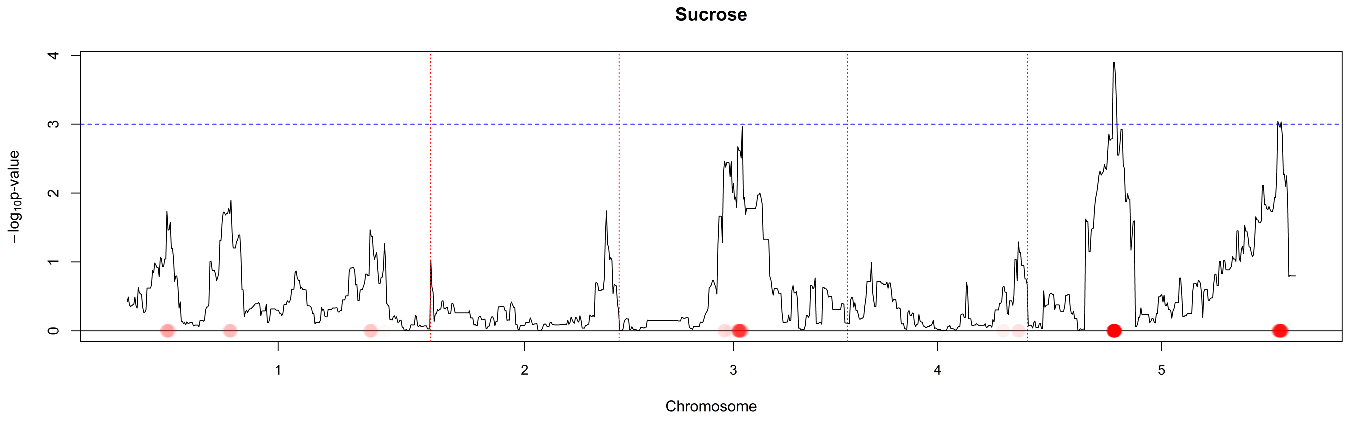
Sucrose
−log10p-value
0
1
2
3
4
1
2
3
4
5
Chromosome

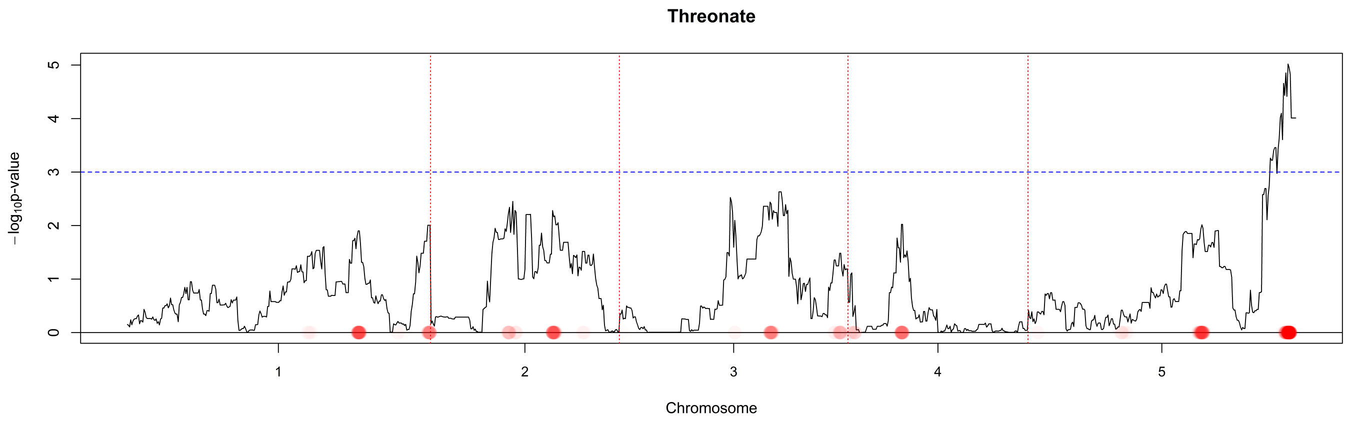
Threonate
−log10p-value
0
1
2
3
4
5
1
2
3
4
5
Chromosome

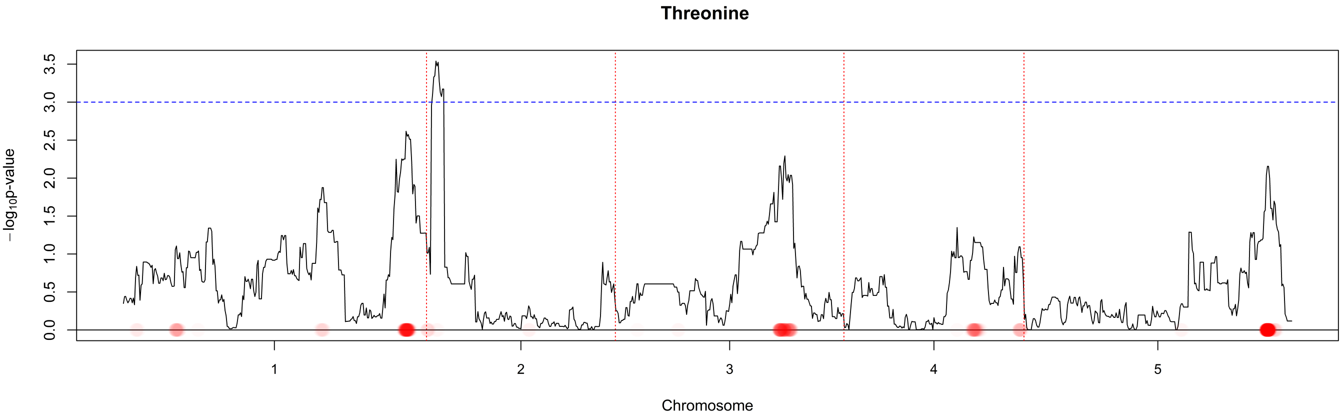
Threonine
−log10p-value
0.0
0.5
1.0
1.5
2.0
2.5
3.0
3.5
1
2
3
4
5
Chromosome

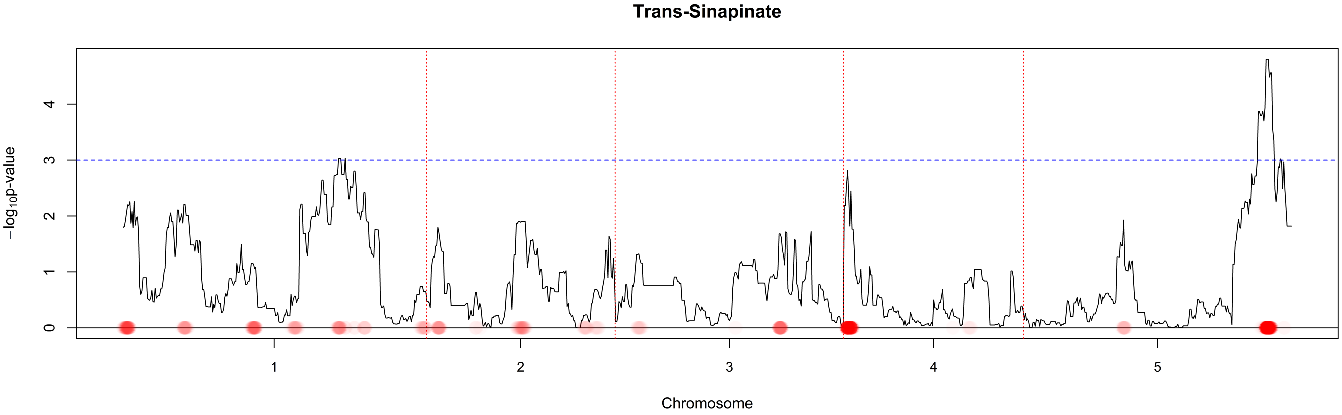
Trans-Sinapinate
−log10p-value
0
1
2
3
4
1
2
3
4
5
Chromosome

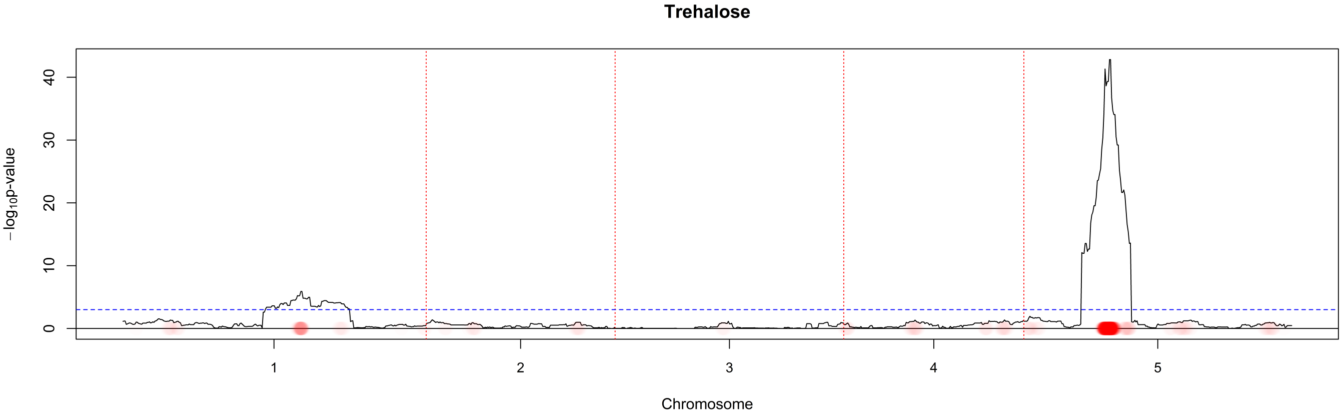
Trehalose
−log10p-value
0
10
20
30
40
1
2
3
4
5
Chromosome

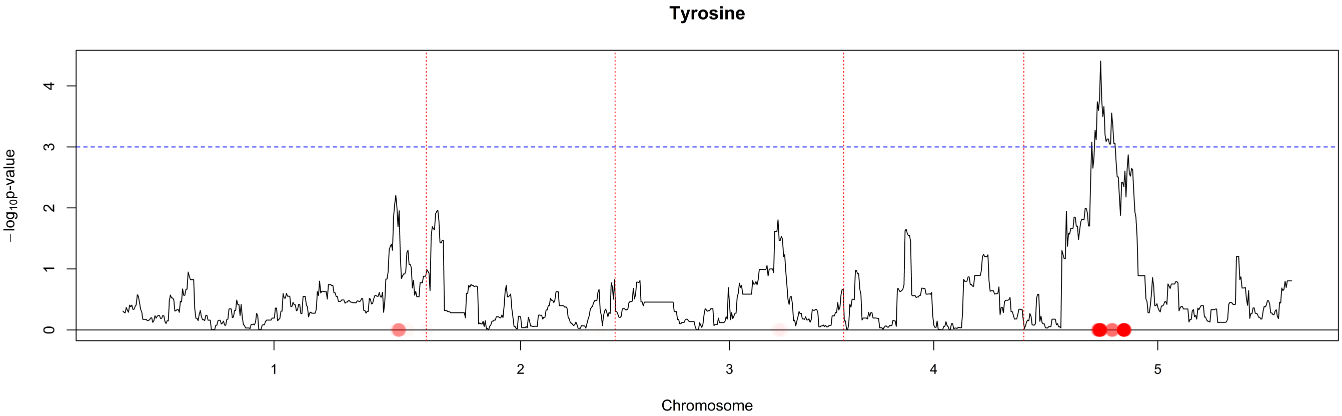
Tyrosine
−log10p-value
0
1
2
3
4
1
2
3
4
5
Chromosome

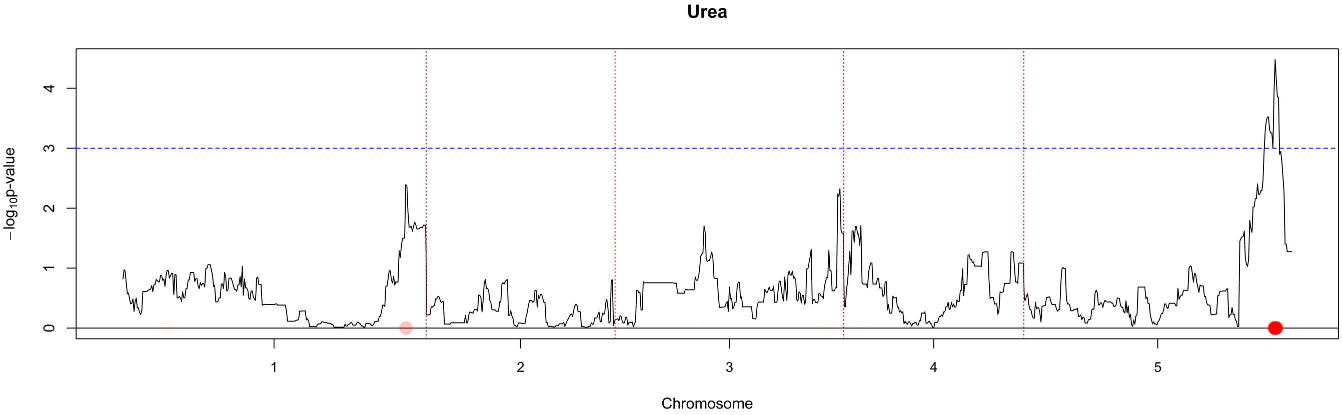
Urea
−log10p-value
0
1
2
3
4
1
2
3
4
5
Chromosome

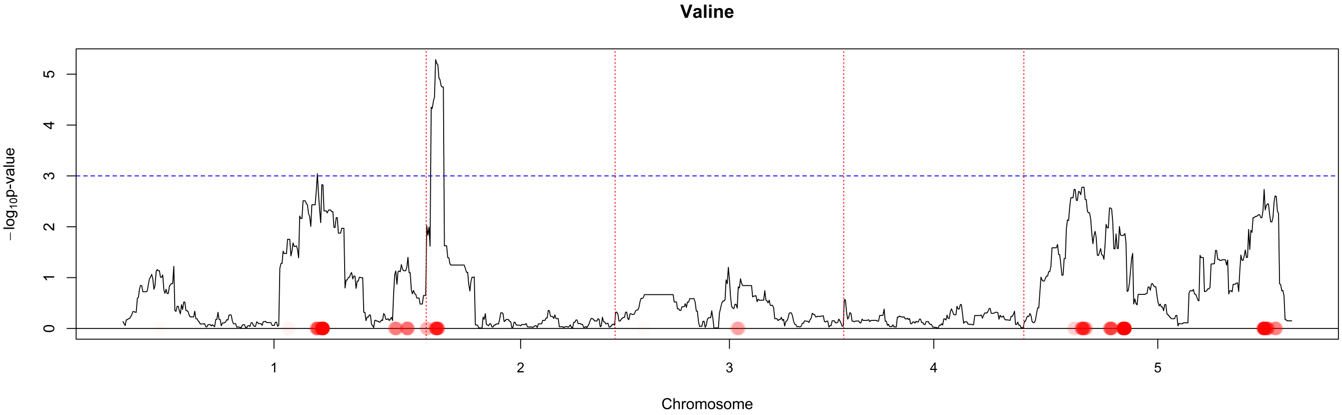
Valine
−log10p-value
0
1
2
3
4
5
1
2
3
4
5
Chromosome

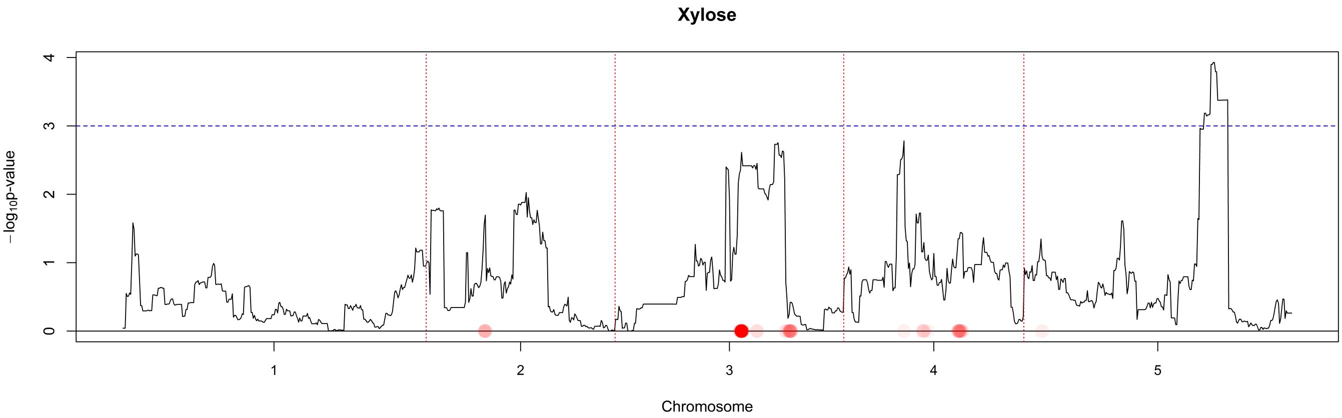
Xylose
−log10p-value
0
1
2
3
4
1
2
3
4
5
Chromosome
